# Nicotinic Acid-Mediated Modulation of Metastasis-Associated Protein 1 Methylation and Inflammation in Brain Arteriovenous Malformation

**DOI:** 10.3390/biom13101495

**Published:** 2023-10-08

**Authors:** Xinpeng Deng, Shengjun Zhou, Ziliang Hu, Fanyong Gong, Junjun Zhang, Chenhui Zhou, Wenting Lan, Xiang Gao, Yi Huang

**Affiliations:** 1 Department of Neurosurgery, The First Affiliated Hospital of Ningbo University, Ningbo 315010, China; 22118158@zju.edu.cn (X.D.); fyyzhoushengjun@nbu.edu.cn (S.Z.); huzilianhao8@163.com (Z.H.); gfyclimb@foxmail.com (F.G.); nbdxfsdyyyzhangjj@163.com (J.Z.); fyyzhouchenhui@nbu.edu.cn (C.Z.); 2 Department of Neurosurgery, Ningbo Hospital of Zhejiang University, Ningbo 315010, China; 3 Cixi Biomedical Research Institute, Wenzhou Medical University, Cixi 315302, China; 4 Department of Radiology, The First Affiliated Hospital of Ningbo University, Ningbo 315010, China; lwt_hh@163.com; 5 Key Laboratory of Precision Medicine for Atherosclerotic Diseases of Zhejiang Province, Ningbo 315010, China

**Keywords:** metastasis-associated protein 1, DNA methylation, inflammation, brain arteriovenous malformation, nicotinic acid, NF-κB

## Abstract

We explored metastasis-associated protein 1 (*MTA1*) promoter methylation in the development of brain arteriovenous malformation (BAVM). The clinical data of 148 sex- and age-matched BAVMs and controls were collected, and the *MTA1* DNA methylation in peripheral white blood cells (WBC) was assessed by bisulfite pyrosequencing. Among them, 18 pairs of case–control samples were used for WBC mRNA detection, 32 pairs were used for WBC MTA1 protein measurement, and 50 pairs were used for plasma inflammatory factor analysis. Lipopolysaccharide (LPS) treatment was used to induce an inflammatory injury cell model of human brain microvascular endothelial cells (BMECS). 5-Aza-2′-deoxycytidine (5-AZA), nicotinic acid (NA), and *MTA1* siRNAs were used in functional experiments to examine BMECS behaviors. RT-qPCR, Western blot, and ELISA or cytometric bead arrays were used to measure the expression levels of MTA1, cytokines, and signaling pathway proteins in human blood or BMECS. The degree of *MTA1* promoter methylation was reduced in BAVM compared with the control group and was inversely proportional to MTA1 expression. Plasma ApoA concentrations in BAVM patients were significantly lower than those in controls and correlated positively with *MTA1* promoter methylation and negatively with MTA1 expression. The expression of cytokine was markedly higher in BAVM than in controls. Cell experiments showed that 5-AZA decreased the methylation level of *MTA1* and increased the expression of MTA1 protein. LPS treatment significantly increased cytokine concentrations (*p* < 0.05). NA and MTA1 silencing could effectively reverse the LPS-mediated increase in IL-6 and TNF-α expression through the NF-κB pathway. Our study indicated that NA may regulate MTA1 expression by affecting promoter DNA methylation, improve vascular inflammation through the NF-κB pathway, and alleviate the pathological development of BAVM.

## 1. Introduction

Brain arteriovenous malformation (BAVM) is a disease in which the connections between arteries and veins are disordered, owing to congenital hypoplasia or acquired adverse factors impacting cerebral blood vessels [1]. Vascular remodeling, hemodynamic imbalance, and inflammation would have crucial roles in the pathophysiological mechanism of BAVM [2]. We observed that inflammatory factors can induce matrix metalloproteinase (MMP)-3 and MMP-9 expression and activity in the mouse brain, leading to brain endothelial cell proliferation and migration, and increasing the chance of BAVM rupture and bleeding [3]. This study revealed that a large number of neutrophils and macrophages gather around the vascular malformation, and an increased local expression of inflammatory factors and related genes may participate in the progression of BAVM disease to vascular rupture [4,5].

In higher eukaryotes, DNA methylation serves as a vital mechanism for epigenetic regulation [6]. The level of DNA methylation in the peripheral blood of patients with cardiovascular and cerebrovascular diseases is often affected by factors including stress, blood lipids, smoking, and drinking [7]. DNA methylation in promoters results in transcriptional silencing of protein-coding genes, thereby impacting their protein function in humans [8]. Researchers demonstrated that changes in local hemodynamics within a BAVM vascular mass might influence cellular metabolism, triggering epigenetic factors in endothelial cells [9]. Thomas et al. [10] observed abnormal DNA methylation signatures in promoters of genes related to vascular development in their study of brain BAVMs tissue. Further, they believed that the presence of multiple alterations in DNA methylation and histone modification in genes linked to vascular development implicated these genes in BAVM development. In addition, Joo et al. observed a significant difference in methylation between arterial and venous endothelial cells, which might be linked to variations in function of different vascular endothelial cells [11]. Thus, abnormal epigenetic modifications in the endothelial cell genome may lead to abnormal arterial or venous phenotypes [9].

Metastasis-associated protein 1 (MTA1) has been considered as a factor involved in tube formation [12]. Studies have reported that *MTA1* has played a crucial regulatory role in blood vessel formation. Kai et al. [13] observed that silencing *MTA1* markedly inhibited cell invasion and angiogenic activity, whereas tumor cells expressing MTA1 had a higher proliferative index, released more VEGF, and exhibited greater vascularization. *MTA1* was also found to promote angiogenesis by stabilizing hypoxia-inducible factor-1α [14]. In addition, studies have indicated that *MTA1* has an essential function in the inflammatory response. It regulates lipopolysaccharide (LPS)-induced pro-inflammatory cytokines as targets and components of NF-κB signaling [15]. In related studies of acute lung injury, MTA1 was reported to regulate local inflammatory response and the apoptosis of alveolar epithelial cells [16]. Knocking down *MTA1* using siRNA was found to reduce the 4-HNE-induced IL-6, TNF-α, and IL-1β levels (these cytokines are considered proinflammatory) [17]. Researchers have demonstrated the participation of *MTA1* in NOTCH, NF-κB, and TGF-β pathways in several diseases [18,19]. Importantly, the TGF-β, NOTCH, and Ras-MAPK pathways are all related to the occurrence and development of BAVM [20,21,22]. To date, the relationship between *MTA1* and BAVM remains unclear. We thus hypothesized that *MTA1* methylation fosters the accumulation of cytokines, including interleukins, triggered by the NF-κB pathway by regulating MTA1 protein translation, eventually leading to BAVM.

We conducted a case–control investigation to study the potential associations between *MTA1* methylation, MTA1 expression, cytokine levels, and BAVM risk. Cell experiments were also performed to explore the functions of nicotinic acid (NA) and other factors on *MTA1* methylation and expression, and to assess the impact of *MTA1* hypomethylation and *MTA1* gene silencing on vascular inflammatory factors though the NF-κB pathway.

## 2. Materials and Methods

**Figure 1:**
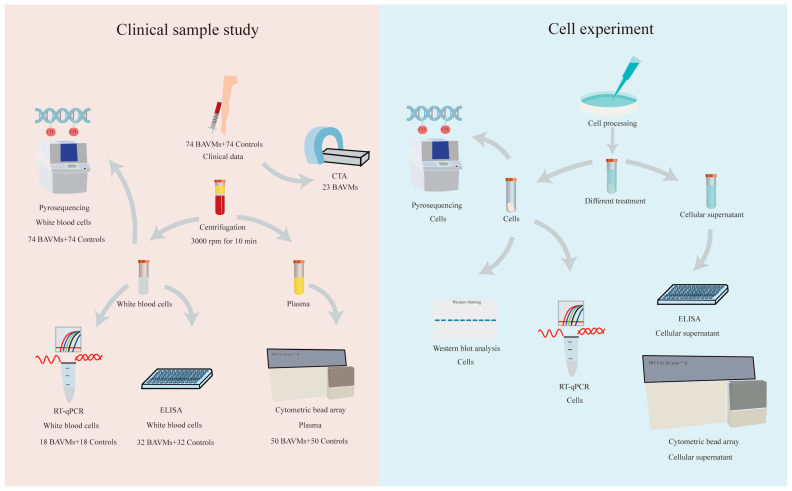
Flowchart of this study. (**left**) The flowchart of clinical sample studies. (**right**) The flowchart of cell experiment.

Seventy-four patients with BAVM and seventy-four patients with trigeminal neuralgia (regarded as the controls) were enrolled in this study. The research process of clinical samples is shown in Figure 1 and Supplementary Table S1. All subjects were inpatients in the neurosurgery department of the First Affiliated Hospital of Ningbo University and were diagnosed according to standard definitions using magnetic resonance imaging or angiography. Subjects with a history of cardiovascular, severe liver, or kidney disease were excluded. Diagnoses were made independently by at least two neurosurgeons. Blood samples were collected from all patients on the first day after admission and processed by the same researcher. The Ethics Committee of the First Affiliated Hospital of Ningbo University approved this study, and written informed consent was obtained from all participants.

### 2.1. Biochemical Analysis

Approximately 8 mL blood samples were collected from each volunteer in two tubes treated with 3.2% sodium citrate within 12 h of fasting. To obtain plasma and peripheral white blood cells (WBC), samples were centrifuged at 3000 rpm for 10 min at 4 °C, and then the upper plasma layer and the middle WBC layer were carefully pipetted into separate centrifuge tubes [23]. Isolated samples were stored temporarily at 4 °C and subsequently at −80 °C for extended preservation. The expressions of total cholesterol (TC), triglycerides (TG), low-density lipoprotein (LDL), and high-density lipoprotein (HDL) were measured using an automatic biochemical analyzer (Olympus AU2700, Tokyo, Japan). ApoA, ApoB, and ApoE concentrations were estimated via the turbidimetric transmission method.

### 2.2. Cerebral Artery CT Angiography

For the computed tomography (CT) angiography (CTA), a 320-row volumetric CT machine (Toshiba Aquilion One, Japan) was used. All patients underwent CTA of the head and neck arteries. Patients were placed in a horizontal position with calm breathing and were instructed to avoid movement and swallowing. Sixty milliliters of iodine contrast reagent, at a concentration of 370 mg/mL, were administered using a double-barreled high-pressure syringe at a rate of 6 mL/s. Next, 20 mL normal saline was infused at a flow rate of 6.5 mL/s. Scanning parameters were as follows: tube voltage 120 kV, tube current 400 mAs, matrix 350 × 350, scanning layer thickness 16 × 0.75 mm, volume scanning (range: from the lower edge of the aortic arch to the cranial roof and the foot to the cephalic side as the scanning direction). Image postprocessing involved maximal intensity projection (MIP), curved planar reconstruction (CPR), and volume-rendering (VR) techniques.

### 2.3. Pyrosequencing

*MTA1* is located in Chr14:105,419,827–105,470,729 (GRCh38/hg38). For DNA methylation pyrosequencing, thirteen CpG dinucleotides located in the promoter area of the *MTA1* have been selected. We used the Qiagen genomic DNA extraction kit to extract the genomic DNA from the WBC, which is manufactured by Qiagen in Hilden, Germany. The bisulfite conversion was carried out using the Epi Tech DNA bisulfite Kit (Qiagen). The PyroMark Q24 (Qiagen) was utilized to measure DNA methylation degrees. The PCR primers were designed through Qiagen′s PyroMark Assay Design v.2.0 software. They included a pair of *MTA1* gene methylation-specific amplification primers and a *MTA1* gene methylation-specific sequencing primer. The nucleotide sequence of upstream primers of the *MTA1* gene methylation-specific amplification was shown as 5′-GGTGGGGGGAGGTTTATTTG-3′. The nucleotide sequence of downstream primer specifically amplified by *MTA1* gene methylation was shown as 5′-Biotin-CAACCCCAAAACTCCTACT-3′; the nucleotide sequence of the *MTA1* gene methylation-specific sequencing primer was shown as 5′-GGGGGAGGTTTATTTGTT-3′.

### 2.4. Quantitative Real-Time Polymerase Chain Reactions (RT-qPCR)

Among the included samples, 18 patients with BAVMs and 18 controls were selected for the detection of *MTA1* content from the WBC. The WBC RNA was extracted using TRIzol reagent (Invitrogen, Carlsbad, CA, USA). cDNA was obtained via the reverse transcription of RNA by a high-volume reverse transcription kit (TransGen Biotech, Beijing, China). Quantitative PCR was performed with a LightCycler 480 system from Roche (Mannheim, Germany), using SYBR Green SuperMix from TransGen Biotech in Beijing, China. The qPCR primers were designed using primer 6 software (Premier Biosoft, CA, USA). The primer sequences were as follows: *MTA1* forward primer, 5′-TACCTGGAGCGGGAGGATTTCTT-3′; and reverse primer, 5′-GCTTGTCTGTGAGTGGGTTGTGC-3′. ACTB was used as an internal reference [24], with the forward primer being 5′-AGCACAGAGCCTCGCCTT-3′ and the reverse primer being 5′-CATCATCCATGGTGAGCTGG-3′. The 2^−△△Ct^ method was used to calculate mRNA expression.

#### Western Blot Analysis

The proteins were separated via sodium dodecyl sulfate-polyacrylamide gel electrophoresis (SDS-PAGE) and transferred to polyvinylidene fluoride. The membranes were blocked for 1 h at room temperature with 5% milk and incubated with the indicated primary antibodies at 4 °C overnight. After washing with PBST three times for 30 min, the membranes were incubated with secondary antibodies for 1 h. The membranes were washed with PBST three times and visualized by chemiluminescence. We used the following antibodies for the experiments: p-RELA/NFκB p65 antibody (Cat: sc-136548, Santa Cruz Biotechnology, TX, USA), MTA1 (Cat: sc-373765, Santa Cruz), HRP-conjugated Affinipure Goat Anti-Rabbit IgG (Cat: SA00001-2, Proteintech, Wuhan, China), HRP-conjugated Affinipure Goat Anti-Mouse IgG (Cat: SA00001-1, Proteintech), and β-actin Antibody (Cat: AF7018, Affinity, Jiangsu, China). Band intensities were quantified by ImageJ and were normalized using β-actin as a housekeeping protein.

### 2.5. Enzyme-Linked Immunosorbent Assay (ELISA)

Sex- and age-matched groups of 32 patients with BAVMs and 32 controls were selected to measure WBC MTA1 protein expression. Cell supernatants were used to detect the expressions of IL-6 and TNF-α. The ELISA kits were used according to the manufacturer′s instructions to detect MTA1 protein levels (MULTISCIENCES, Hangzhou, China), IL-6 (E-EL-H6156, Elabscience, Wuhan, China), and TNF-α (E-EL-H0109c, Elabscience). A multifunctional microplate reader (Molecular Devices, CA, USA) was used to record the absorbance of each well at 450 nm.

### 2.6. Detection of Interleukin Using Cytometric Bead Array

Fifty BAVM patients and fifty controls were sampled on order to test cytokines concentrations including IL-2, IL-4, IL-6, TNF-α, IFN-γ, IL-10, and IL-17. The Human TH1/TH2/TH17 Subgroup Detection Kit (Flow Fluorescent Luminescence Method) (Jiangxi Saiji Biotechnology, Nanchang, China) was used to measure the concentration of the plasma cytokines. The process was as follows. Firstly, microsphere vortex for 20 s, mix thoroughly, centrifuge at 200× *g* for 5 min, discard the supernatant, and supplement with an equal volume of microsphere buffer. Incubate at room temperature in a light-protected environment for 15 min, vortex mix for 20–30 s, and add 25 μL microsphere to each experimental tube. After adding 25 μL of the test sample and 25 μL of fluorescent detection reagent, incubate in the dark for 2.5 h. After incubation, add 1 milliliter of PBS buffer to every well, centrifuge at 200× *g* for 5 min, discard the supernatant, and add 100 μL of PBS buffer to every well before detection on the machine (BD FACSCanto II Flow Cytometer, BD Biosciences, NJ, USA). In cell experiments, 2 × 10^5^ Cells after drug treatment were lysed with cell lysis solution RIPA (Lot. No.20230309, Solarbio, Beijing, China) and then centrifuged, and the separated supernatant was diluted five times and experimented with the above steps.

### 2.7. Cell Culture and Drug Treatment

In vitro studies were conducted using Brain Microvascular Endothelial Cells (BMVECS) (Cell Systems). BMVECS were cultured in endothelial cell medium (500 mL), FBS (25 mL), endothelial cell growth supplement (5 mL), and penicillin/streptomycin solution (5 mL, Lot. No. 36608, ScienCell Research Laboratories, San Diego, CA, USA). The medium was exchanged every 6–8 h. Lipopolysaccharide (LPS) (5 μg/mL) [25] (cat: GC19203, GlpBio, Montclair, CA) -treated cells (24 h) were used as an inflammatory cell model [26]. The overall flow of cell experiments is shown in Figure 1.

In order to explore the functions of DNA methylation on MTA1 expression, BMVECS were treated with 5-Aza-2′-deoxycytidine (5-AZA) (Cat. No. HY-A0004; MedChemExpress, NJ, USA), a commonly used DNA methyltransferase (DNMT) inhibitor. Cells were incubated with 5-AZA (2, 4 μM) for 3 days. Previous studies have shown that NA has a certain correlation with epigenetics [27]. We treated inflammatory cells with NA (300 μM) [28] (cat: GC13206, GlpBio, Montclair, CA, USA) and observed changes in DNA methylation and MTA1 expression. To verify the MTA1 functions in BMVECS, we used siRNA (Lot#L1619, Santa Cruz) to silence *MTA1* and observed changes in the NF-κB pathway and inflammatory cytokines. Detailed cell groupings are as follows: Control, LPS, LPS + NA, LPS + 5-AZA, LPS + Control siRNA, and LPS + MTA1 siRNA [25,28].

### 2.8. Transfection

In a six-well plate, 2 × 10^5^ BMVECS were seeded in each well. The cells were incubated at 37 °C in a CO_2_ incubator until the cell density reached 60–80% confluency. The cells were transfected with the control and *MTA1* siRNAs using Lipofectamine 3000 (Lot CN2541162, Thermo Fisher Scientific, MA, USA). Following incubation for 5–7 h, the culture medium was exchanged for subsequent experiments.

### 2.9. Statistical Analyses

SPSS (v. 25.0, Chicago, IL, USA) and GraphPad Prism (v. 7.0, CA, USA) were used for statistical analyses. The mean replacement method was used for missing data. Continuous variables were analyzed between groups using the Student′s t-test, whereas the Mann–Whitney U test was used for non-normal continuous variables. A one-way ANOVA was used for multi-group comparisons. Correlations among *MTA1* methylation, Spetzler-Martin classification, and clinical features were analyzed by Pearson or Spearman correlation coefficients. Propensity score-matching analysis was used to control for confounding factors. To assess the predictive value of the degree of *MTA1* methylation in relation to the BAVM risk, receiver operating characteristic (ROC) curves were utilized, with statistical significance designated as *p* < 0.05.

## 3. Results

### 3.1. Characteristics of Study Participants

**Figure 2:**
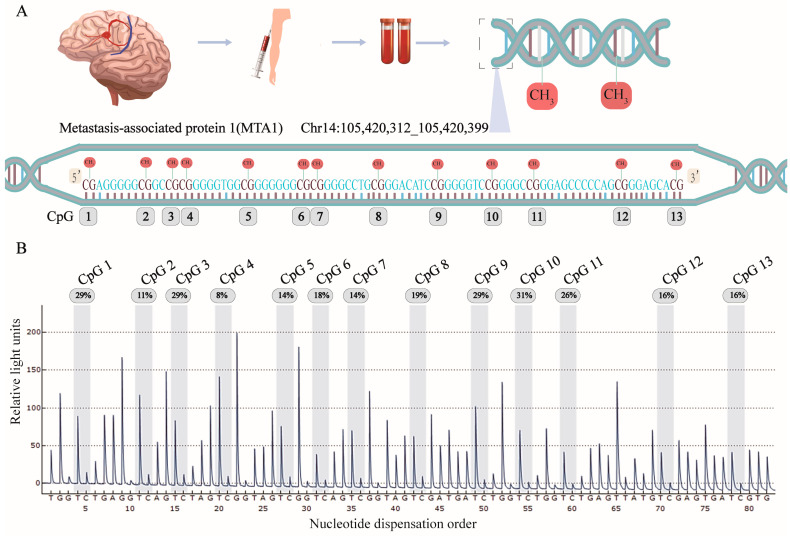
Location and analysis of 13 CpG sites in metastasis-associated protein 1 (MTA1). (**A**) *MTA1* target sequence and distribution of the 13 CpG sites. (**B**) A typical sequencing analysis of the 13 methylated sites. The percentage represents the degree of methylation.

**Figure 3:**
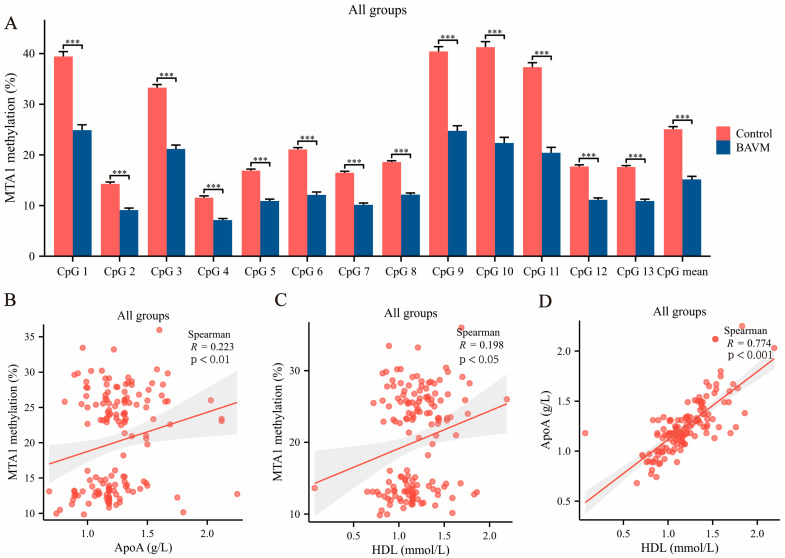
Correlation analysis between *MTA1* methylation and clinical data. (**A**) Methylation levels in brain arteriovenous malformation (BAVM) and controls among all participants (*n* = 74). (**B**) Correlation of *MTA1* methylation with APOA in all groups. (**C**) Correlation of *MTA1* methylation with HDL in all groups. (**D**) Correlation of HDL with APOA in all groups. Wilcoxon rank-sum test and Spearman correlation analysis were used to analyze data. Results are presented as mean ± SEM. ***: *p* < 0.001.

Seventy four BAVM patients (37 males and 37 females, mean age 49.70 ± 12.18 years) and 74 control patients (37 males and 37 females, mean age 52.99 ± 8.35 years, *p* > 0.05, Table 1) participated in this study. Clinical features, including ApoA, Lpa, and ApoE, were statistically different between the BAVM patients and the controls (*p* < 0.05, Table 1). No statistically significant differences were observed in TG, TC, HDL, LDL, or ApoB levels between the BAVM patients and the controls (*p* > 0.05, Table 1). The locations of CpG1, CpG2, CpG3, CpG4, CpG5, CpG6, CpG7, CpG8, CpG9, CpG10, CpG11, CpG12, and CpG13 in the *MTA1* fragment (Chr14:105,420,312–105,420,399, GRCh38/hg38) are shown in Figure 2. Compared to the controls, *MTA1* methylation degrees were markedly reduced in BAVM patients (*p* < 0.001, Figure 3A). Separate analyses by sex revealed that methylation was markedly reduced in the BAVM group compared to controls among males (*p* < 0.001, Supplementary Figure S1A) and females (*p* < 0.001, Supplementary Figure S1B). Since environmental factors can affect DNA methylation, we performed subgroup analyses to assess effects of smoking, alcohol consumption, and hyperlipidemia. Our study findings revealed a statistically significant decrease in *MTA1* methylation among smokers compared to nonsmokers but found no statistical difference among alcohol drinkers or patients with hyperlipidemia. Among all subjects, the degree of DNA methylation among the 13 CpG sites correlated positively (*p* < 0.05, Supplementary Figure S1C–E). *MTA1* mean methylation correlated positively with ApoA levels (*p* = 0.006, Figure 3B) in all groups. In addition, HDL concentration correlated positively with the mean *MTA1* methylation level (*p* = 0.016, Figure 3C) and ApoA level (*p* < 0.001, Figure 3D) in all the participants.

### 3.2. Cerebral Artery CT Angiography

**Figure 4:**
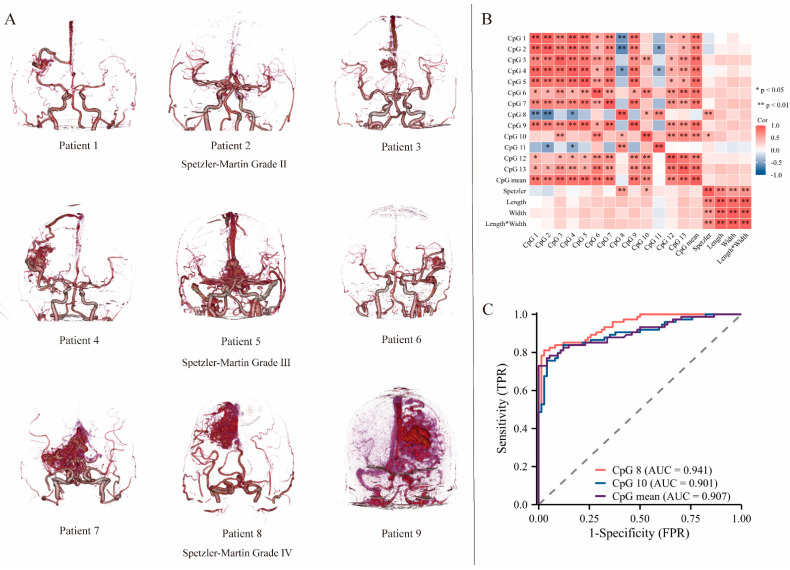
Analysis of correlation between *MTA1* methylation status and BAVM clinical grade. (**A**) Classification of computed tomography angiography (CTA) images from BAVM patients. (**B**) Correlation between Spetzler grade and *MTA1* methylation levels (*n* = 23). (**C**) Receiver operating characteristic (ROC) curves of *MTA1* methylation in BAVM patients. Spearman correlation analysis is used for data analysis.

Using the head and neck CTA imaging data of the 23 patients with BAVMs, we graded the patients according to the Spetzler–Martin classification criteria [29]. The CTA image classification of the patients is shown in Figure 4A. The degree of *MTA1* methylation at CpG 8 and CpG 10 correlated positively with the BAVM Spetzler classification (*p* < 0.05; Figure 4B). Additionally, receiver operating characteristic (ROC) curve analysis demonstrated that *MTA1* methylation had a significant diagnostic value for BAVM (CpG 8 methylation, area under the curve (AUC) = 0.941; CpG 10 methylation, AUC = 0.901; and *MTA1* methylation, AUC = 0.907, Figure 4C).

### 3.3. mRNA and Protein Expression

**Figure 5:**
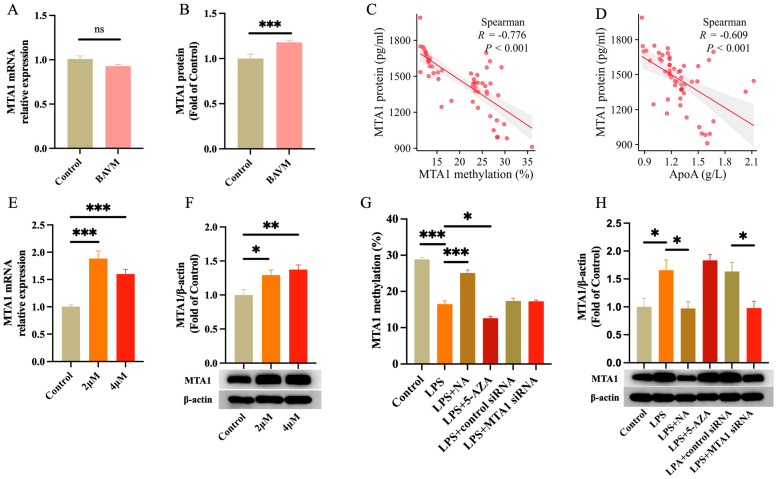
MTA1 expression analysis in clinical samples and cell experiments. (**A**) *MTA1* mRNA levels in clinical samples (*n* = 18). (**B**) ELISA detection of MTA1 protein expression differences in peripheral white blood cells (*n* = 32). (**C**) Correlation analysis between MTA1 methylation and protein level. (**D**) Correlation analysis between MTA1 expression and ApoA concentration. (**E**) 5-AZA (2 μM, 4 μM) treatment results in markedly increased levels of *MTA1* mRNA. (**F**) 5-AZA (2 μM, 4 μM) treatment visibly elevated the level of MTA1 protein (*n* = 3). (Original images can be found in Supplementary Materials). (**G**) The level of *MTA1* methylation in different cell treatment groups (*n* = 3). (**H**) The concentration of MTA1 in different cell treatment groups (*n* = 6). (NA: nicotinic acid; 5-AZA: 5-Aza-2′-deoxycytidine). Data (**A**,**B**) were analyzed using an unpaired t-test. Data (E-H) were analyzed through a one way ANOVA. Results are presented as mean ± SEM. *: *p* < 0.05, **: *p* < 0.01, ***: *p* < 0.001.

**Figure 6:**
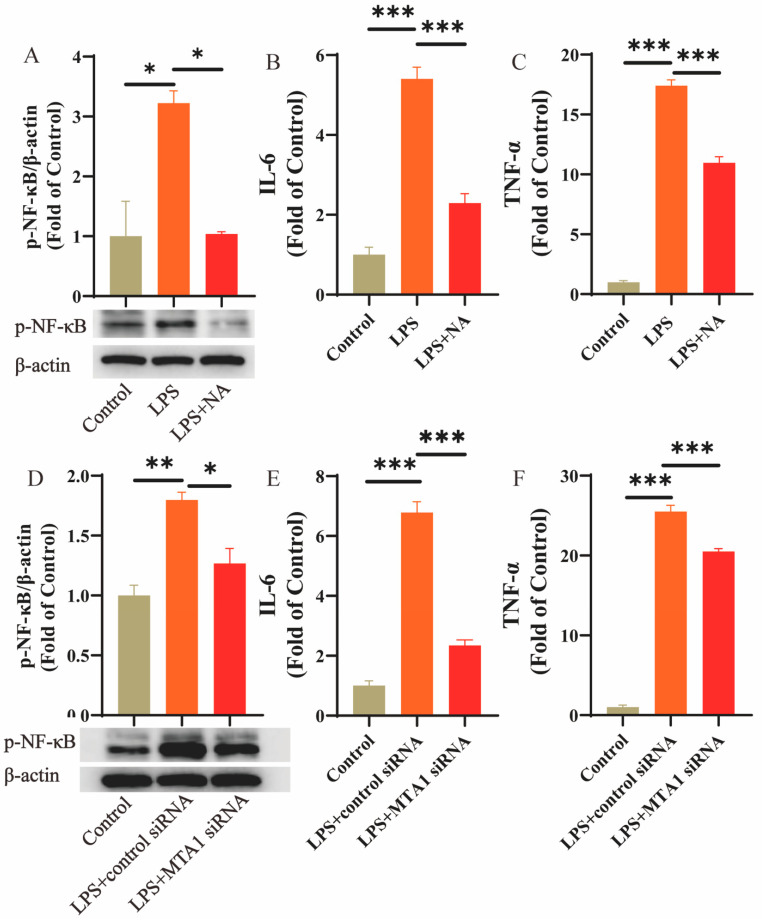
Changes in NF-κB pathway in cell function experiments. Changes in the expression of phosphorylated NF-κB (**A**) (*n* = 3) (Original images can be found in Supplementary Materials), IL-6 (**B**) (*n* = 6), and TNF-α (**C**) (*n* = 6) after LPS and NA treatment. Changes in the expression of phosphorylated NF-κB (**D**) (*n* = 3), IL-6 (**E**) (*n* = 6), and TNF-α (**F**) (*n* = 6) after *MTA1* siRNA treatment. Multi-group comparisons were processed via one way ANOVA. Results are presented as mean ± SEM. *: *p* < 0.05, **: *p* < 0.01, ***: *p* < 0.001.

No statistical difference (*p* > 0.05, Figure 5A) was observed in the *MTA1* mRNA expression in BAVM patients and controls. ELISA experiments revealed a marked elevation of MTA1 protein levels in BAVM patients (*p* < 0.05, Figure 5B). MTA1 protein concentrations correlated negatively with the average methylation level (*p* < 0.05, Figure 5C) and ApoA concentration (*p* < 0.05, Figure 5D). In cell experiments, in contrast to the blank group, the contents of both the *MTA1* mRNA and protein were markedly elevated in the experimental group after 5-AZA (2 μM, 4 μM) treatment (*p* < 0.05, Figure 5E,F). The *MTA1* methylation levels of six groups, Control, LPS, LPS + NA, LPS + 5-AZA, LPS + Control siRNA, and LPS + MTA1 siRNA, were detected, respectively. The experimental data showed that NA can increase the methylation level of *MTA1*, while 5-AZA and LPS can decrease the methylation level of MTA1 (*p* < 0.001, Figure 5G). The Western blot results demonstrated that *MTA1* content was elevated in the LPS group compared to that in the controls (*p* < 0.05, Figure 5H), and MTA1 expression was markedly reduced in the LPS + NA group compared to that in the LPS group. *MTA1* siRNA treatment significantly inhibited MTA1 expression in the LPS + MTA1 siRNA group compared with that in the LPS + control siRNA group. However, no significant difference has been observed between the LPS + 5-AZA and LPS groups. As shown in Figure 6, the cell function experiments showed that the expressions of phosphorylated NF-κB, IL-6, and TNF-α in the LPS group were significantly higher than that in the control group and were effectively reduced after NA treatment (Figure 6A–C). Similar results showed that the expression levels of phosphorylated NF-κB, IL-6, and TNF-α in the LPS + MTA1 siRNA group were also significantly lower than the LPS + Control siRNA group (Figure 6D–F).

### 3.4. Cytokine Levels

**Figure 7:**
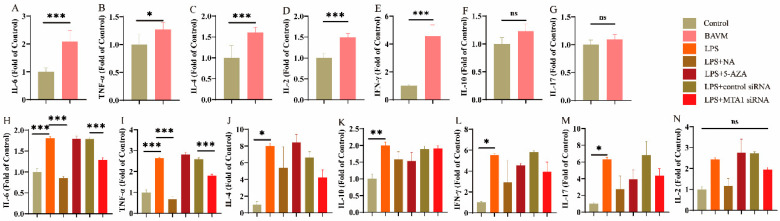
Analysis of cytokine levels in clinical samples. (**A**–**G**) IL-6, TNF-α, IL-4, IL-10, IFN-γ, IL-17, and IL-2 levels in clinical samples (*n* = 50). (**H**–**N**) IL-6, TNF-α, IL-4, IL-10, IFN-γ, IL-17, and IL-2 content in cell function experiments (*n* = 3). Data (**A**–**G**) were analyzed using Mann–Whitney test. (**H**–**N**) Multi-group comparisons were processed via one way ANOVA. Results are presented as mean ± SEM. *: *p* < 0.05, **: *p* < 0.01, ***: *p* < 0.001.

IL-2, IL-4, IL-6, TNF-α, IFN-γ, IL-10, and IL-17 expression in plasma was measured using cytometric bead arrays. These findings revealed significant elevations in levels of IL-2, IL-4, IL-6, TNF-α, and IFN-γ in the BAVM group compared to the controls (*p* < 0.05, Figure 7A–E). No significant difference was observed in IL-10 and IL-17 levels (*p* > 0.05, Figure 7F,G). Subsequent functional experiments revealed that LPS treatment can induce the production of IL-6, TNF-α, IL-4, IL-10, IFN-γ, and IL-17 in the LPS group relative to controls (*p* < 0.05, Figure 7H–M). This effect can be partially reversed by NA (as seen with IL-6 and TNF-α in the LPS + NA group, *p* < 0.05, Figure 7H,I). IL-6 and TNF-α expression in the LPS + MTA 1 siRNA group was reduced compared to that of the LPS + control siRNA group (*p* < 0.01; Figure 7H,I). NA, 5-AZA, and *MTA1* siRNA treatments exhibited no significant effect on IL2, IL4, IL10, IL17, and IFN-γ.

## 4. Discussion

This study explored a possible relationship between *MTA1* promoter methylation and the mechanism of BAVM. The results revealed that: (1) *MTA1* methylation was lower in patients with BAVM than in the controls. (2) Methylation of CPG 8 and CPG 10 sites of *MTA1* exhibited a significant positive correlation with the Spetzler–Martin classification of BAVM and provided a greater predictive value for BAVM risk. (3) The MTA1 level in patients with BAVM was markedly higher than in the controls and was inversely proportional to the DNA methylation level. (4) Plasma ApoA concentrations in BAVM patients were significantly lower than those in the controls and correlated positively with *MTA1* methylation and negatively with *MTA1* expression level. (5) Cytokine (IL-2, IL-4, IL-6, TNF-α, and IFN-γ) concentrations in the plasma of BAVM patients were markedly increased relative to those in the controls. (6) Cell experiments demonstrated that LPS treatment strongly promoted cell migration and increased IL-6, TNF-α, IL-4, IL-10, IFN-α, and IL-17 concentrations. NA and *MTA1* silencing could effectively reverse the induction of IL-6 and TNF-α induced by LPS through the NF-κB pathway. (7) LPS and 5-AZA can reduce the degree of methylation of *MTA1*, and then increase the expression of MTA1. Also, NA can increase the degree of methylation of *MTA1* and decrease the expression of MTA1.

An increasing number of studies are reporting that DNA methylation is linked to the risk of stroke. This insight may help to predict the occurrence of stroke and serve as a target for treatment evaluation [30]. A previous study [10] showed that abnormal DNA methylation of vascular differentiation genes may be an important factor in BAVM lesions. It has also been observed that the hypermethylation of cyclin-dependent kinase inhibitor 2A (CDKN2A) can increase the risk of BAVM and has potential value for early diagnosis [31]. Our study revealed that *MTA1* methylation was decreased in BAVM patients relative to the controls, and was significantly linked with the BAVM clinical grade, suggesting its value as a risk predictor. In a study of malformations of cortical development (MCDS), researchers observed that DNA methylation can help establish a classification and diagnosis scheme for epilepsy-related MCDS [32]. DNA methylation in gene promoter regions participates in the development of cerebrovascular diseases by modulating gene expression via different signaling pathways [33,34]. Zhu et al. [35] reported that DNA methylation of the PTEN promoter may promote the occurrence of cavernous vascular malformation by regulating its gene expression through the ERK pathway. Our results suggest *MTA1* promoter methylation negatively regulates gene expression and participates in the pathology of BAVM.

Previous studies demonstrated that changes in the concentrations of different lipids in the blood are closely related to DNA methylation levels [36]. Cash et al. revealed that a reduction in DNA methylation in the peripheral blood of cardiovascular patients might closely relate to the increased blood LDL concentration and reduced HDL in patients [37]. ApoA is a protein component of HDL and has an important protective function against vascular lesions [38]. A HDL/ApoA-I complex may participate in pathological processes of microvascular and macrovascular diseases by regulating the self-association of von Willebrand factor (VWF) under shear stress [39]. The concentration of ApoA in the bloodstream has been found to be related to its DNA methylation. Thus, it could potentially be monitored as a risk biomarker to predict survival in patients with hepatocellular carcinoma and reduce the risk of this disease [40]. This study revealed that the ApoA concentration in the BAVM group is markedly reduced relative to that in the controls, which could be caused if the low concentration of ApoA in the patient is not enough to protect the health of its blood vessels, which would lead to pathological progression of BAVM. Our data also demonstrate that ApoA and HDL concentrations correlate positively with the degree of *MTA1* methylation and negatively with *MTA1* gene expression. However, the shortcoming is that this study did not further verify the relationship between ApoA, HDL, and methylation at the cellular experimental level.

Accumulating evidence indicates that an inflammatory response is involved in the pathogenesis of BAVM. Abnormally elevated concentrations of inflammatory cells have been confirmed in tissue specimens of human arteriovenous malformations [3,4,5]. Further studies have revealed that the accumulation of neutrophils in BAVM tissue is significantly associated with BAVM progression and rupture [4]. Researchers have also validated that the interaction between inflammatory cells and blood vessels is the key factor leading to BAVM using single-cell sequencing [41]. Our clinical data also showed that IL-2, IL-4, IL-6, TNF-α, and IFN-γ were significantly elevated in the plasma of BAVM patients. Subsequently, we used LPS to induce inflammation in cerebral vascular endothelial cells. The levels of inflammatory factors in cells treated with LPS increased significantly, while niacin could reduce the levels of inflammatory factors IL-6 and TNF-α in cells by regulating the NF-κB pathway.

As there is currently no accepted cellular model for BAVM, and considering the association between BAVM and inflammation, we used LPS to simulate a more realistic pathological state [42,43]. Niacin, a drug commonly used to increase HDL levels [27], also increased APOA concentrations. Previous studies have reported that niacin effectively inhibited LPS-induced IL-6, TNF-α, and IL-1β concentrations in mice [28] and was able to inhibit cell migration [44]. Our study showed that niacin may reverse LPS-induced elevated inflammatory factors in brain vascular endothelial cells through increasing the *MTA1* gene methylation level, reducing MTA1 protein expression, and the NF-κB pathway. Although the demethylation drug 5-AZA can increase the *MTA1* level, it has no noticeable effect on inflammatory factors. This may be because 5-AZA is a non-specific demethylation drug and may not be involved in the regulation of intracellular inflammation.

**Figure 8:**
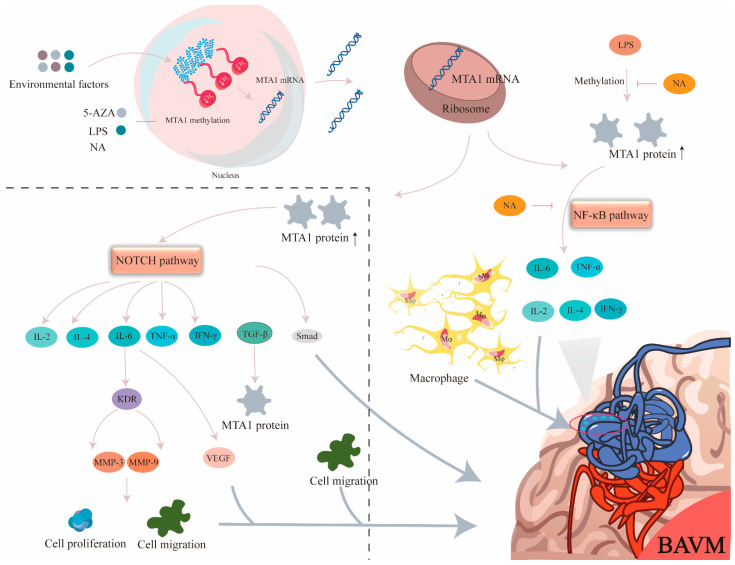
MTA1 methylation may mediate the mechanisms of BAVM occurrence. NA can impact MTA1 protein levels by regulating MTA1 promoter methylation, leading to decreasing of the expression of IL-6 and TNF-α by NF-κB pathway, and ultimately preventing abnormal angiogenesis. This effect can be reversed by NA. Our experimental data also found that the IL-2, IL-4, IL-6, TNF-α, and IFN-γ in the plasma of BAVM patients was increased relative to that of the control group. Combining our data with the literature, we speculate that MTA1 may also regulate the generation of downstream inflammatory factors such as interleukins via the NOTCH pathways, which may also promote the occurrence of BAVM. (Mø: microglia; NA: nicotinic acid; MMP-3: matrix metalloproteinase-3; MMP-9: matrix metalloproteinase-9; KDR: kinase insert domain-containing receptor).

MTA1 is involved in several inflammatory responses. MTA1 participates in the control of inflammatory responses and cytokine-mediated prostaglandin E2 production [17]. Furthermore, MTA1 co-regulates the expression and function of transglutaminase 2 during inflammatory responses and is involved in NF-κB signaling in macrophages [45]. Studies have reported that *MTA1* overexpression enhances cancer cell migration. Knocking out *MTA1* can reverse this effect [46] and can effectively reduce the production of interleukin and TNF-α [47,48]. A high expression of MTA1 protein in human placental cells may promote trophoblast migration and neovascularization [49]. Previous research has indicated that MTA1 is linked with the Notch, NF-κB, and TGF-β signaling pathways [18], which may induce IL-2, IL-4, IL-6, IL-17, IL-10, and IFN-g mediated inflammatory response in various diseases [50]. Recent research has also discovered that the Notch, NF-κB, and TGF-β pathways all play important roles in BAVM [20]. To explore the regulatory relationships between MTA1, the NF-κB pathway and inflammatory factors in BAVM, we used siRNA to knock down the expression of the *MTA1* gene. The results demonstrated that *MTA1* regulated the levels of IL-6 and TNF-α via the NF-κB pathway and participated in the pathological process of BAVM [51] (Figure 8).

Our study had certain limitations. First, there was no statistical distinction in the expression of *MTA1* observed in the clinical blood samples, which may be explained by RNA degradation during sample storage. We did, however, demonstrate differences in MTA1 expression in the cell experiments. Second, we only explored the correlation between ApoA, HDL, and methylation from the perspective of clinical samples, and did not further verify the specific regulatory mechanism; this needs to be investigated in subsequent studies. Third, we have only briefly verified the role of the NF-κB pathway in the production of inflammatory cytokines, and more experiments are needed to fully explore the detailed regulatory mechanisms of NF-κB. Fourth, owing to the difficulty in obtaining BAVM tissue samples, our study did not detect changes in *MTA1* in the diseased vascular tissue, necessitating further future research.

## 5. Conclusions

Our study shows that NA may regulate MTA1 expression by affecting promoter DNA methylation, reduce the levels of inflammatory factors such as IL-6 and TNF-α through the NF-κB pathway, and alleviate the pathological development of BAVM.

## Figures and Tables

**Table 1:** The clinical characteristics for all participants. TG: triglycerides, TC: total cholesterol, HDL: high-density lipoprotein, LDL: low-density lipoprotein, ApoA: apolipoprotein A, ApoB: apolipoprotein B, Lpa: lipoprotein a, and ApoE: apolipoprotein E.

Character	BAVM (*n* = 74)	Control (*n* = 74)	*t*/*x*	*p*
Age (year)	49.70 ± 12.18	52.99 ± 8.35	1.913	0.058
Sex (male, *n*)	37	37	0.020	0.888
Smoking (*n*)	18	11	0.970	0.325
Drinking (*n*)	11	12	0.040	0.841
TG (mmol/L)	1.40 ± 0.79	1.50 ± 0.68	0.825	0.410
TC (mmol/L)	4.66 ± 0.96	4.74 ± 0.94	0.526	0.600
HDL (mmol/L)	1.15 ± 0.28	1.23 ± 0.27	1.852	0.066
LDL (mmol/L)	2.96 ± 0.64	3.03 ± 0.66	0.583	0.561
ApoA (g/L)	1.18 ± 0.24	1.31 ± 0.26	3.077	0.002
ApoB (g/L)	0.89 ± 0.22	0.92 ± 0.19	0.843	0.401
Lpa (mg/dL)	26.96 ± 26.05	19.59 ± 17.15	2.032	0.044
ApoE (g/L)	51.63 ± 16.54	46.33 ± 15.48	2.012	0.046

## Data Availability

The research created experimental data that can be found in the tables and figures presented in this manuscript.

## Supplementary Materials

The following supporting information can be downloaded at: https://www.mdpi.com/article/10.3390/biom13101495/s1, Figure S1: Correlation analysis between MTA1 methylation and clinical data; Table S1: Usage details of clinical samples in different experimental groups. Original western blot images can be found in Supplementary Materials.
