# Supplementary material for: Nicotinic Acid-Mediated Modulation of Metastasis-Associated Protein 1 Methylation and Inflammation in Brain Arteriovenous Malformation
Source: Biomolecules. 2023 Oct 8;13(10):1495. doi: 10.3390/biom13101495 (PMC10605296; doi:10.3390/biom13101495)
Supplement: Supplementary file 1 [file biomolecules-13-01495-s001.zip › biomolecules-2493321-supplementary.pdf]

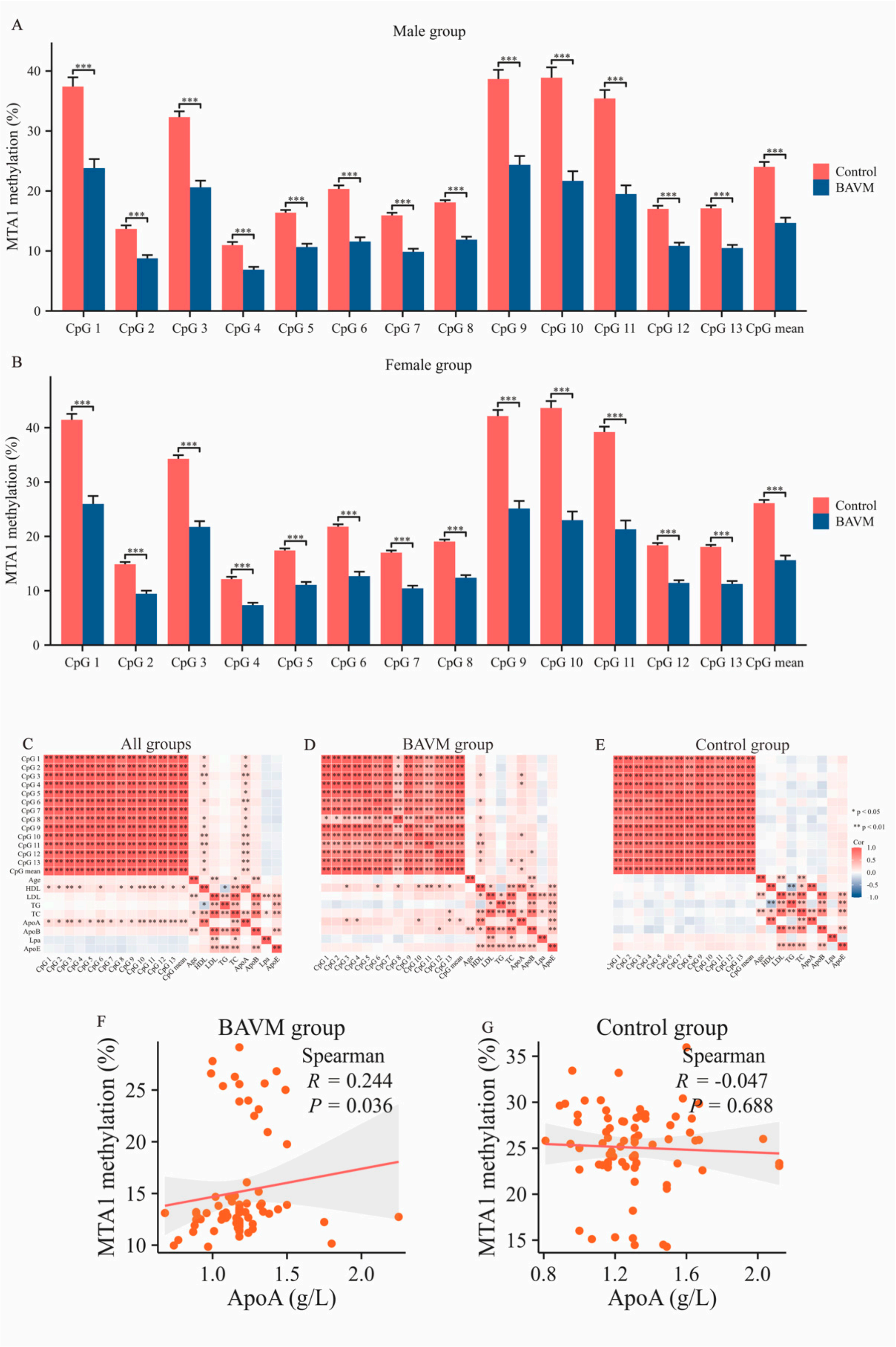

Supplementary Figure S1. Correlation analysis between MTA1 methylation and clinical data.

(A) Differences in methylation between BAVM and controls among males. (B) Differences in methylation between BAVM and controls among females. (C) Correlation analysis in all groups. (D) Correlation analysis in BAVM group. (E) Correlation analysis in Controls. (F) Correlation of MTA1 methylation with APOA in BAVM group. (G) Correlation of MTA1 methylation with APOA in control group. Wilcoxon rank-sum test and spearman correlation analysis were used to analyze data. Results are presented as mean ± SEM. *: $p < 0.05$, **: $p < 0.01$, ***: $p < 0.001$.

Supplementary Table S1. Usage details of clinical samples in different experimental groups.

Character	BAVM	Control	Analysis information
All volunteers	74	74	Collection of clinical data including age, sex, smoking, drinking, TG, TC, HDL, LDL, ApoA, ApoB, Lpa, ApoE.<br>Collection of peripheral white blood cells for the DNA methylation pyrosequencing.
CTA	23	N/A	Collection of computed tomography angiography for analysis.
RT-qPCR	18	18	Collection of peripheral white blood cells for *MTA1* mRNA expression.
ELISA	32	32	Collection of peripheral white blood cells for MTA1 protein expression.
Cytometric bead array	50	50	Collection of plasma for inflammatory factor analysis.

RT-qPCR: Quantitative real-time polymerase chain reactions, CTA: Computed tomography angiography, ELISA: Enzyme-linked immunosorbent assay, TG: Triglycerides, TC: Total cholesterol, HDL: High-density lipoprotein, LDL: Low-density lip-oprotein, ApoA: Apolipoprotein A, ApoB: Apolipoprotein B, Lpa: Lipoprotein a, ApoE: Apolipoprotein E, MTA1: Metastasis-associated protein 1.
