# Supplementary material for: Nicotinic Acid-Mediated Modulation of Metastasis-Associated Protein 1 Methylation and Inflammation in Brain Arteriovenous Malformation
Source: Biomolecules. 2023 Oct 8;13(10):1495. doi: 10.3390/biom13101495 (PMC10605296; doi:10.3390/biom13101495)

MTA1

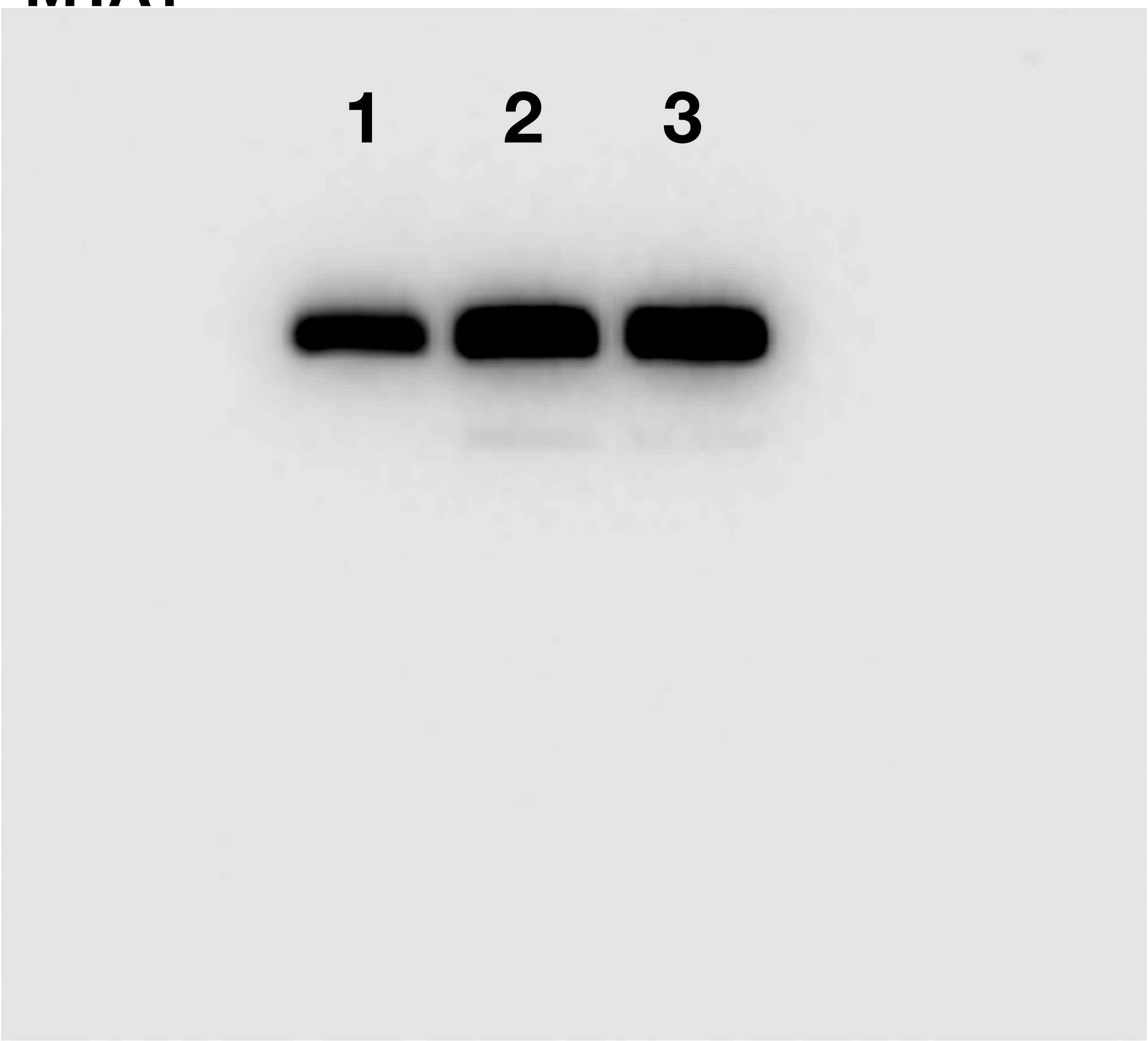

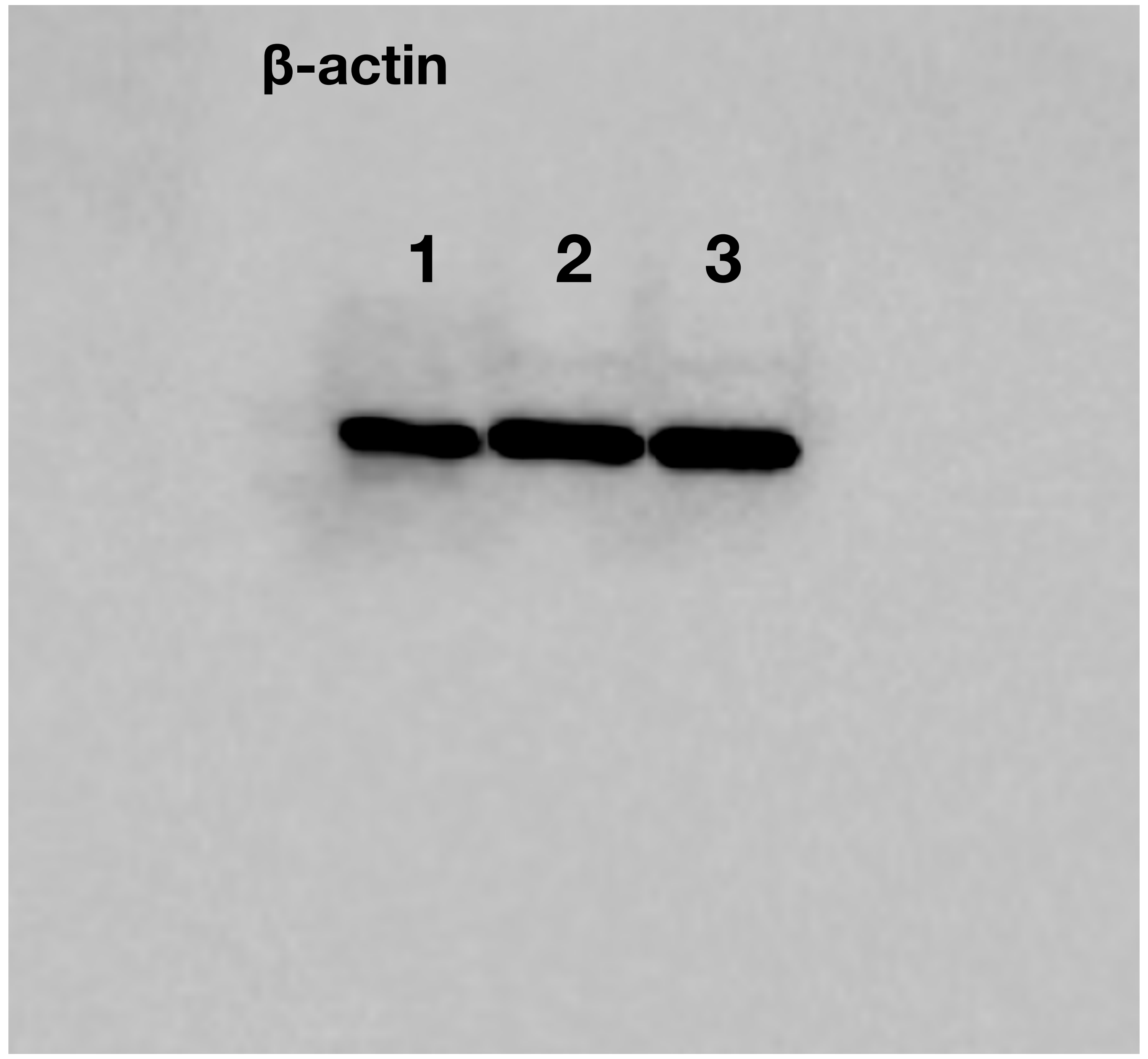

Western blot original bands from Figure 5F, Group 1 bands used for analysis

1 Control
2 2μM
3 4μM

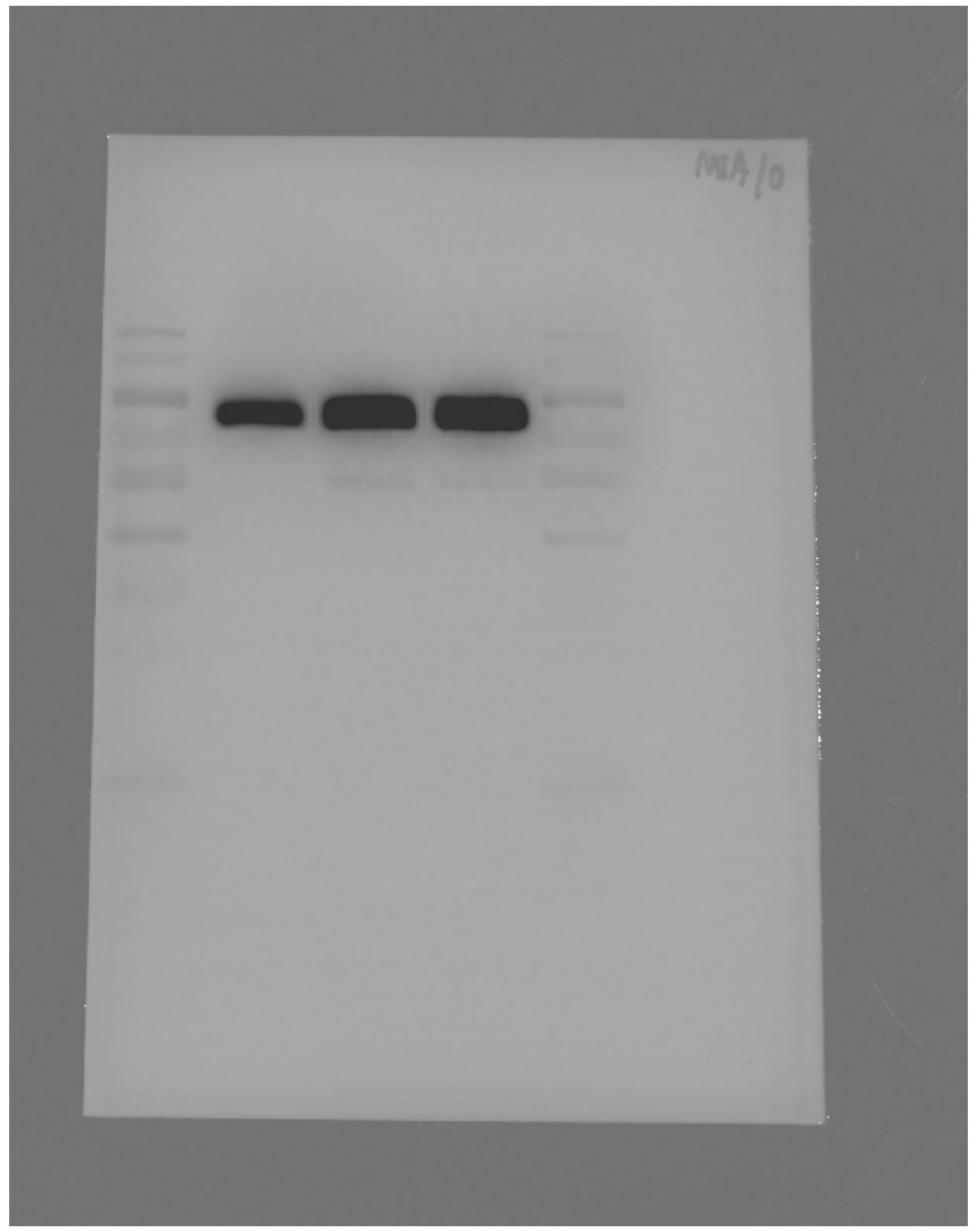

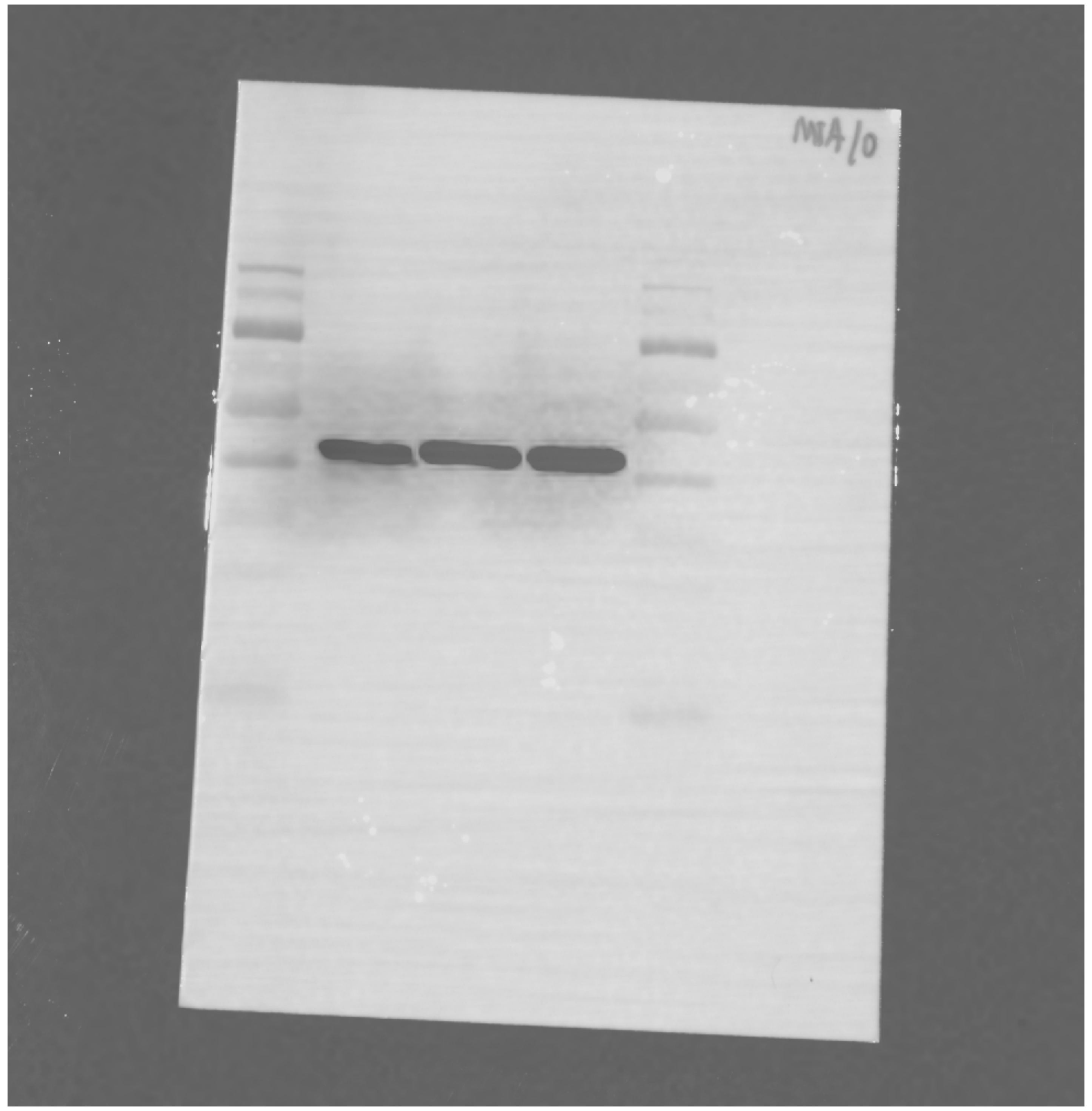

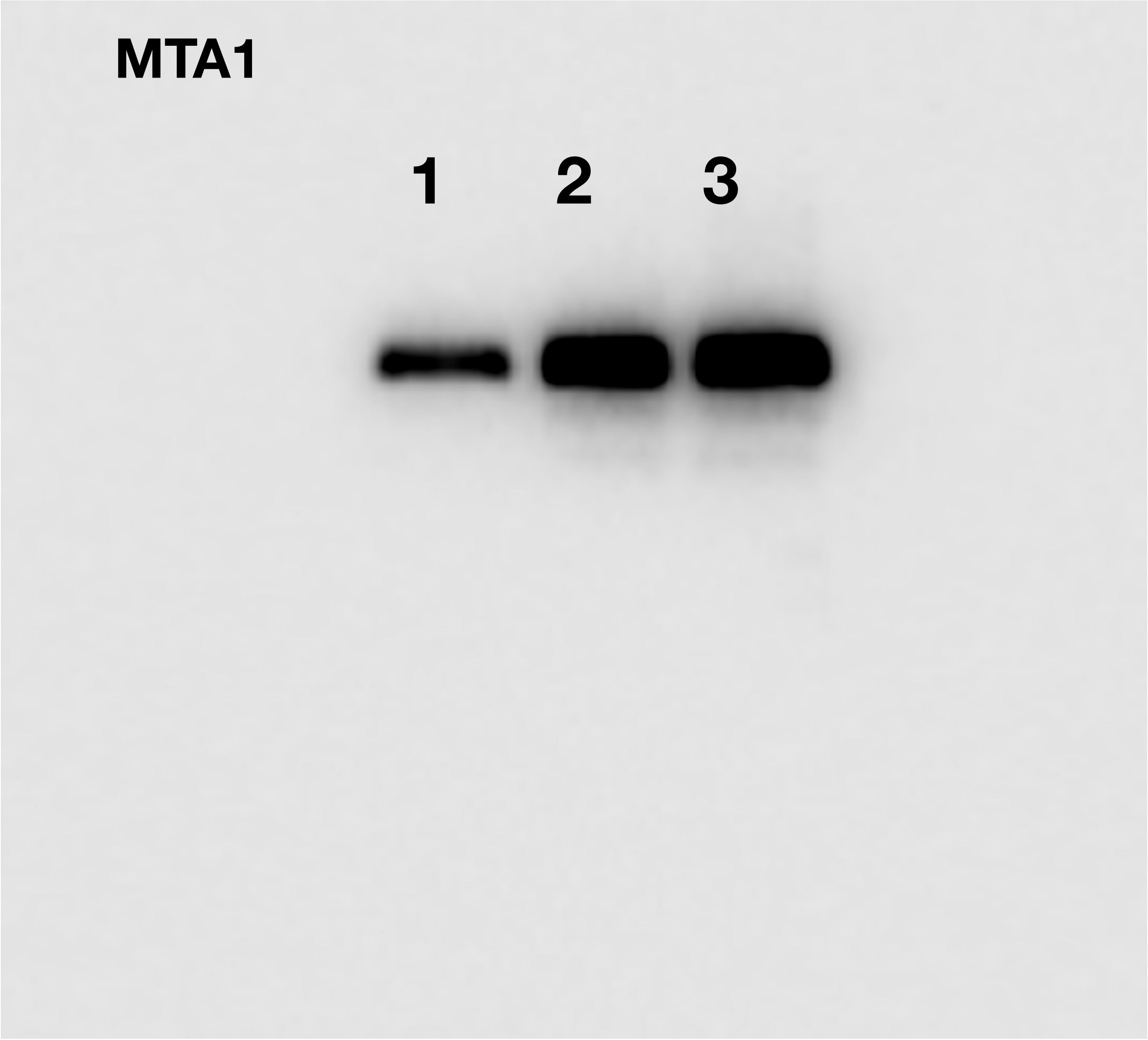

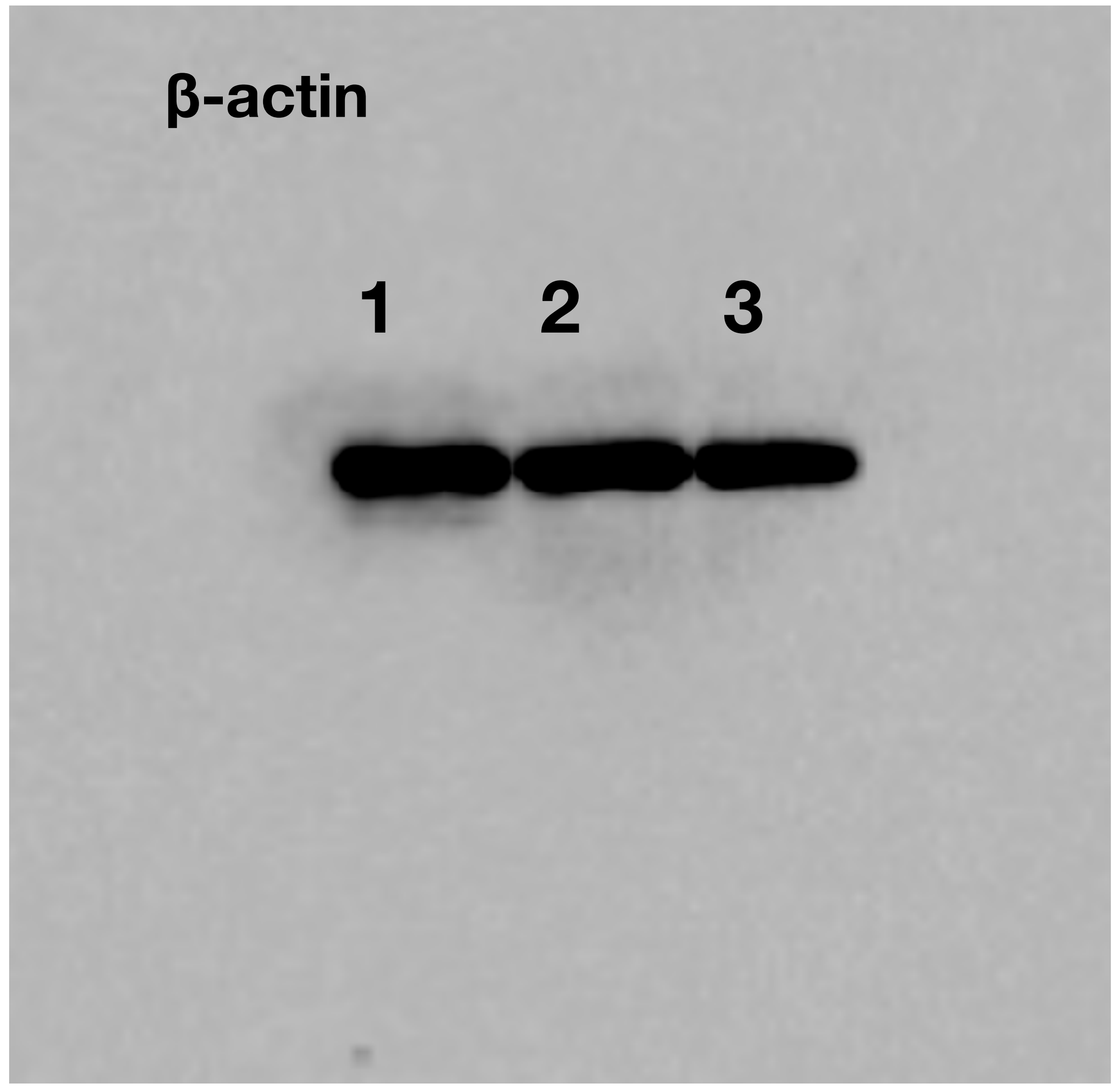

Western blot original bands from Figure 5F, Group 2 bands used for analysis

1 Control
2 2μM
3 4μM

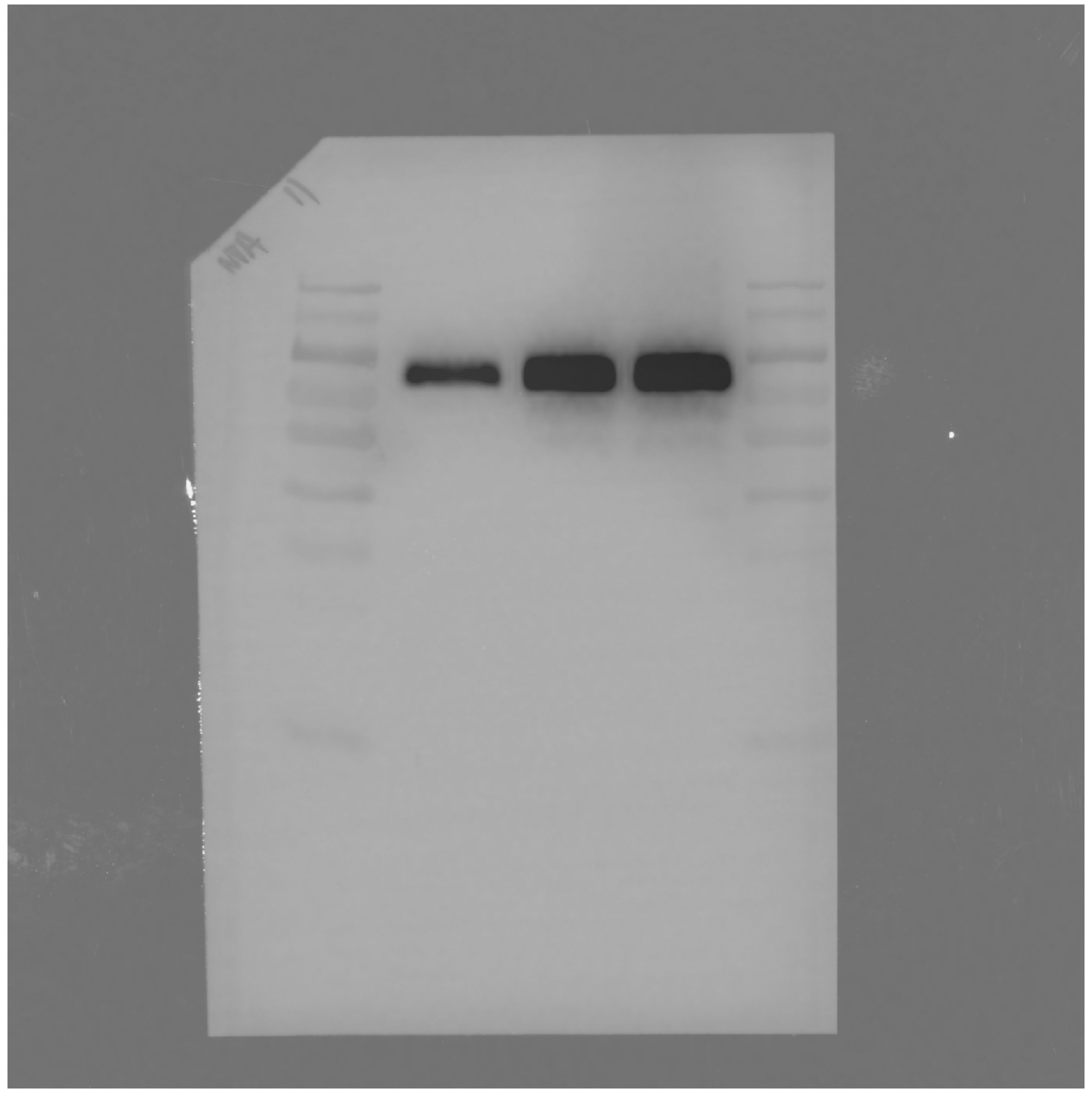

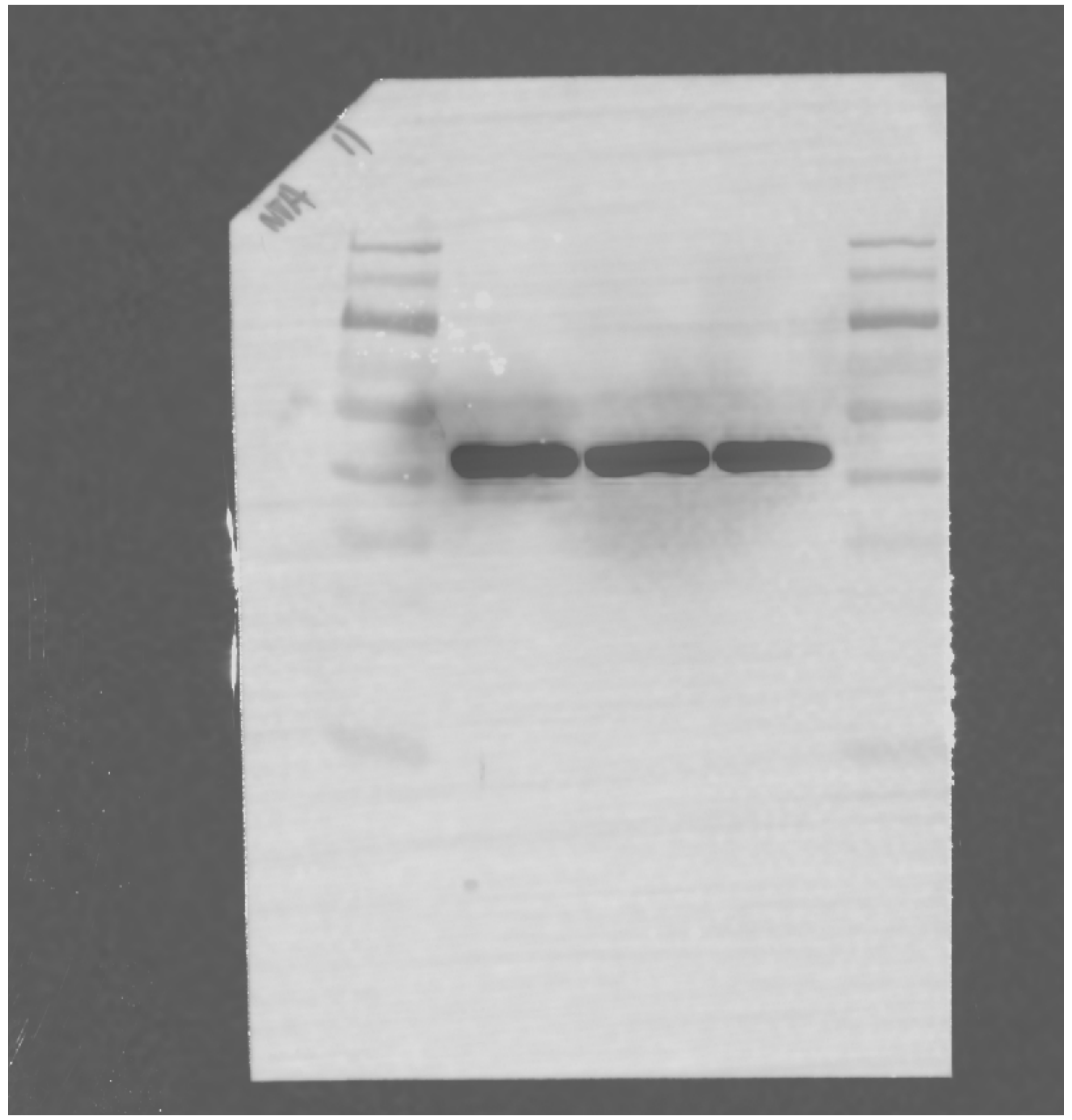

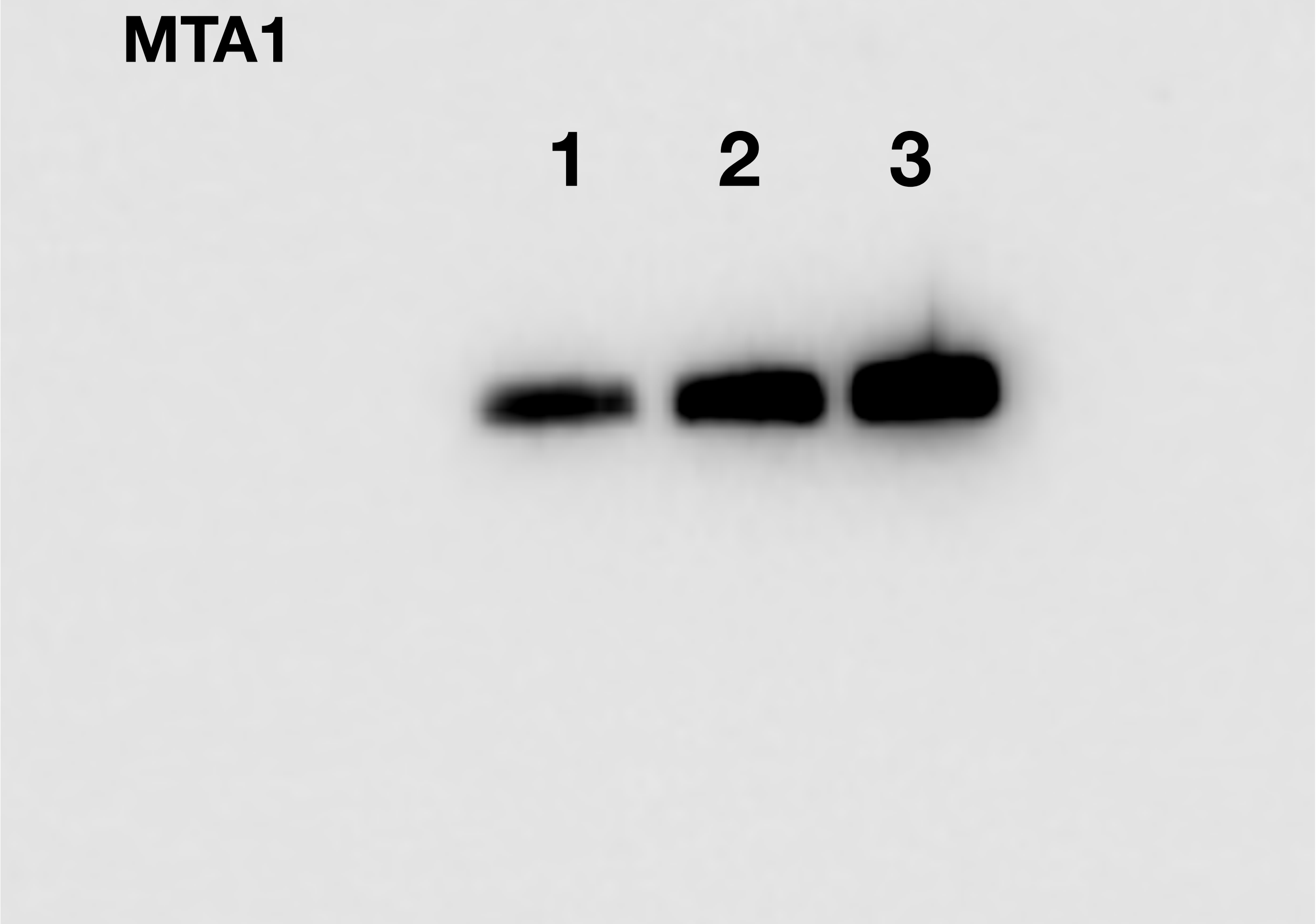

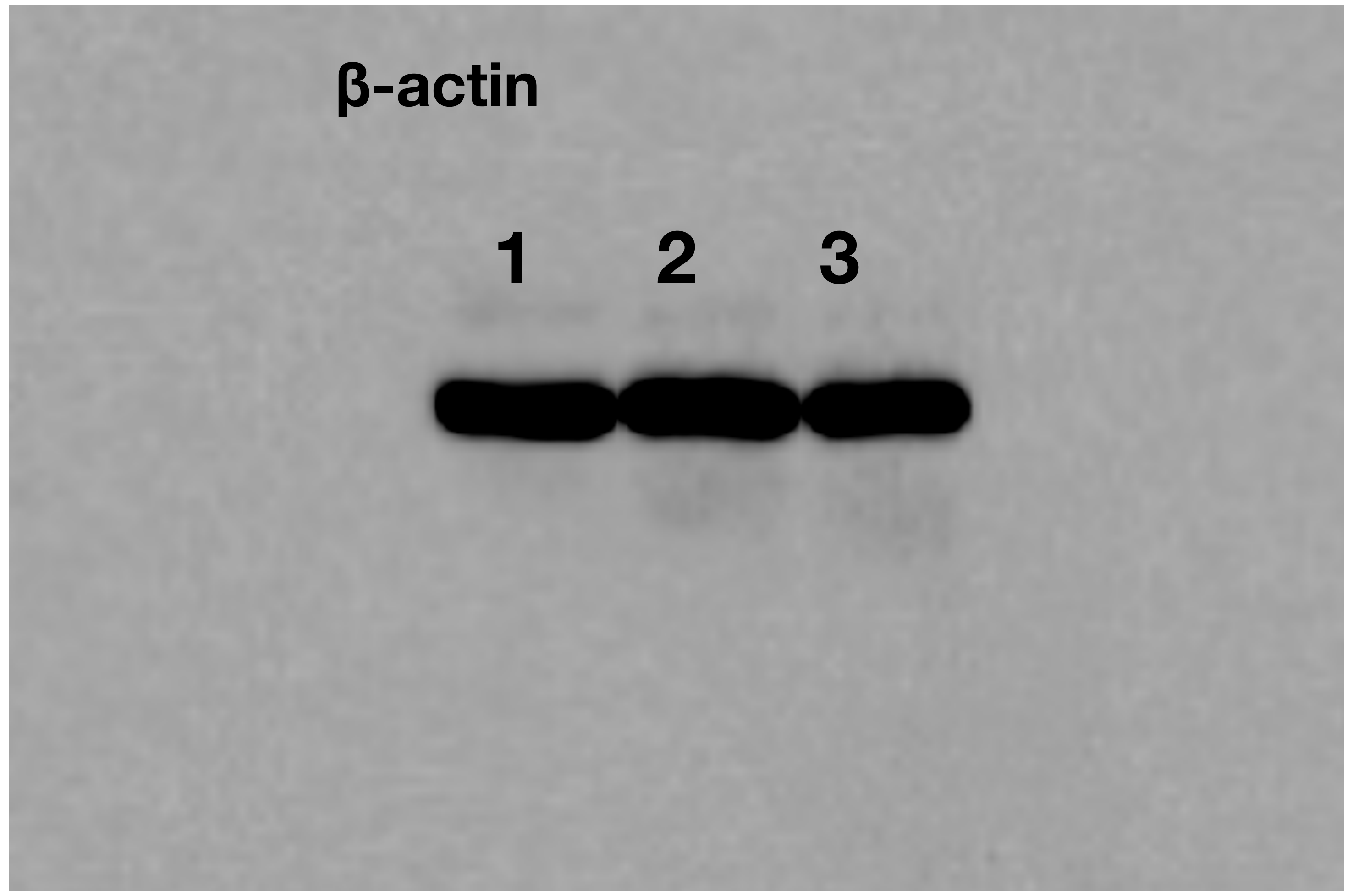

Western blot original bands from Figure 5F, Group 3 bands used for analysis

1 Control
2 2μM
3 4μM

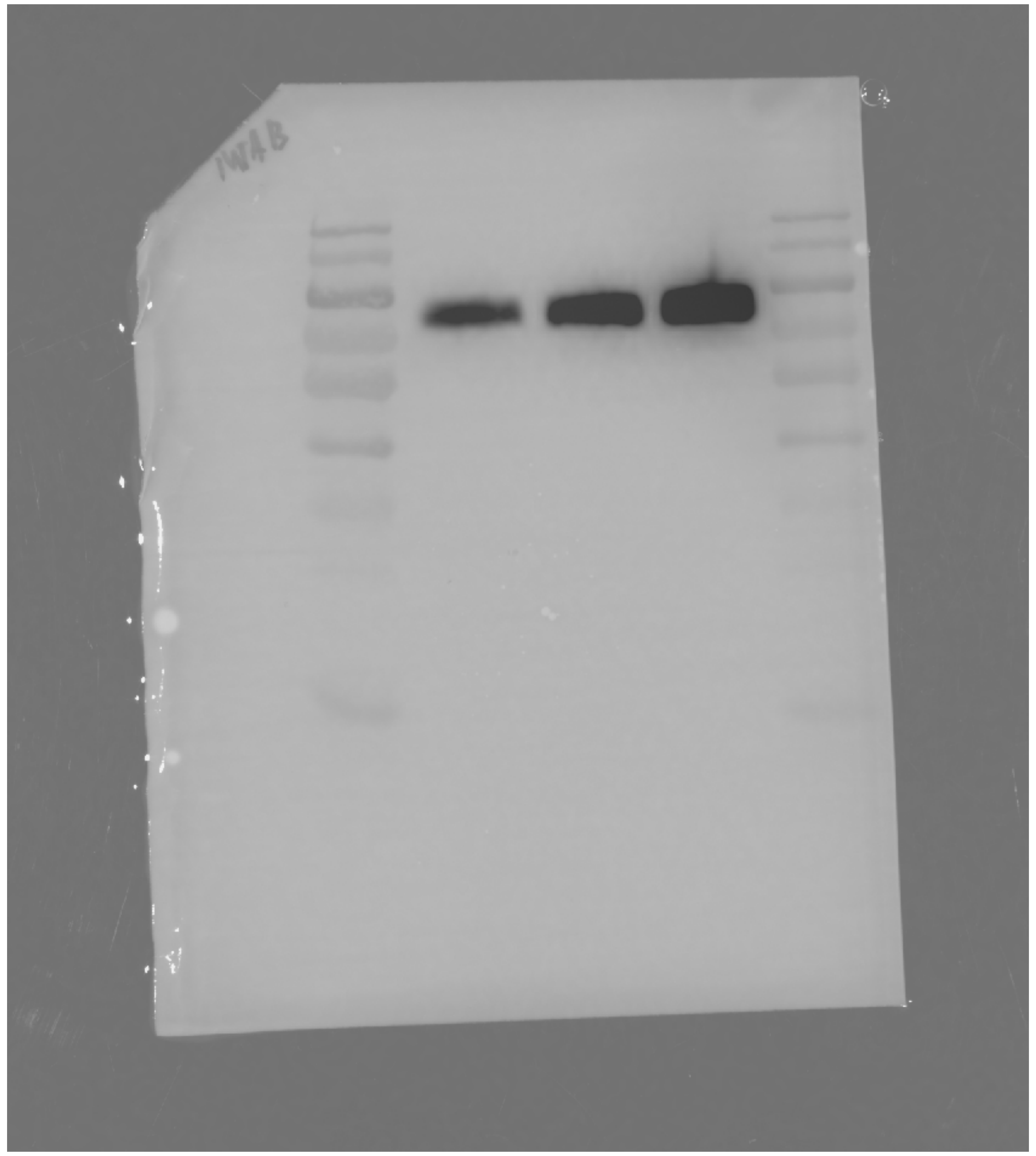

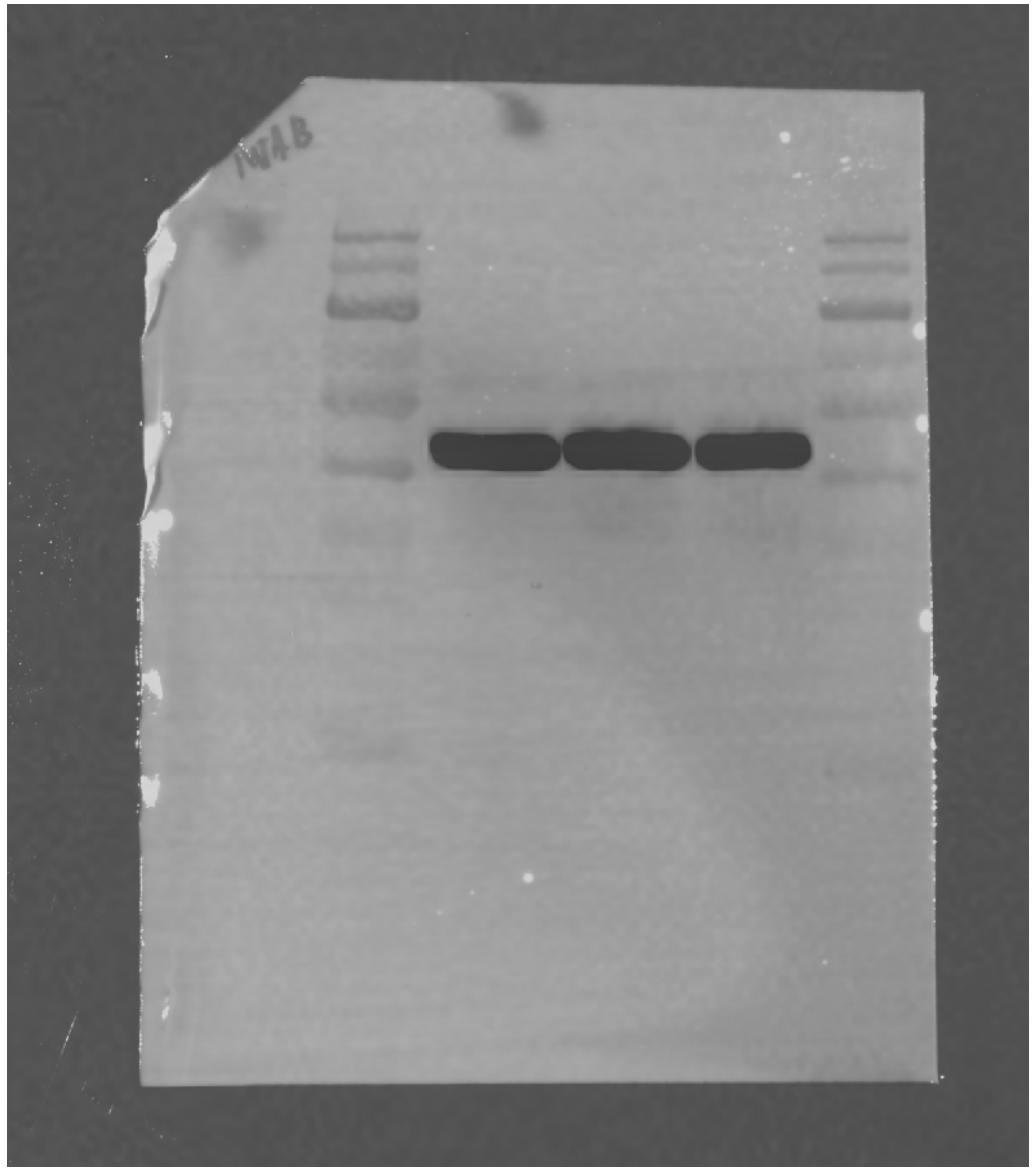

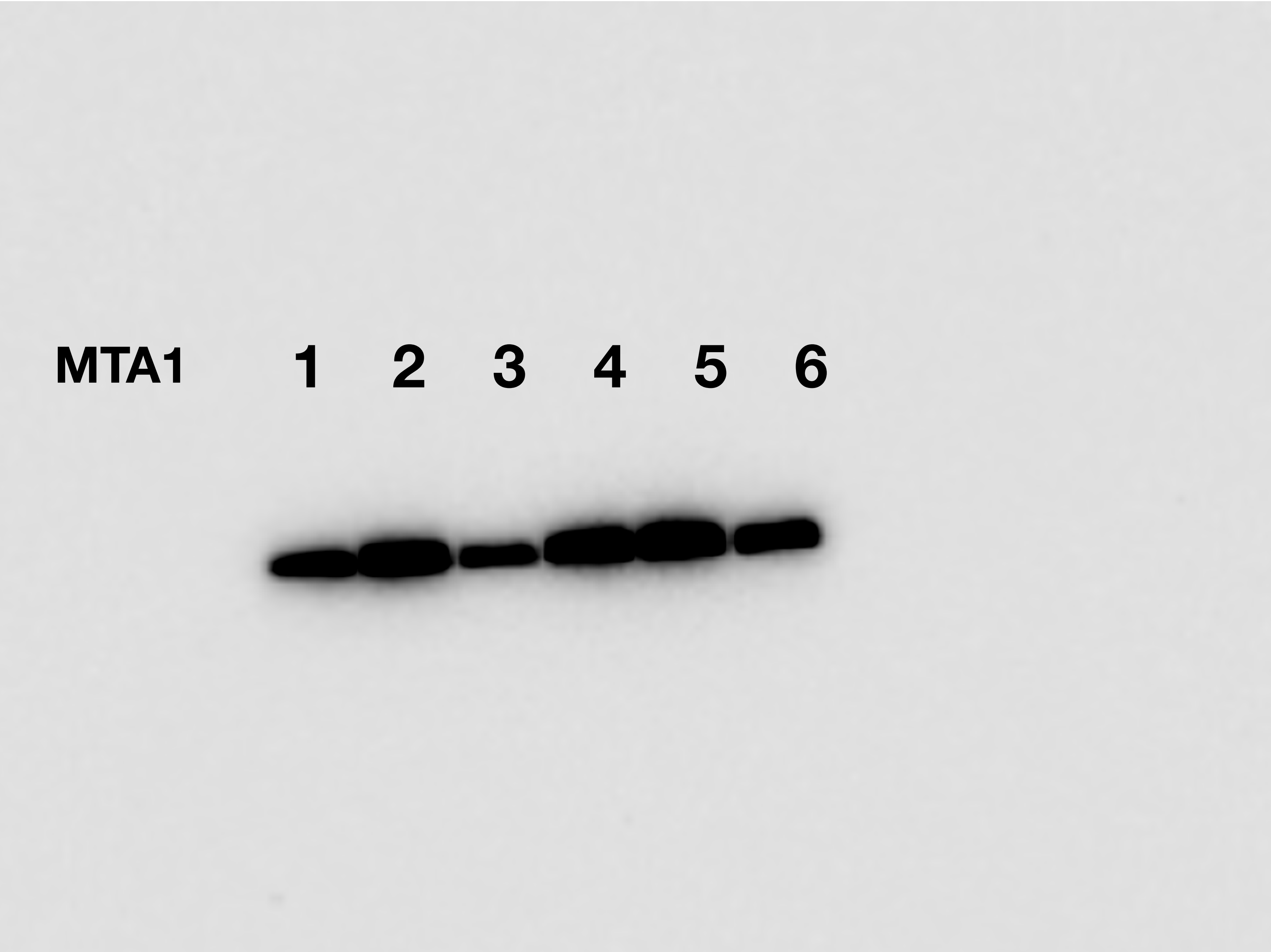

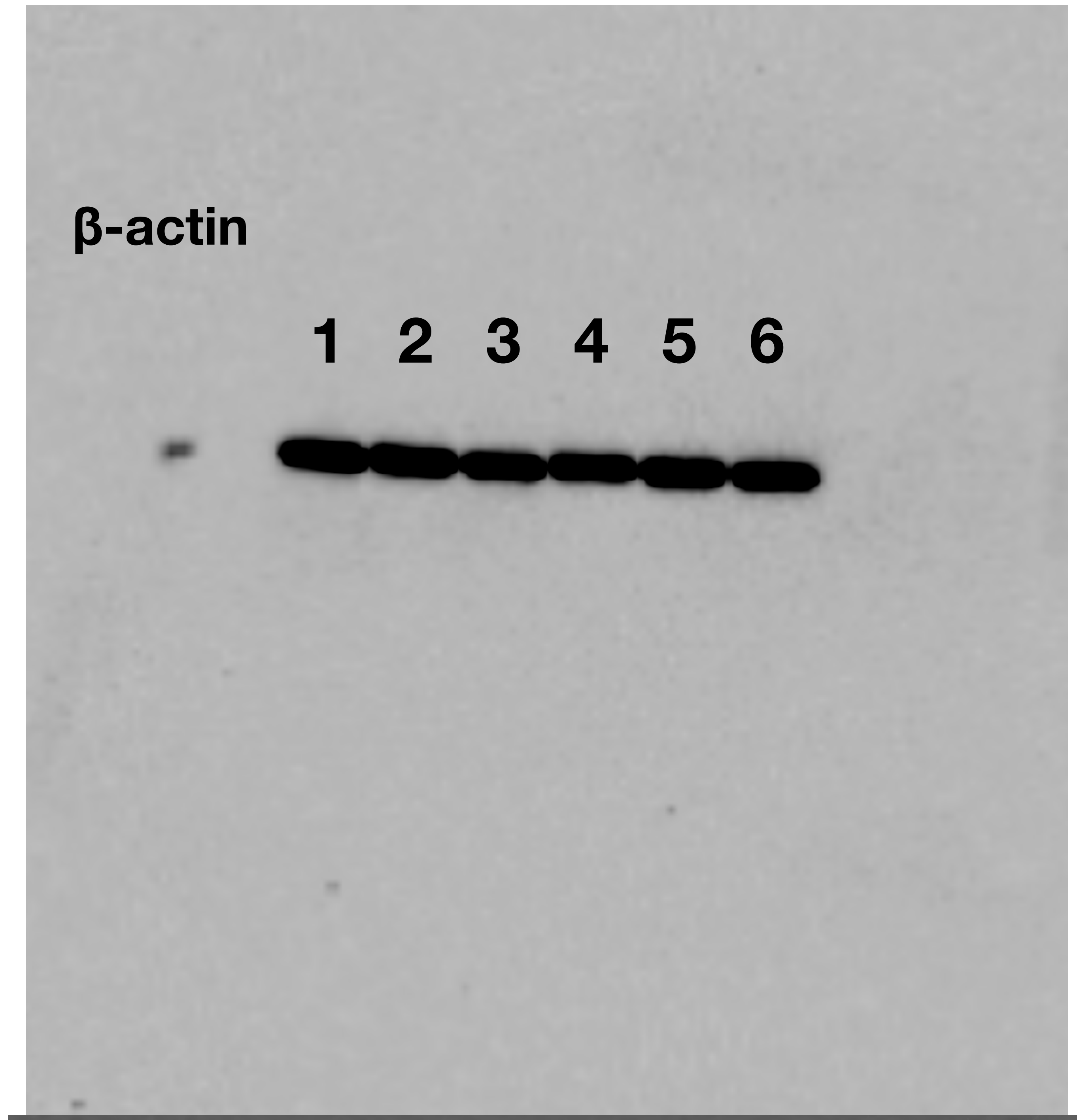

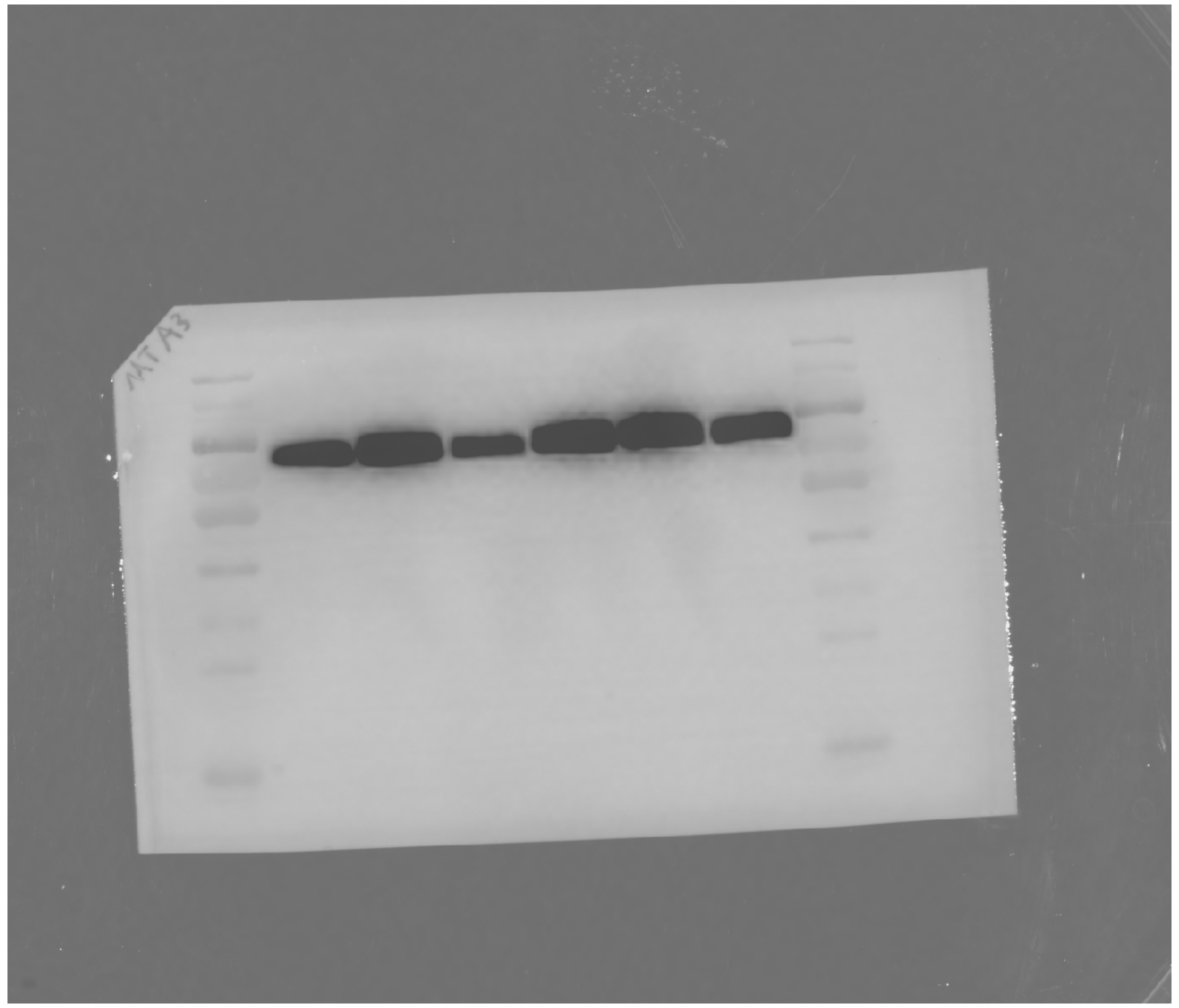

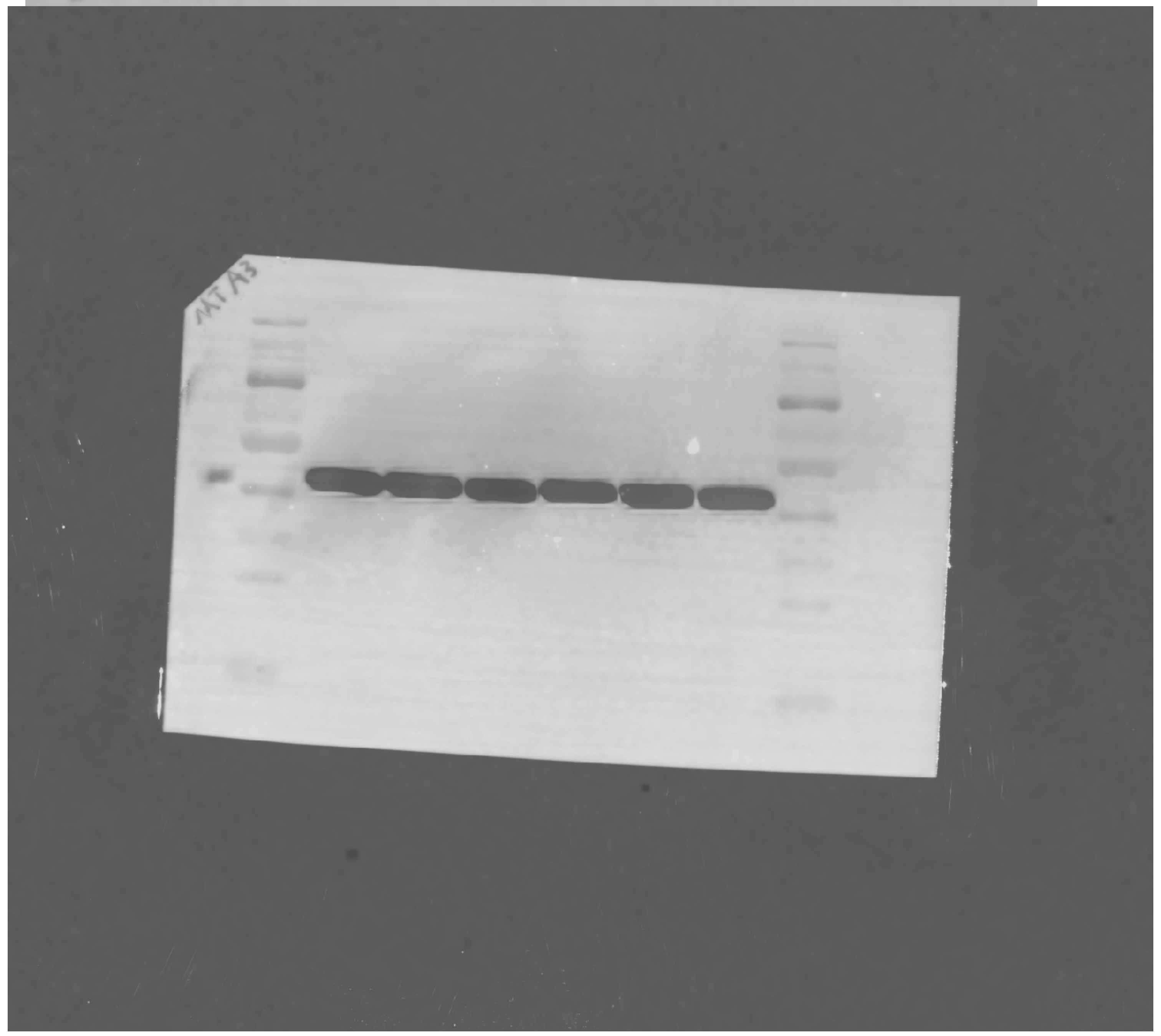

Western blot original bands from Figure 5H, Group 1 bands used for analysis

1 Control
2 LPS
3 LPS+NA
4 LPS+5-AZA
5 LPS+control siRNA
6 LPS+MTA1 siRNA

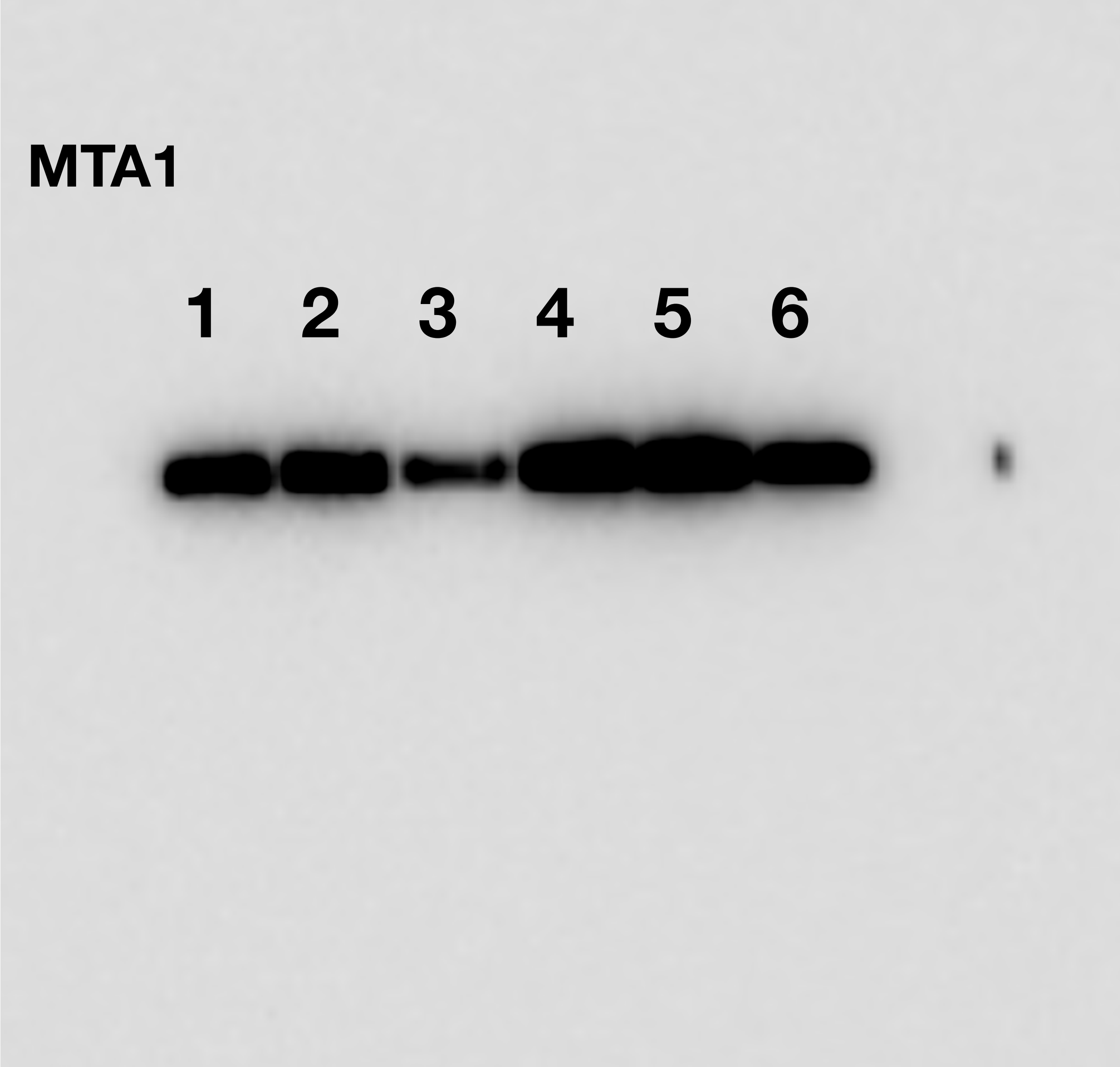

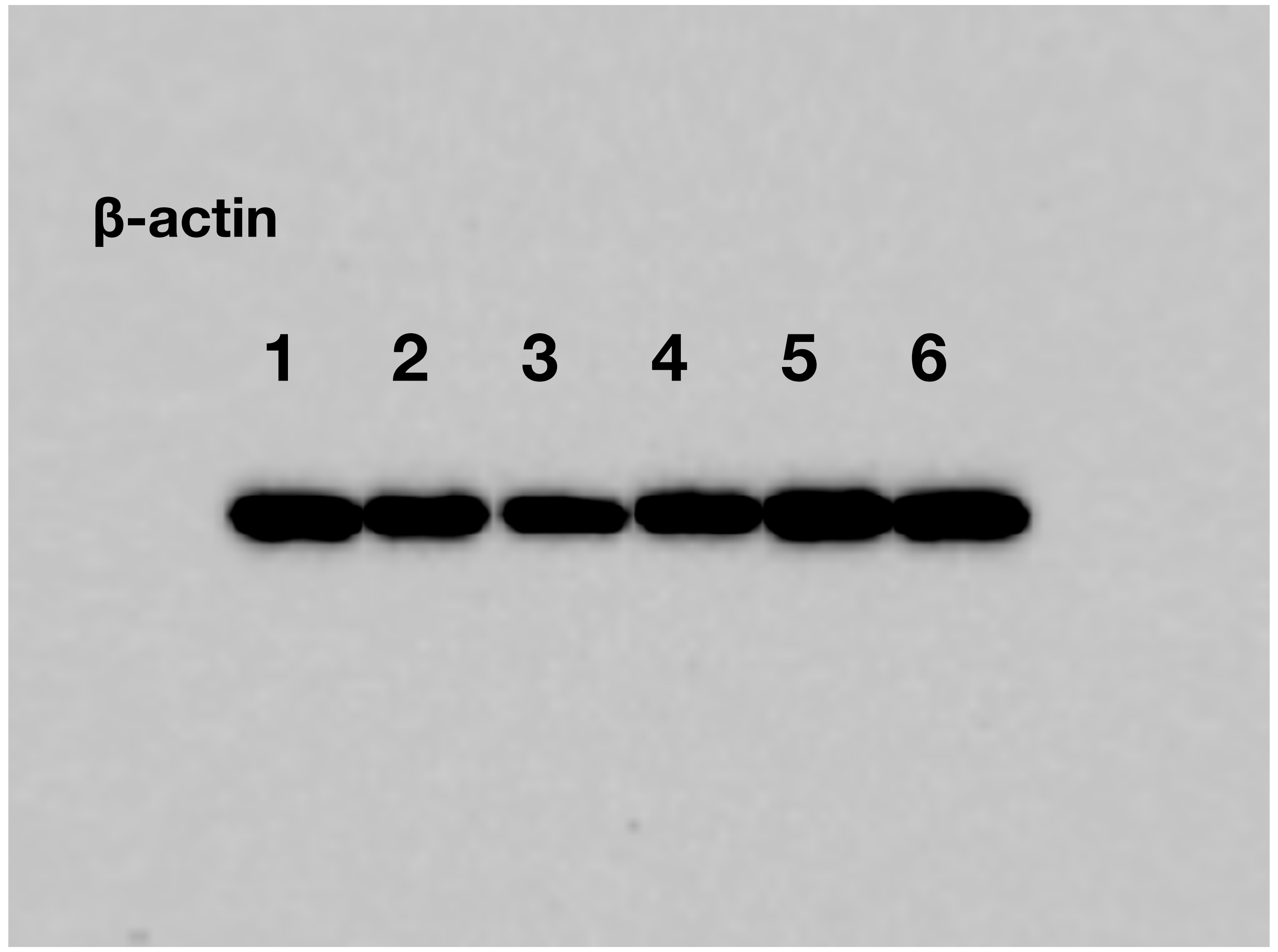

Western blot original bands from Figure 5H, Group 2 bands used for analysis

1 Control
2 LPS
3 LPS+NA
4 LPS+5-AZA
5 LPS+control siRNA
6 LPS+MTA1 siRNA

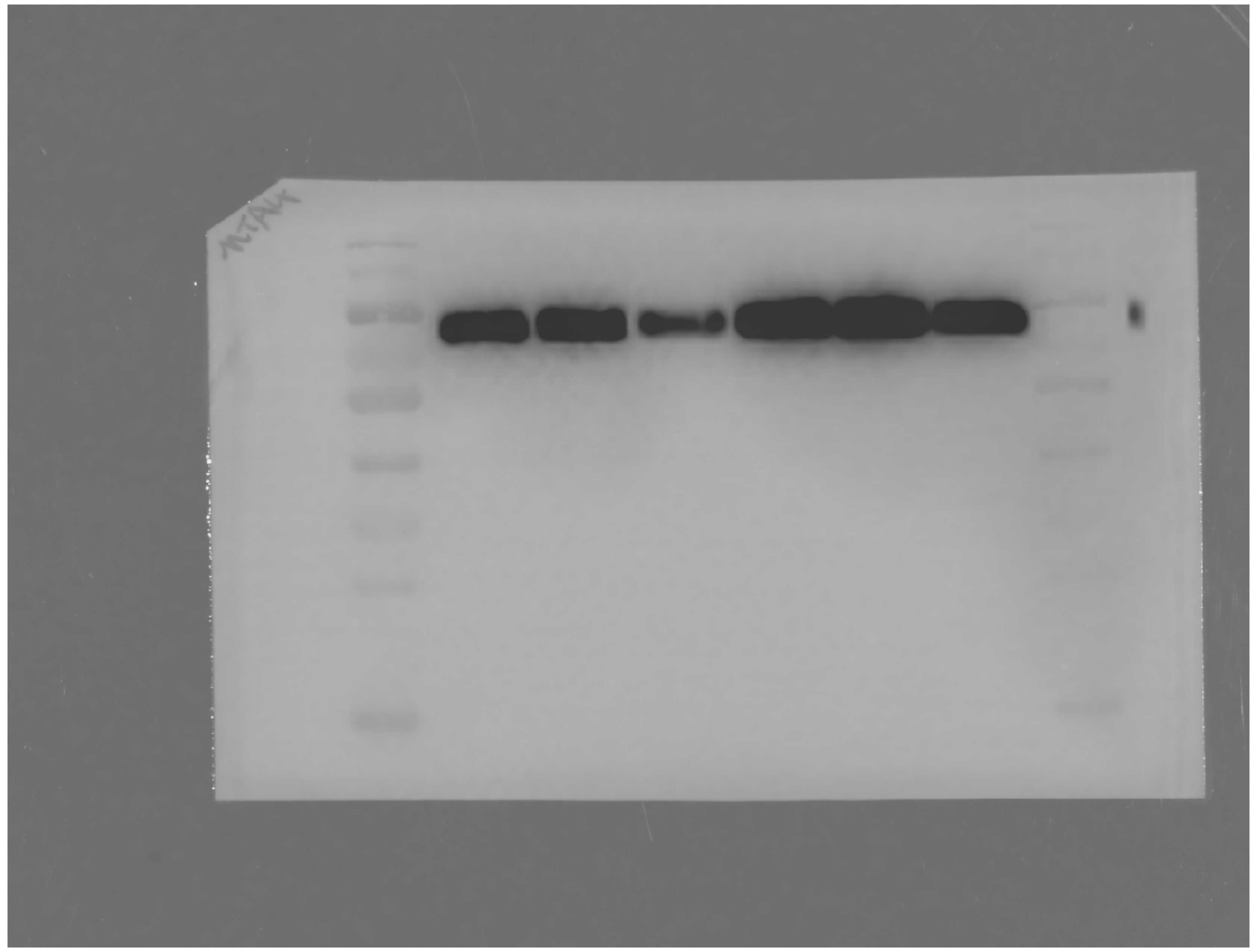

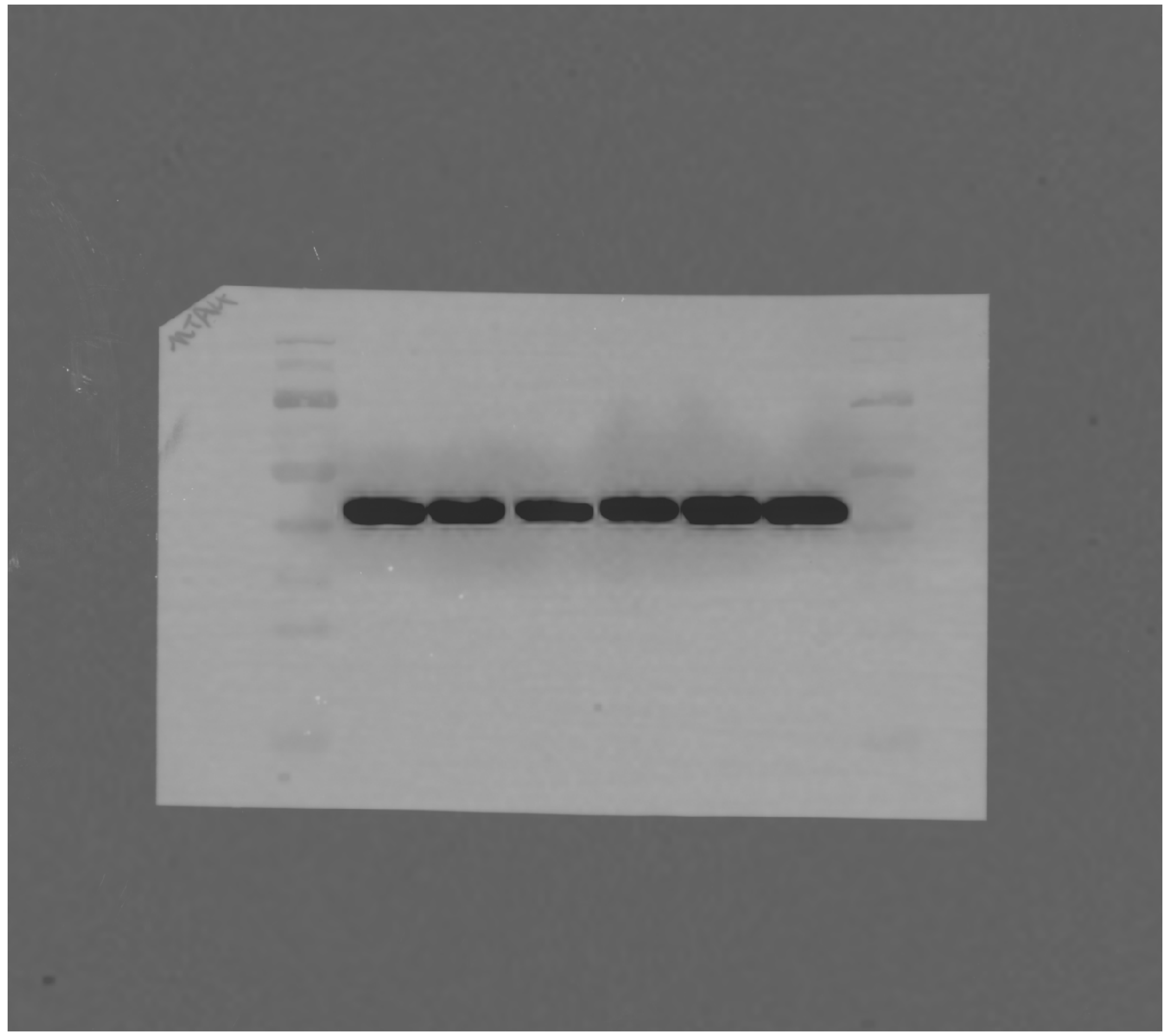

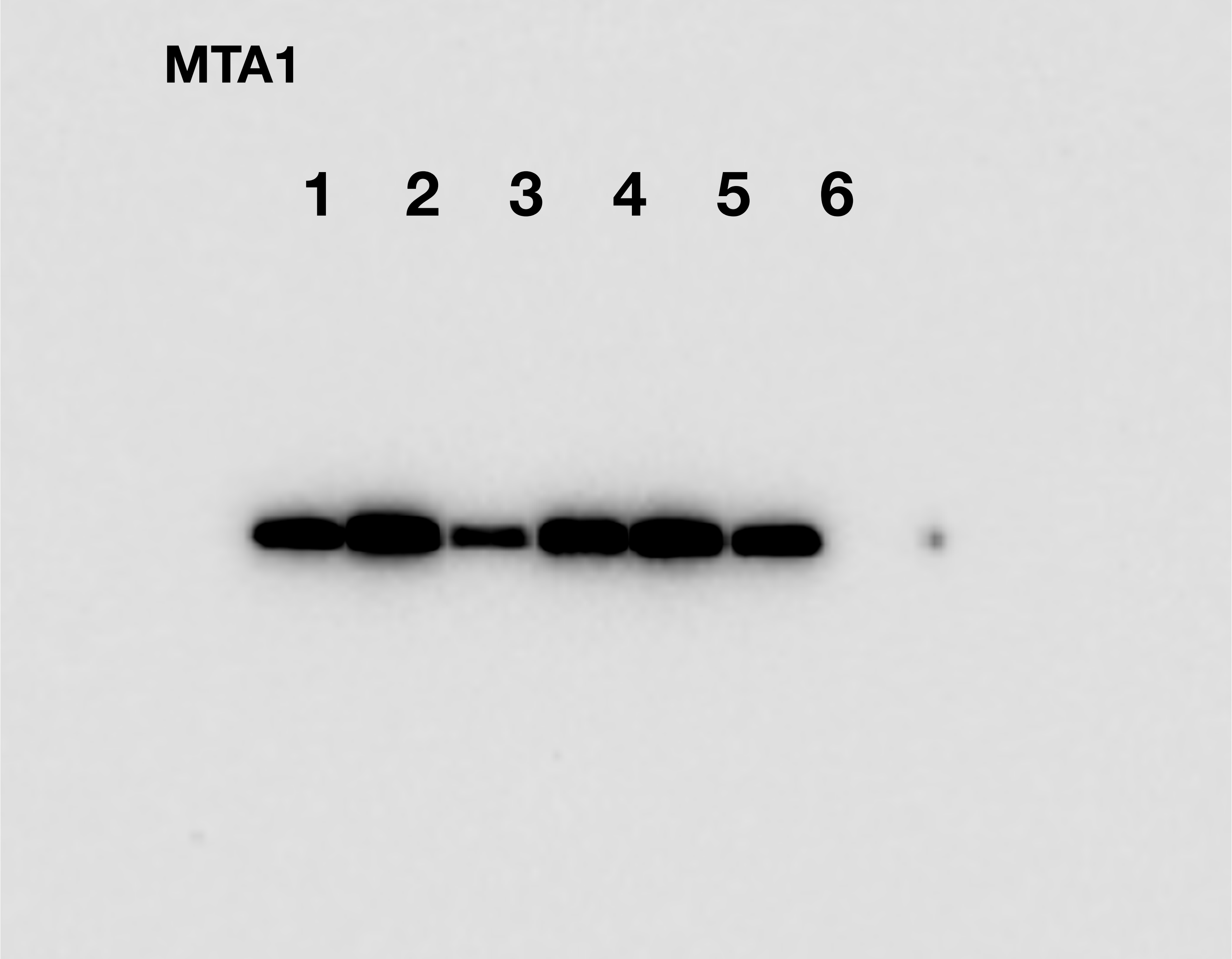

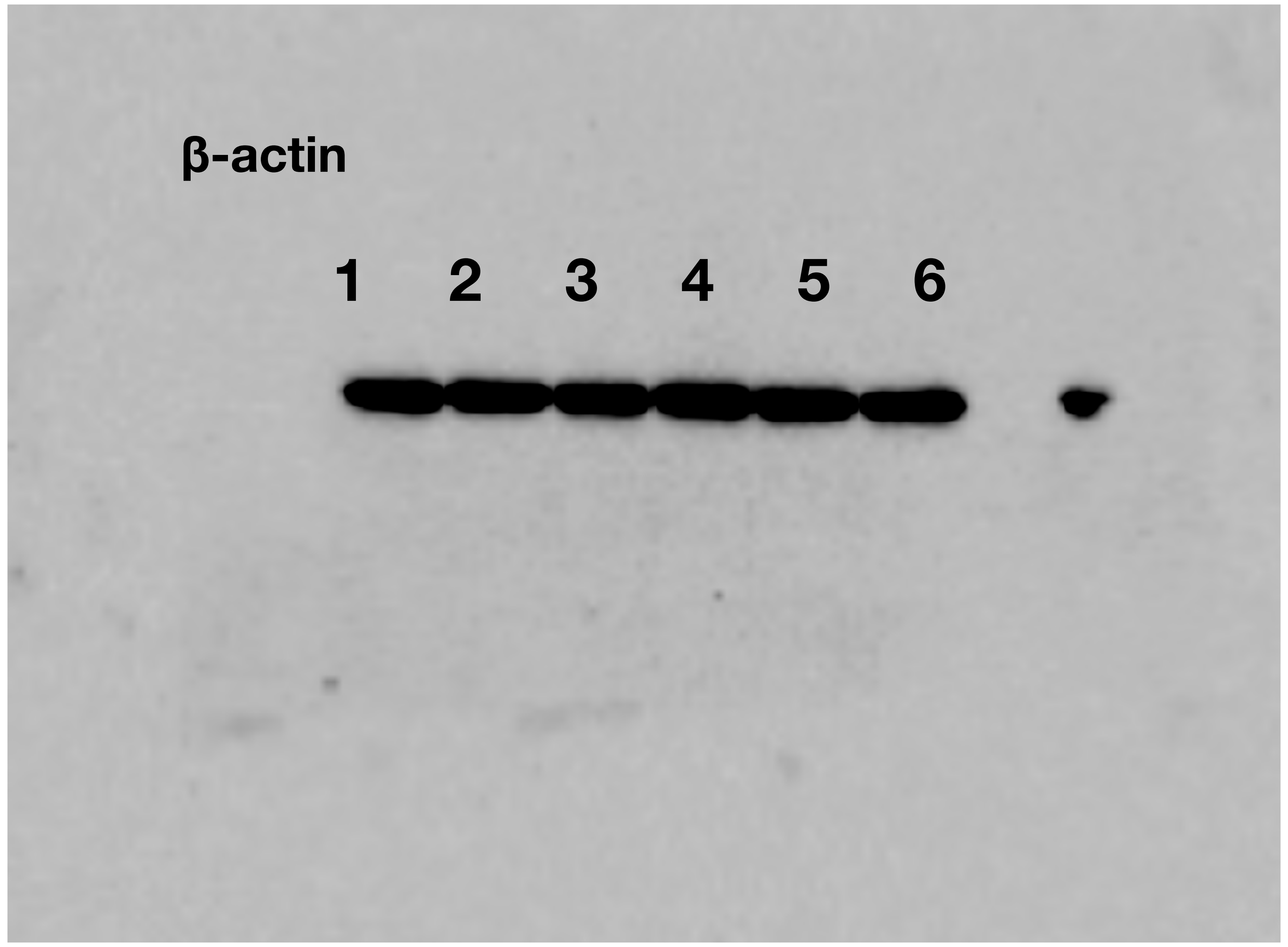

Western blot original bands from Figure 5H, Group 3 bands used for analysis

1 Control
2 LPS
3 LPS+NA
4 LPS+5-AZA
5 LPS+control siRNA
6 LPS+MTA1 siRNA

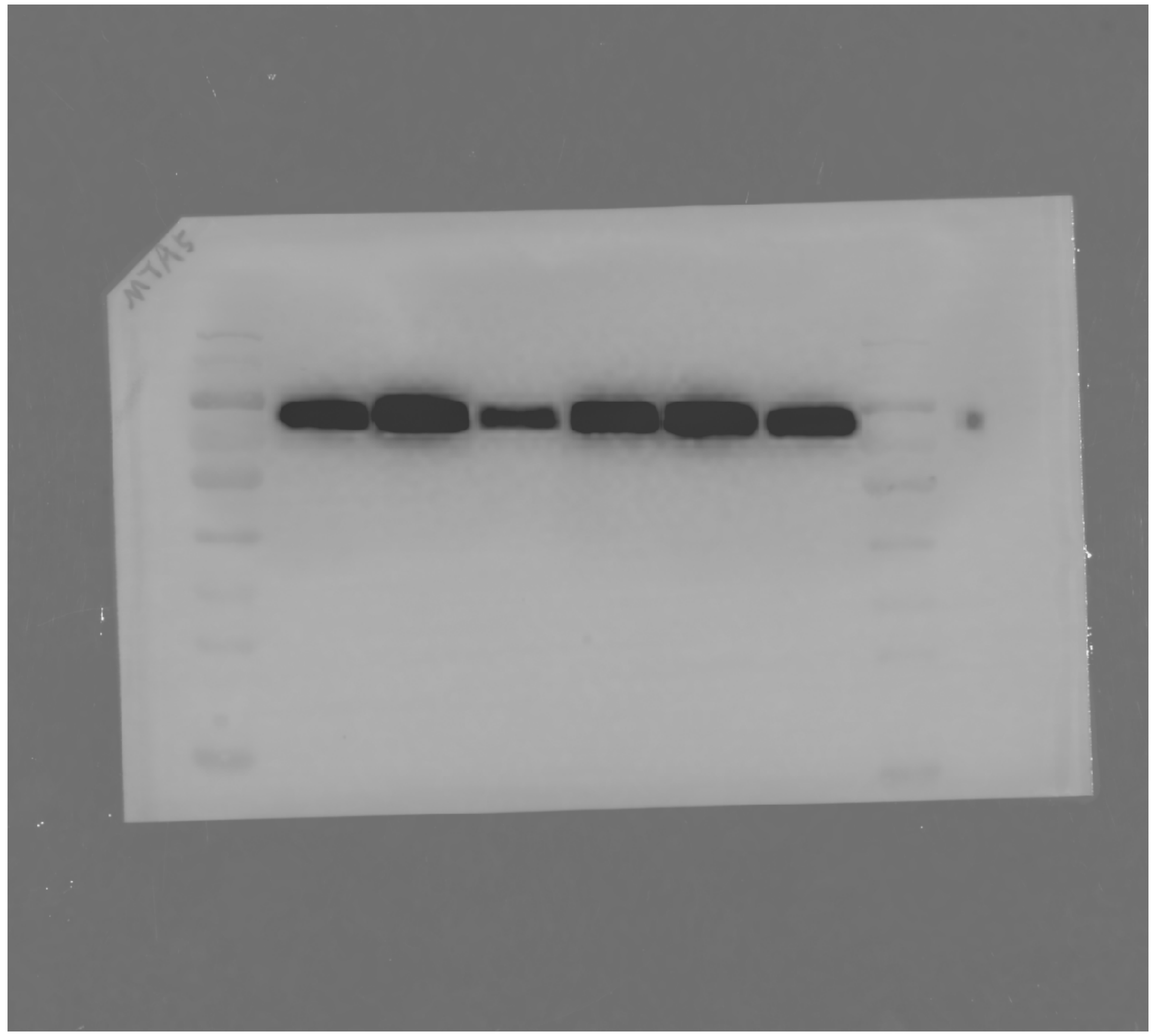

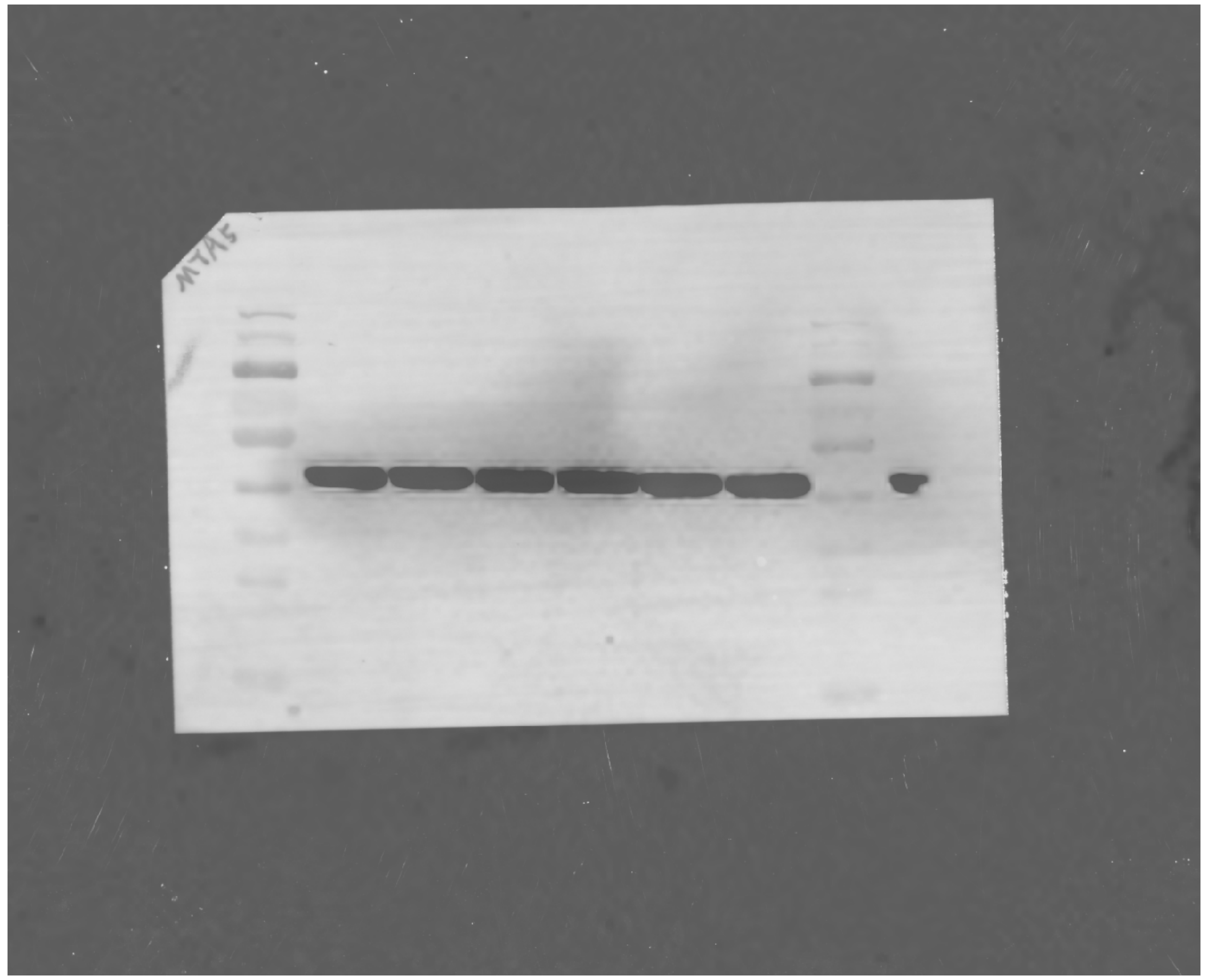

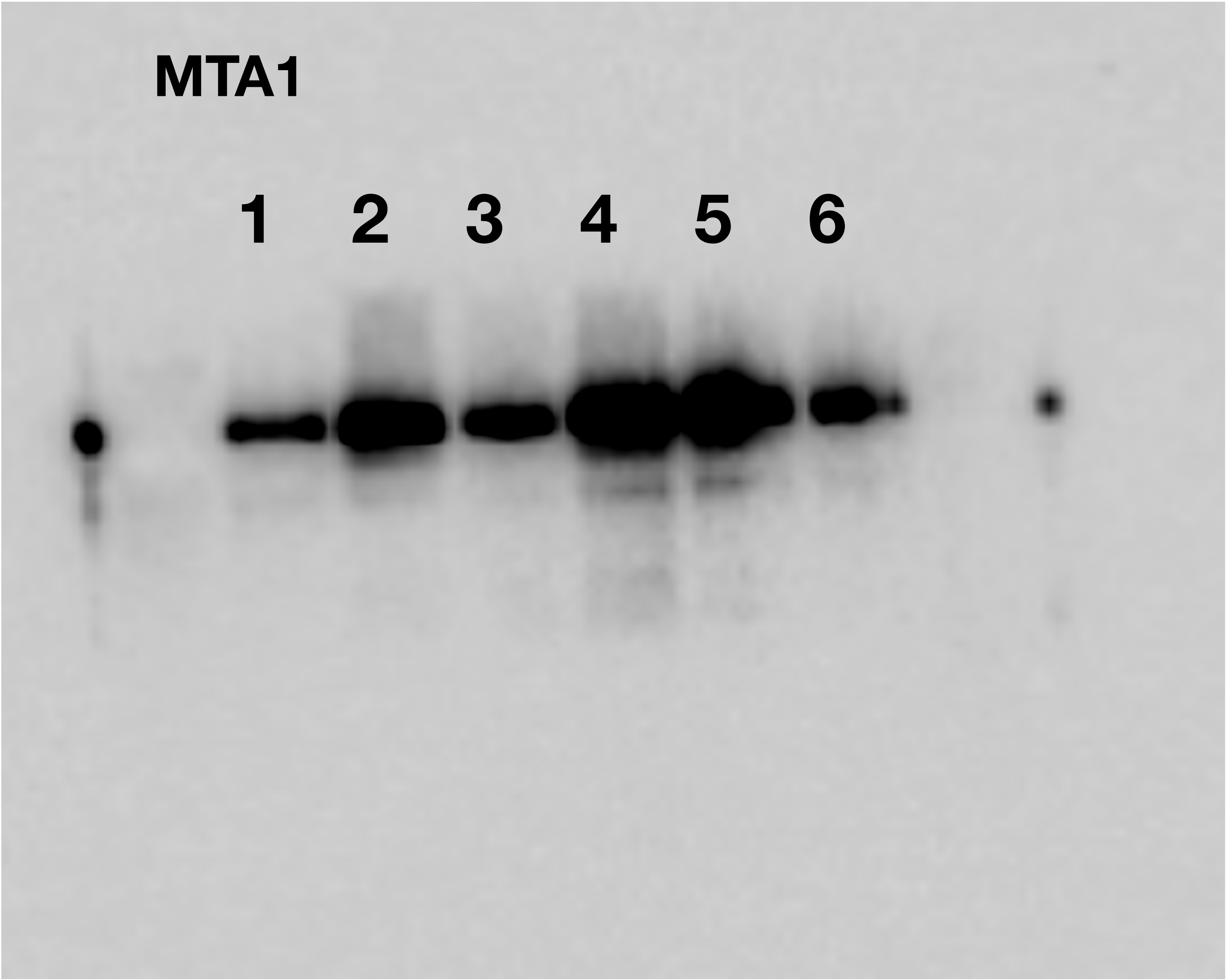

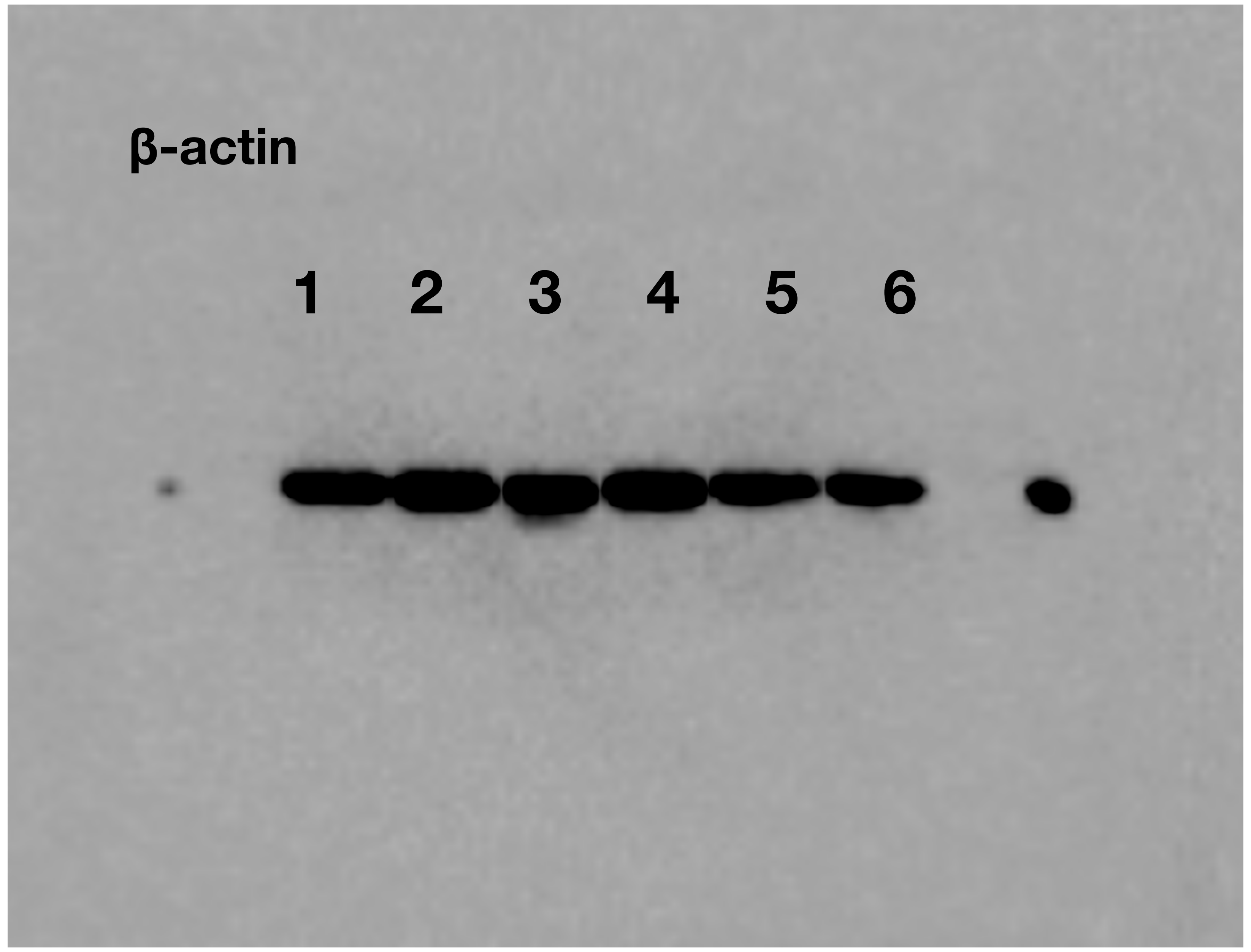

Western blot original bands from Figure 5H, Group 4 bands used for analysis

1 Control
2 LPS
3 LPS+NA
4 LPS+5-AZA
5 LPS+control siRNA
6 LPS+MTA1 siRNA

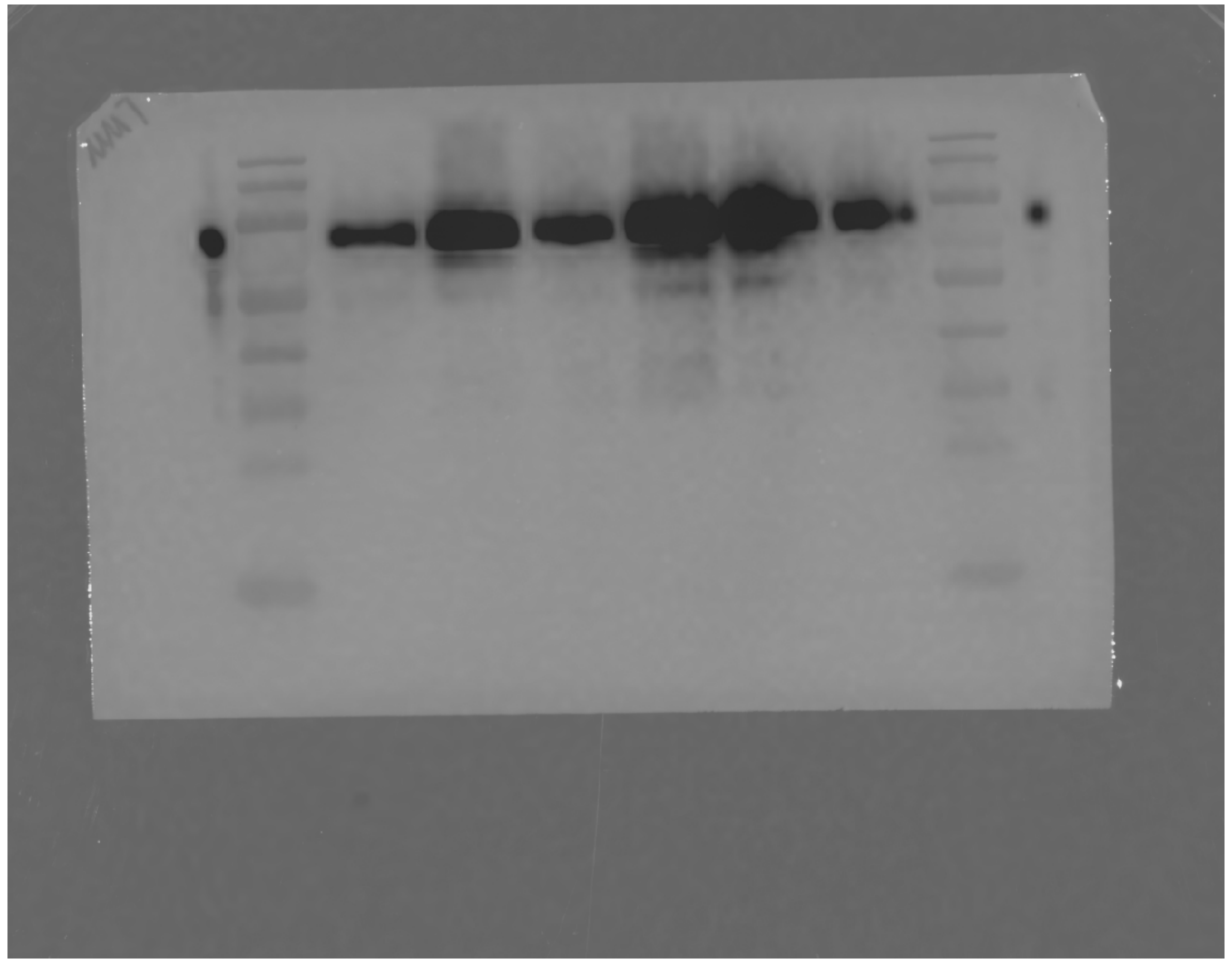

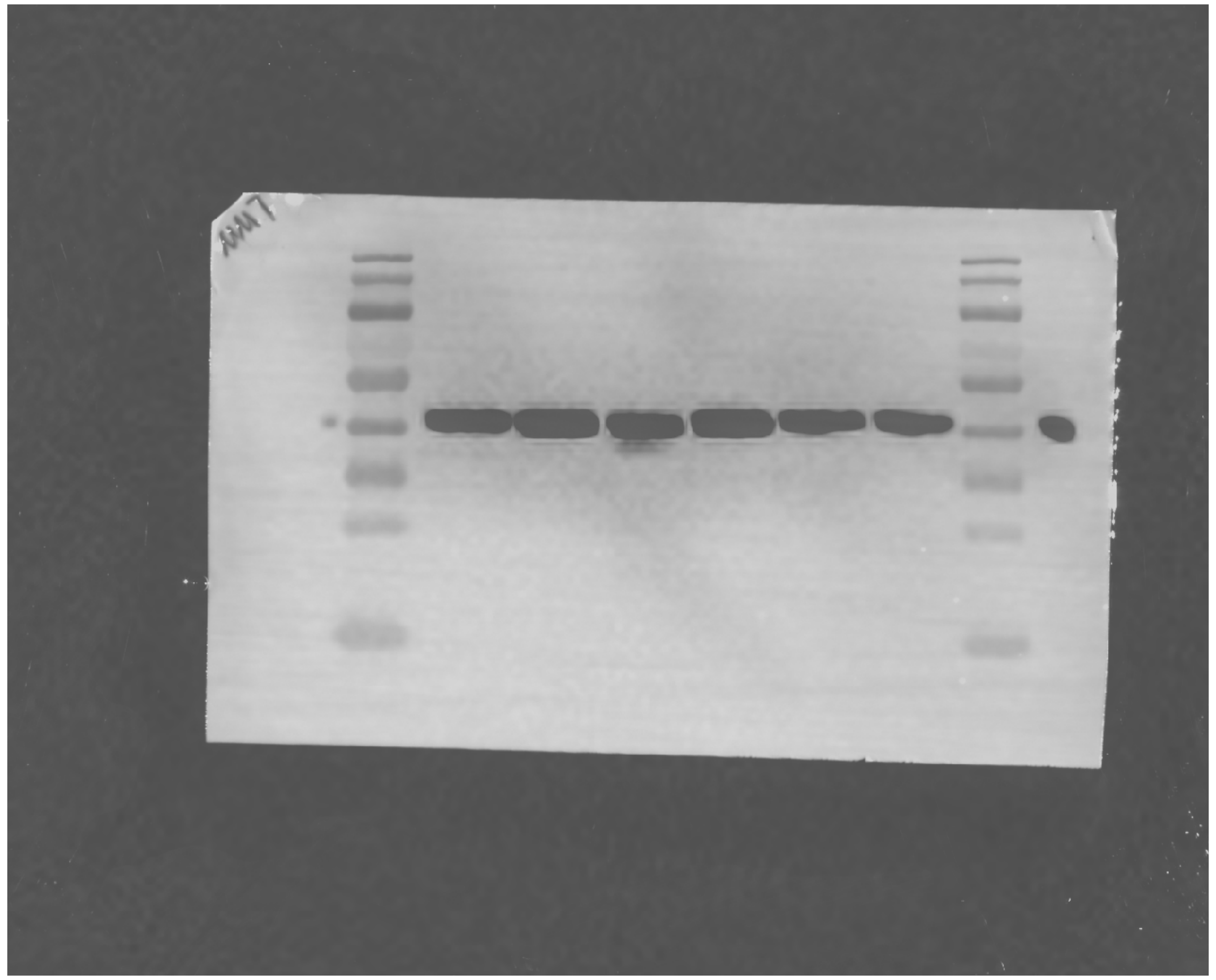

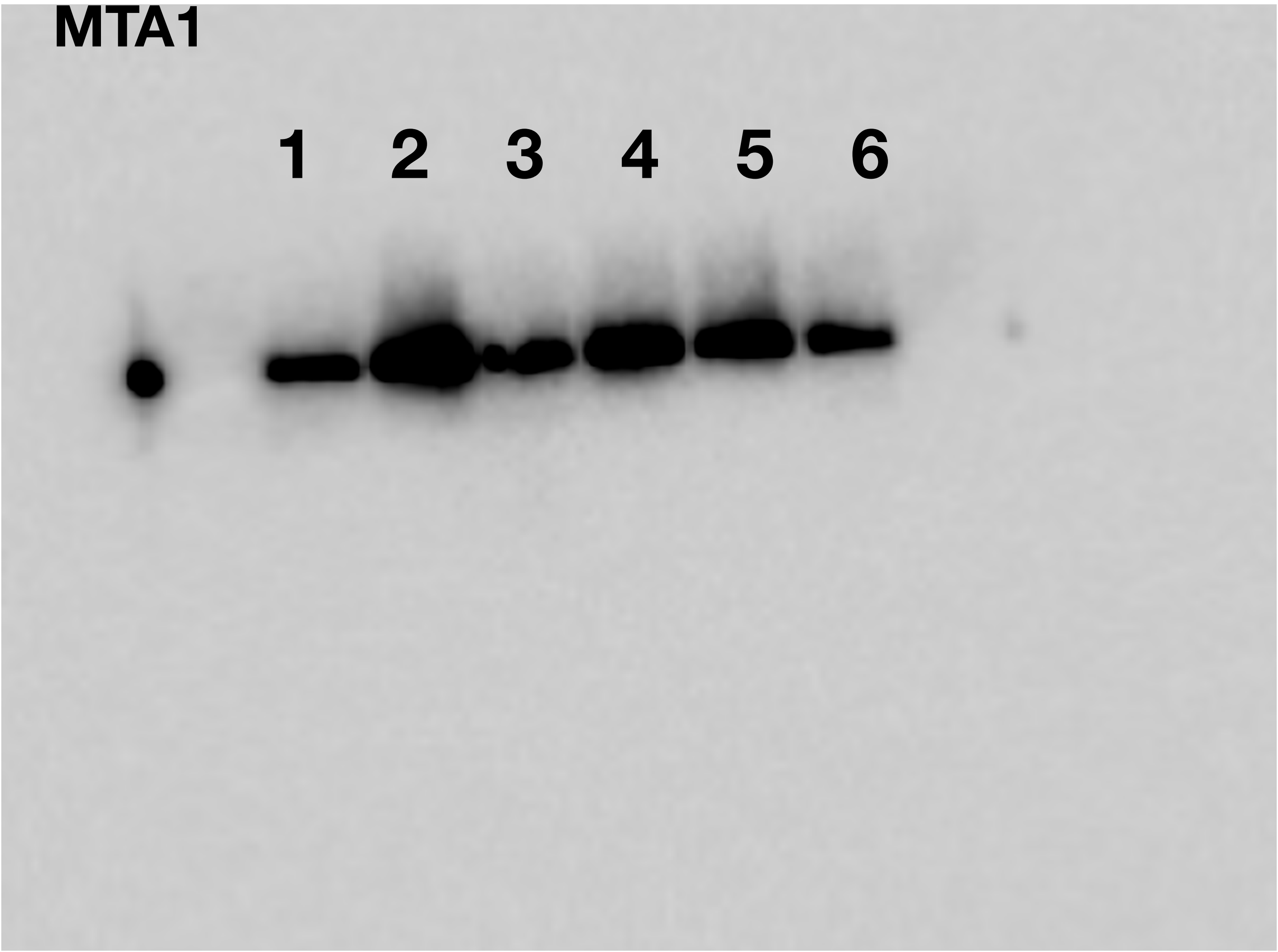

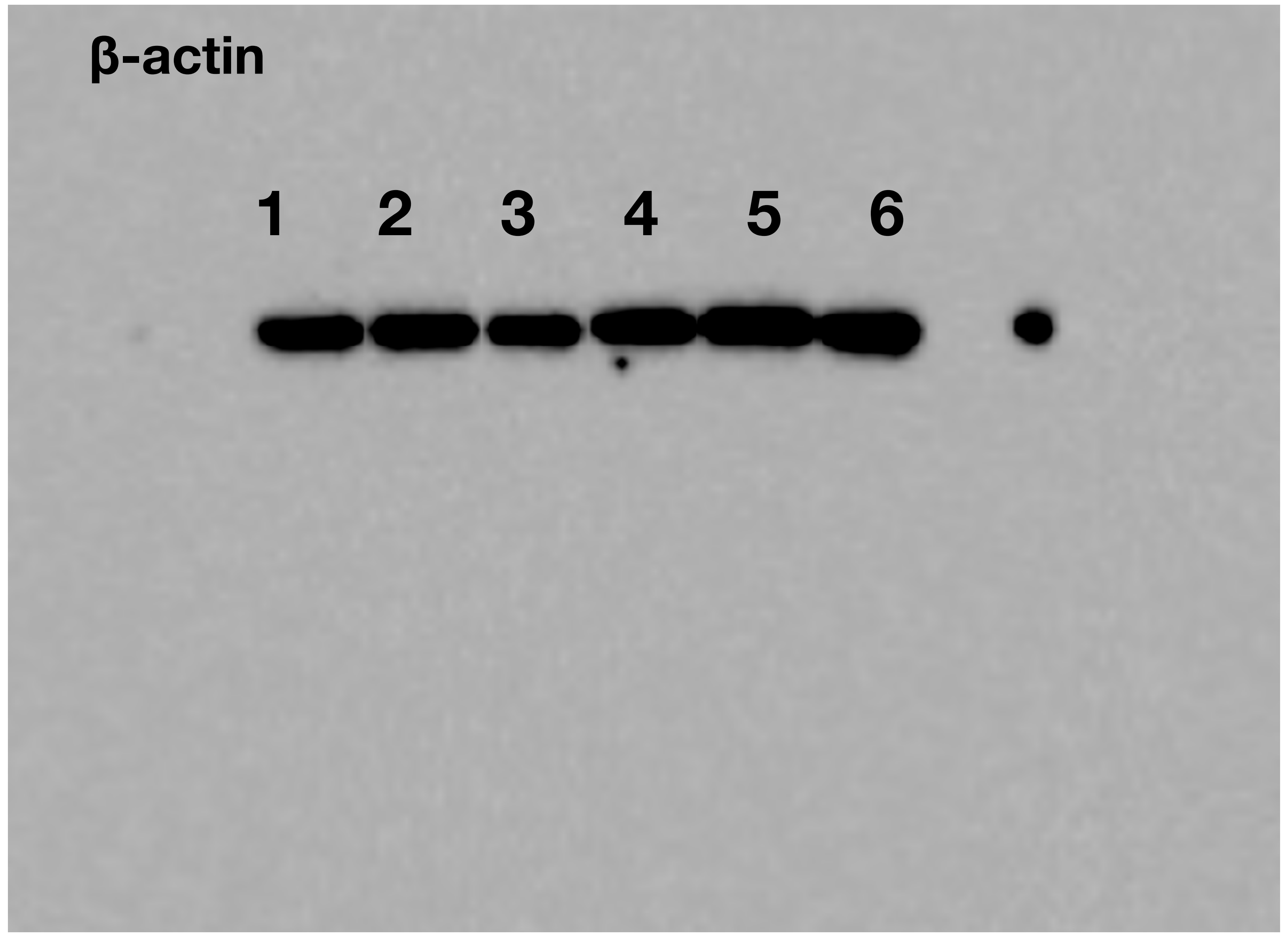

Western blot original bands from Figure 5H, Group 5 bands used for analysis

1 Control
2 LPS
3 LPS+NA
4 LPS+5-AZA
5 LPS+control siRNA
6 LPS+MTA1 siRNA

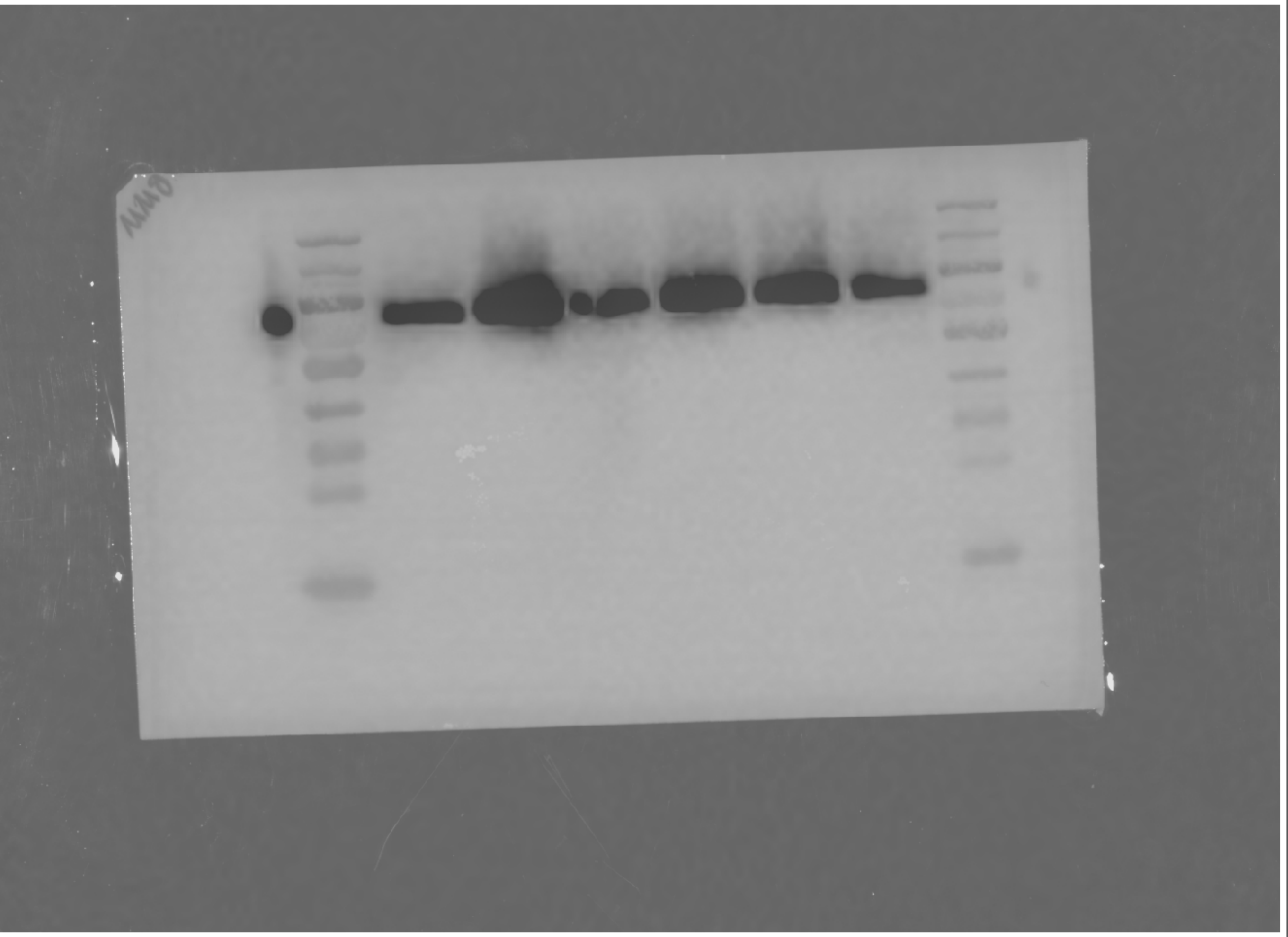

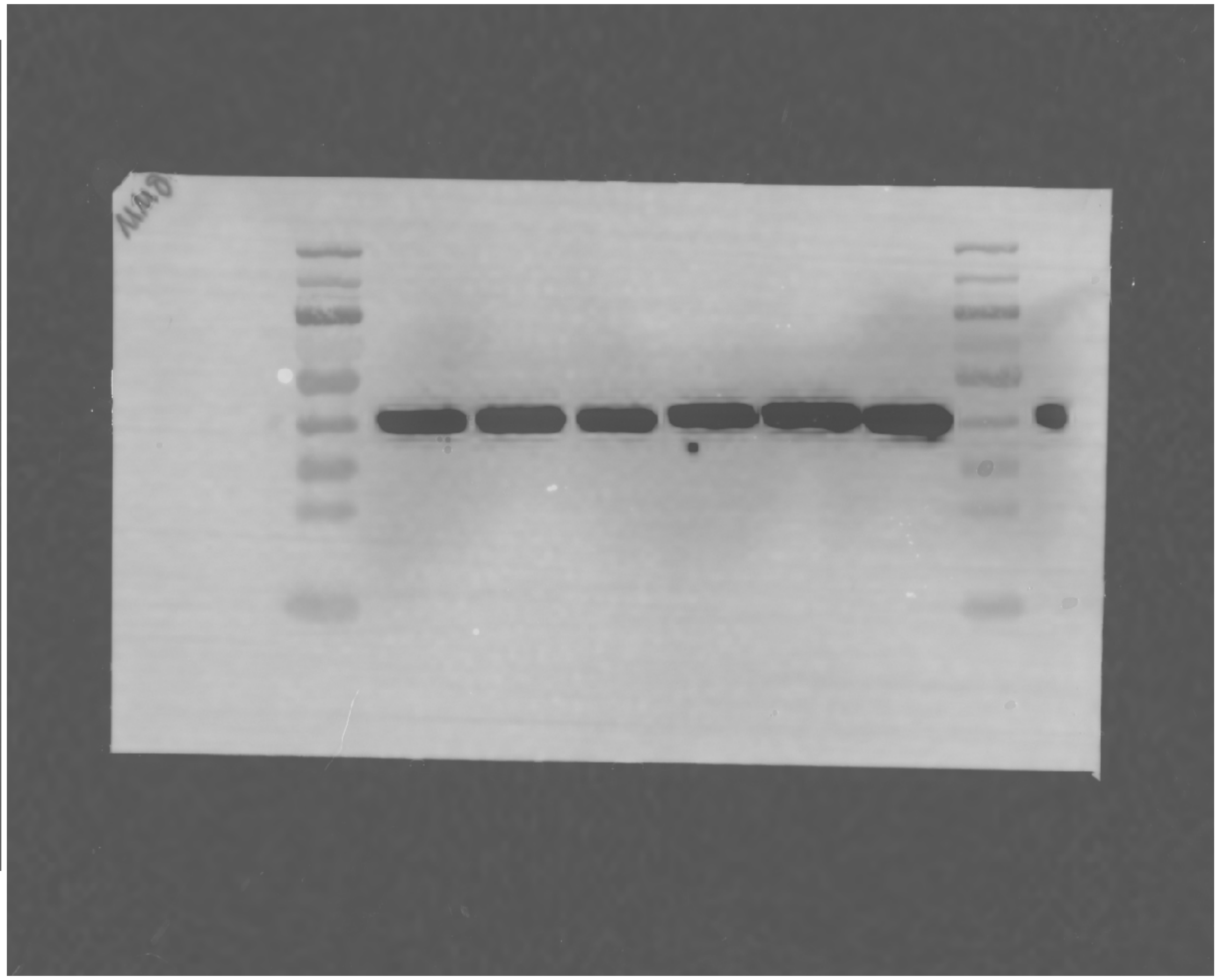

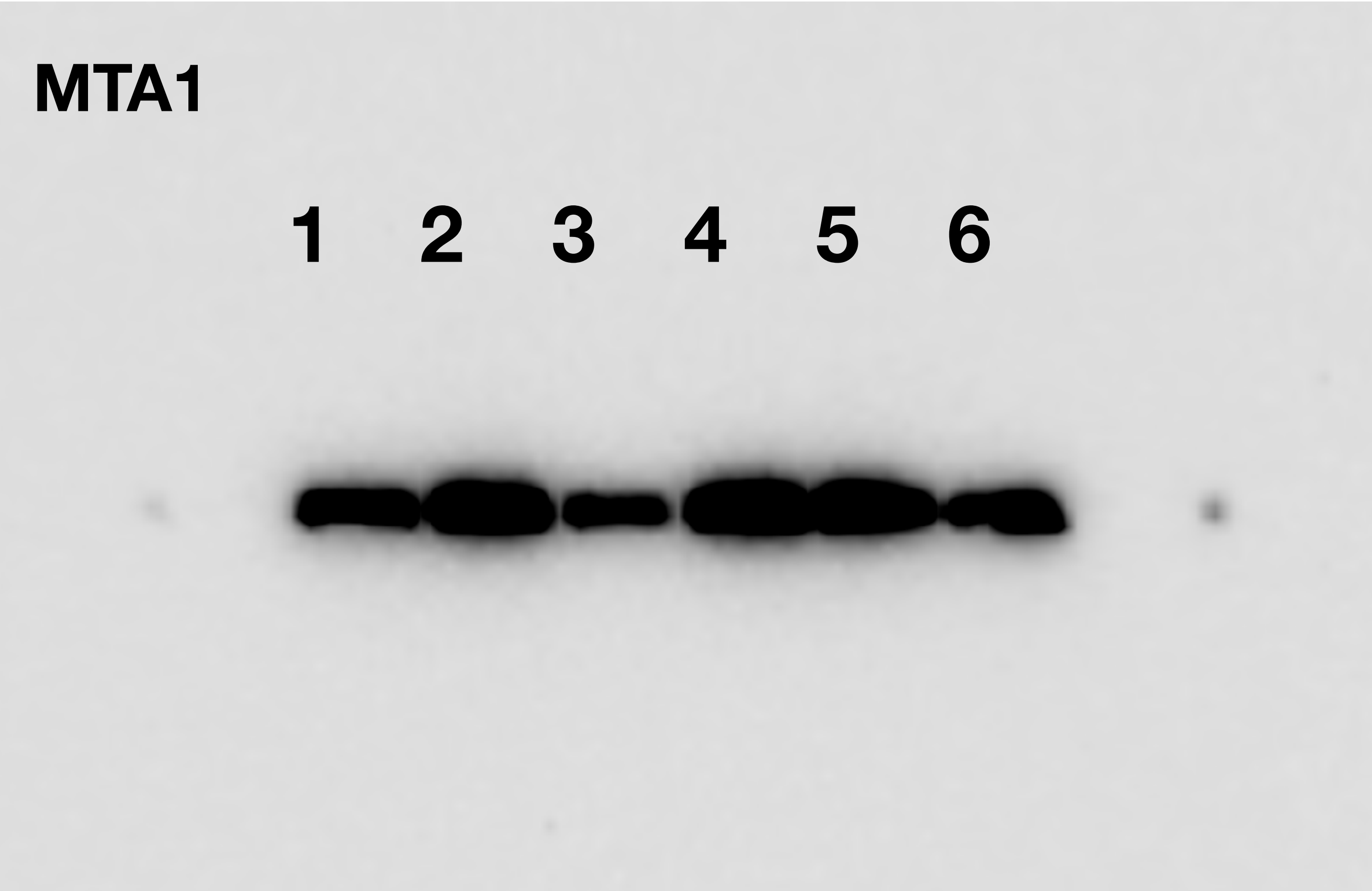

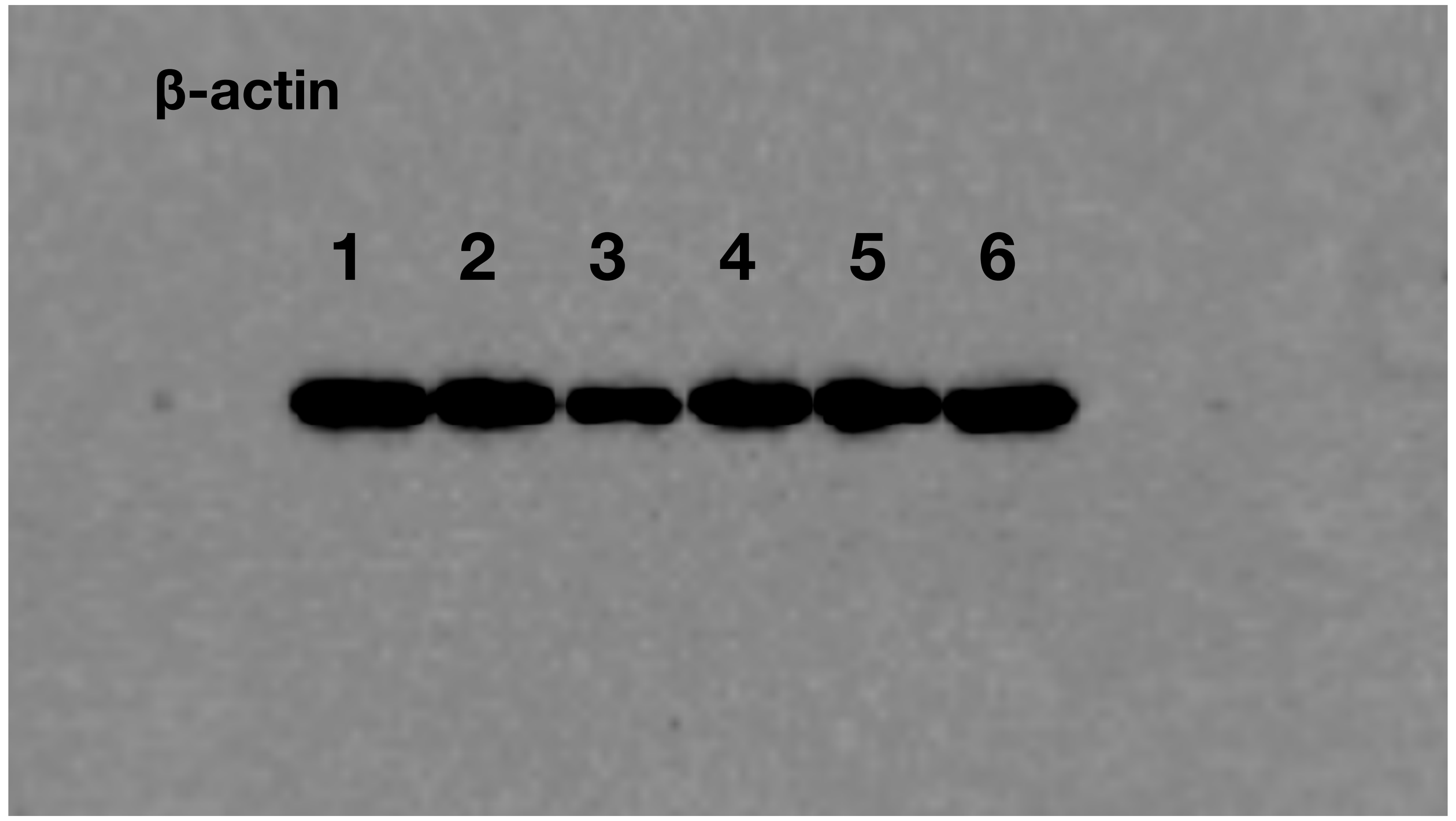

Western blot original bands from Figure 5H, Group 6 bands used for analysis

1 Control
2 LPS
3 LPS+NA
4 LPS+5-AZA
5 LPS+control siRNA
6 LPS+MTA1 siRNA

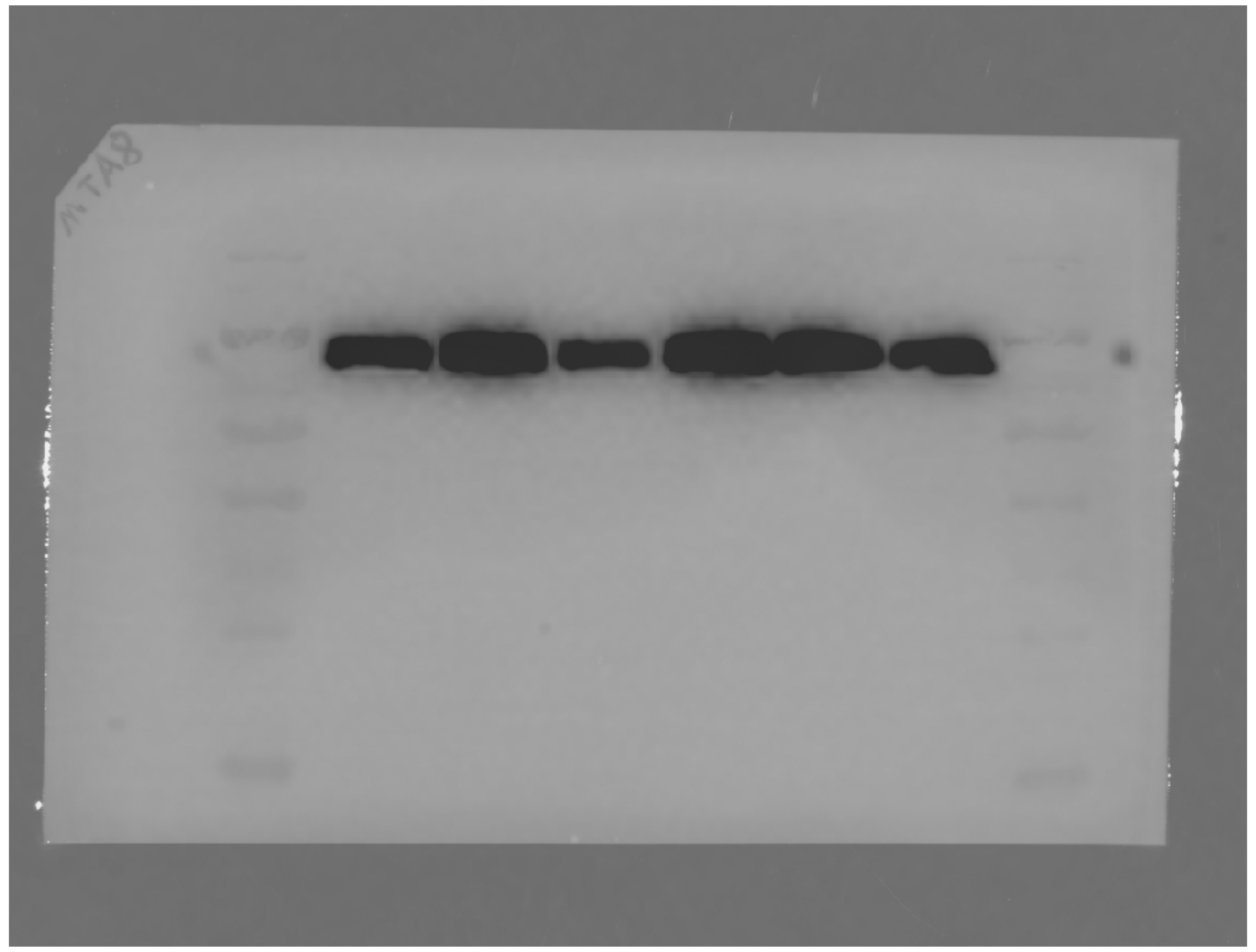

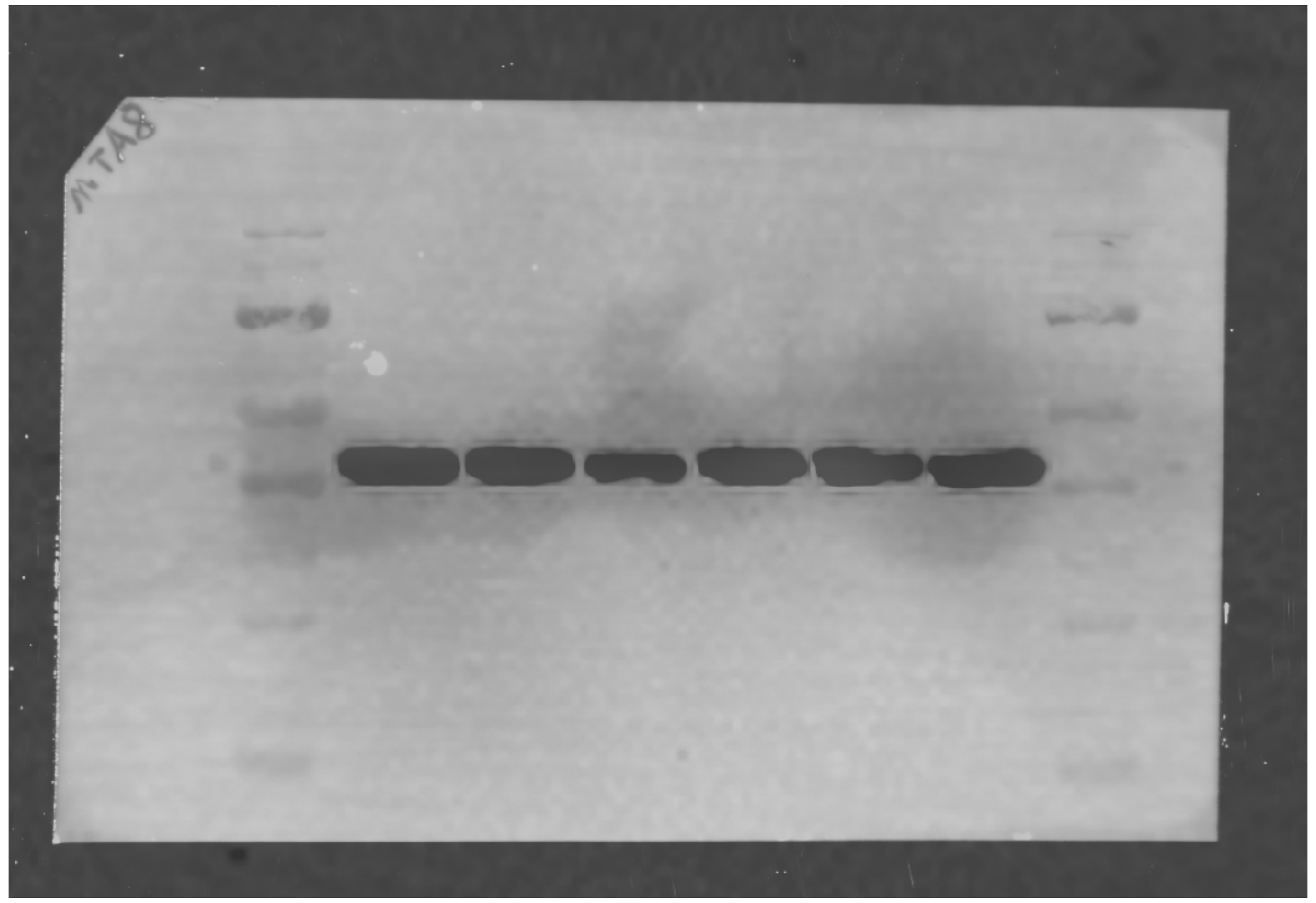

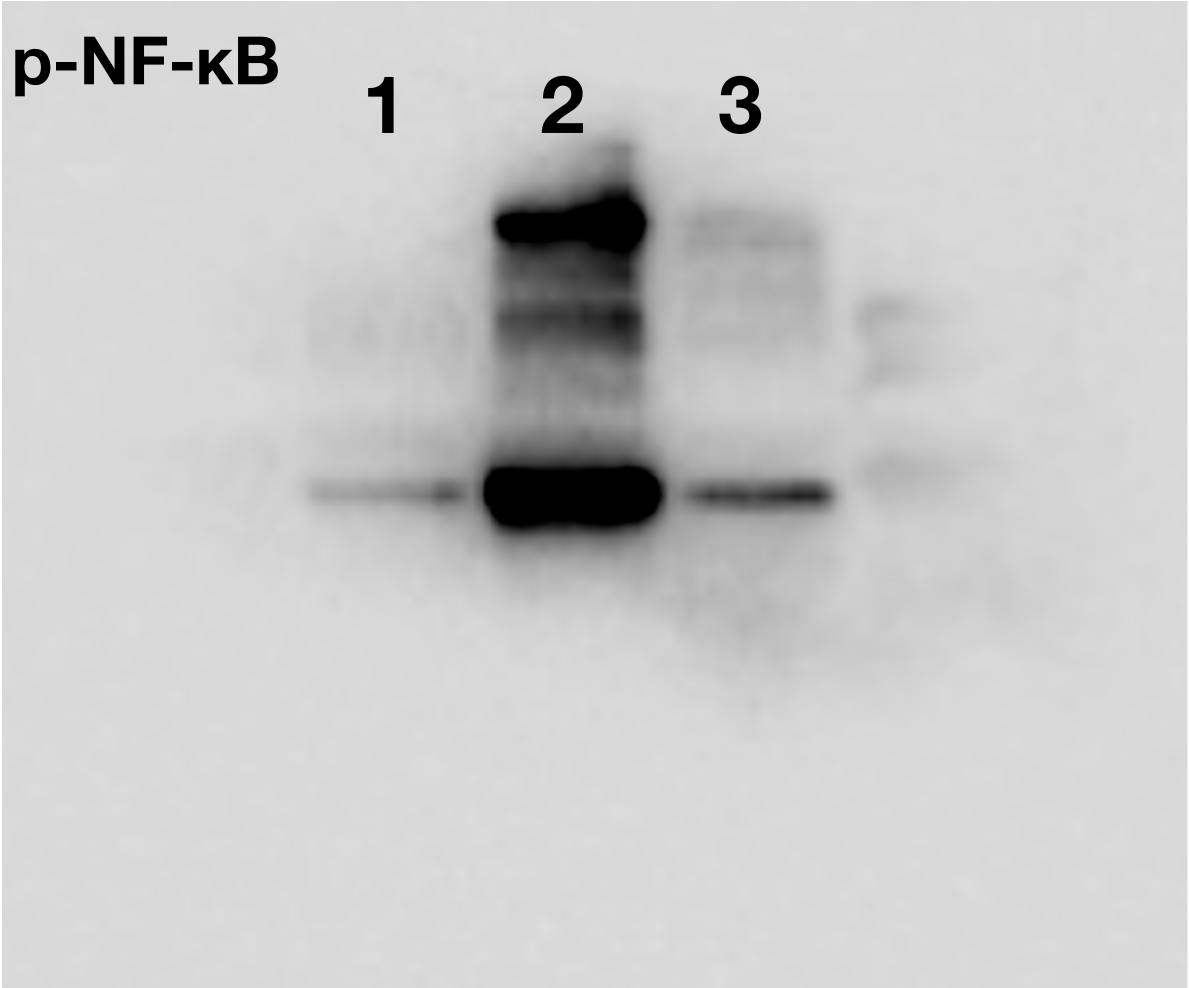

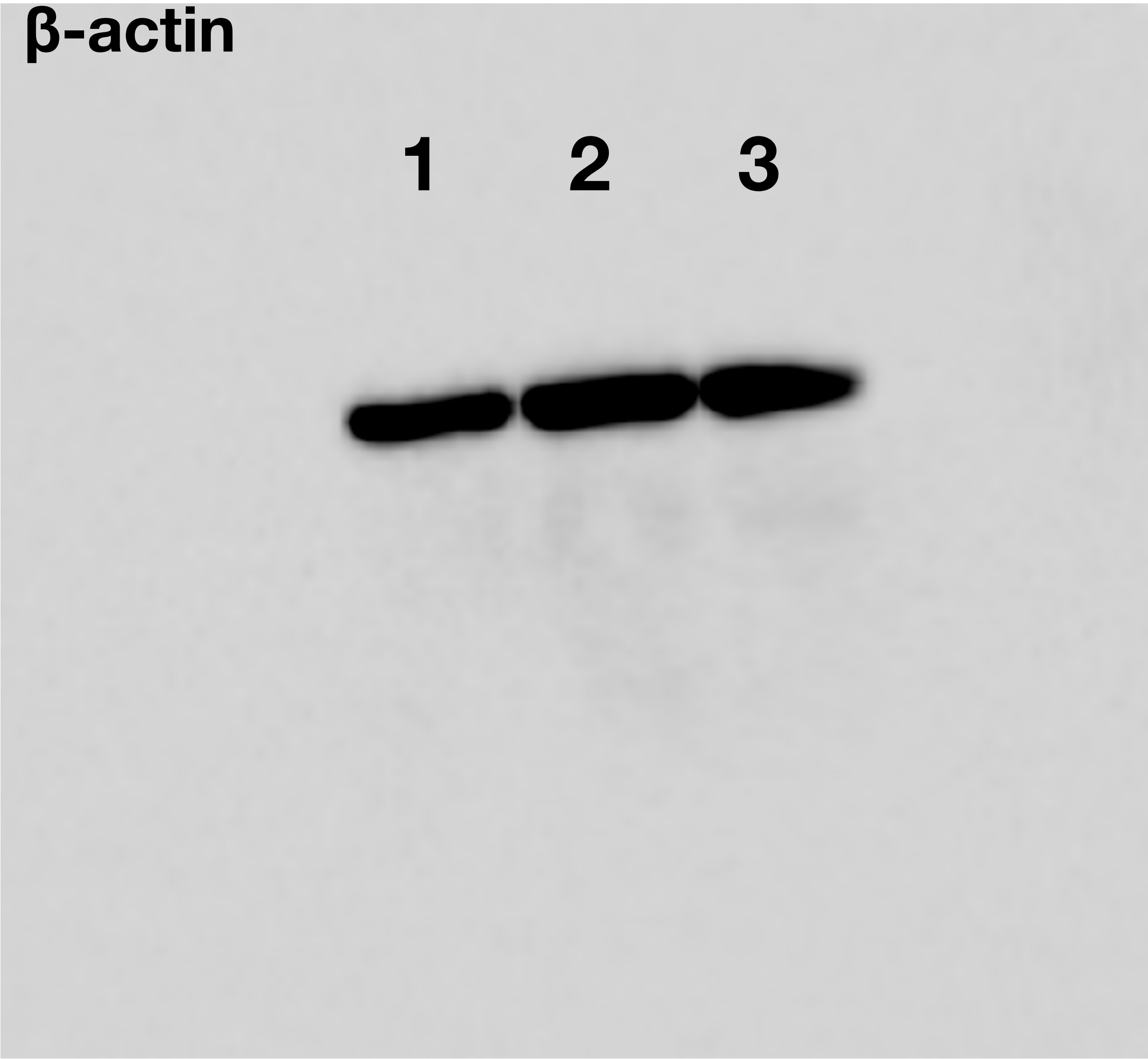

Western blot original bands from Figure 6A, Group 1 bands used for analysis

1 Control
2 LPS
3 LPS+NA

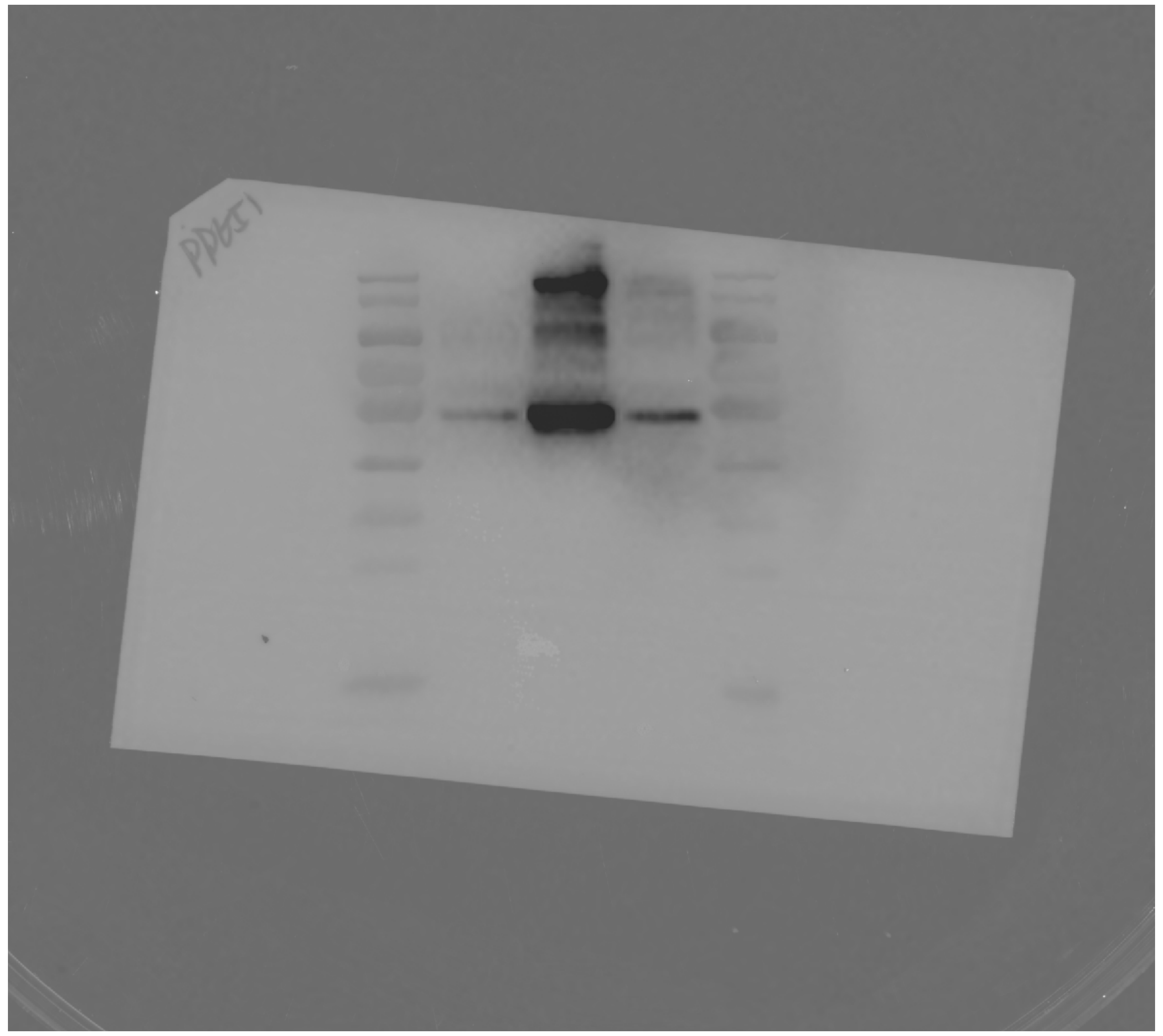

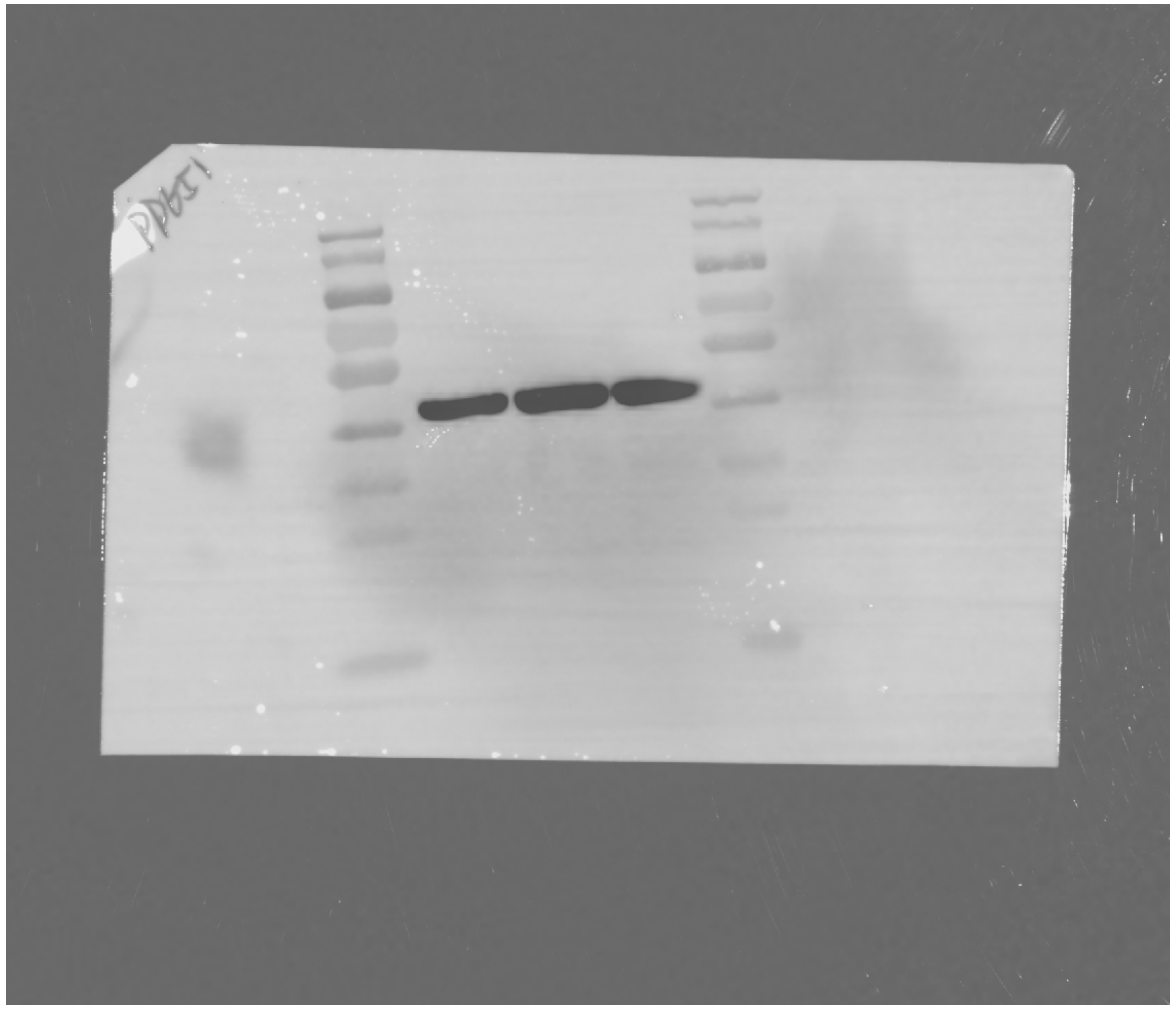

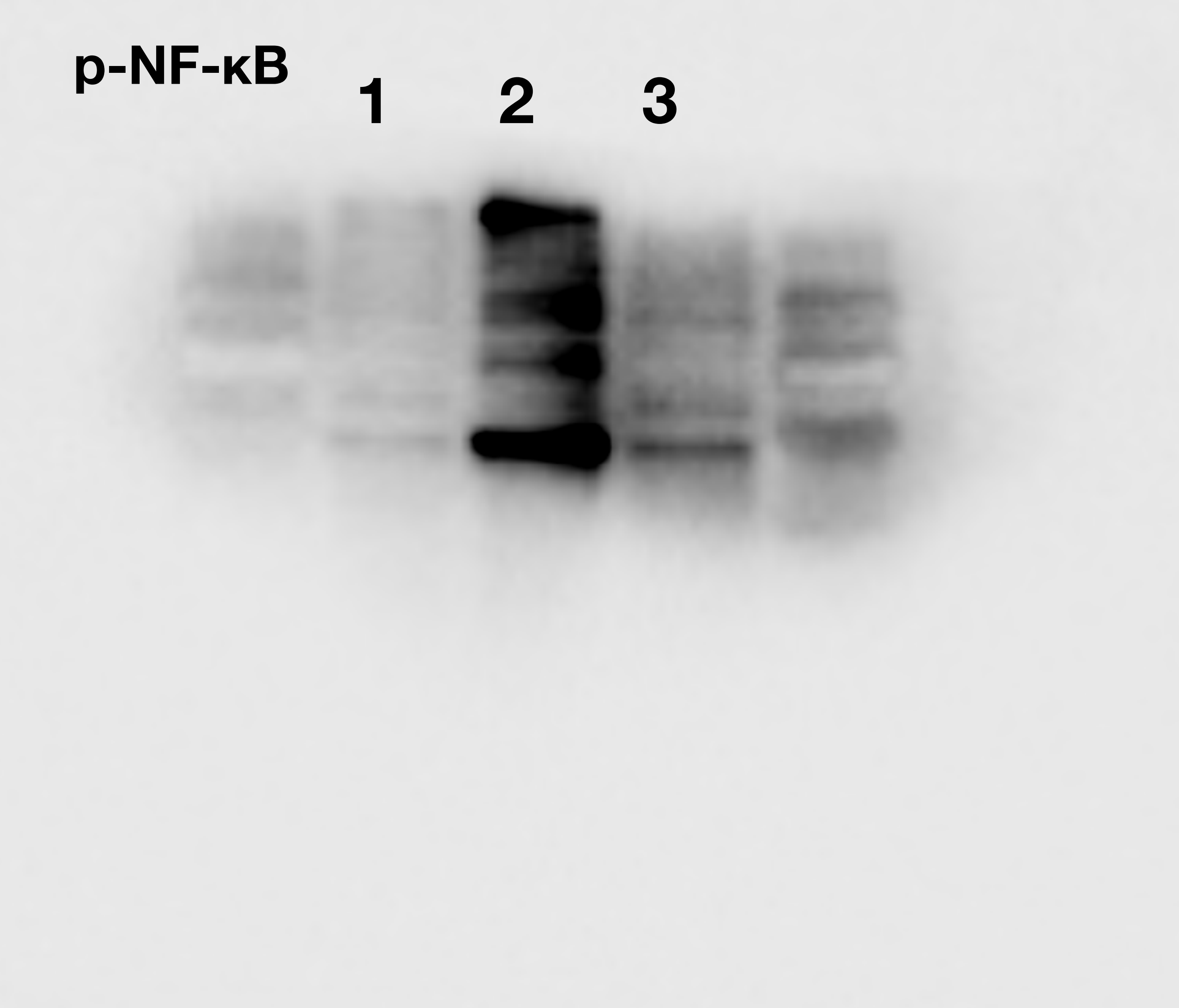

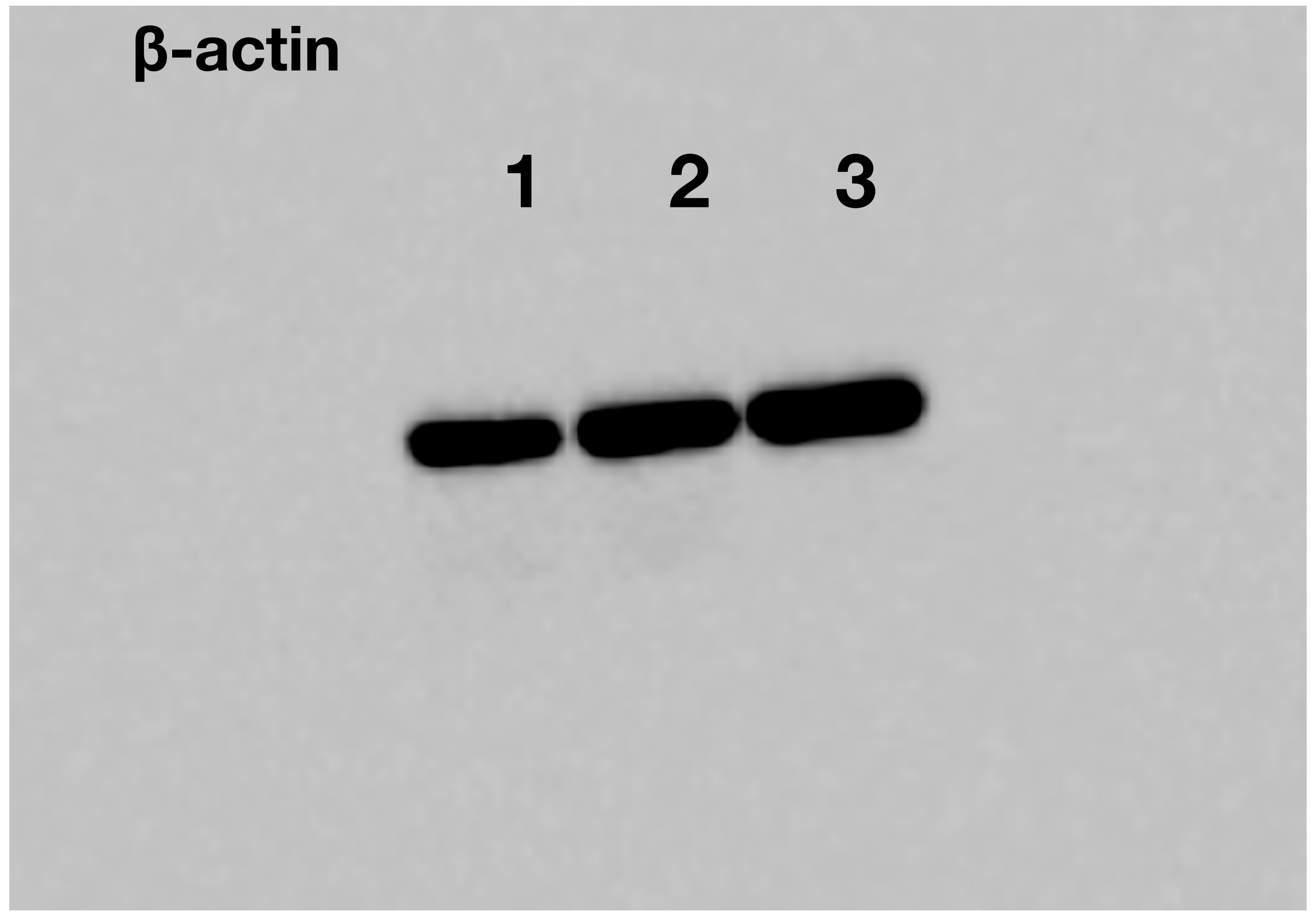

Western blot original bands from Figure 6A, Group 2 bands used for analysis

1 Control
2 LPS
3 LPS+NA

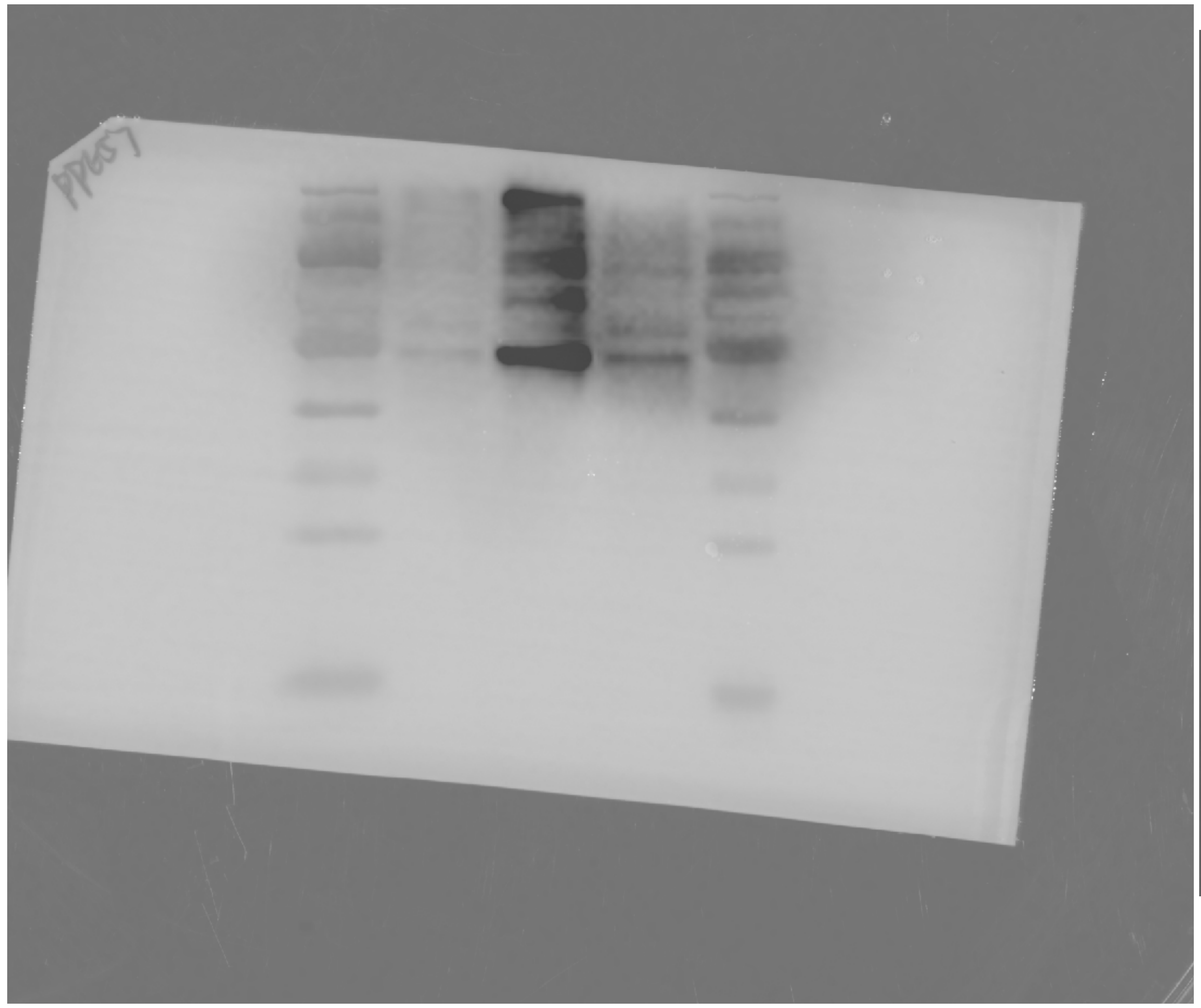

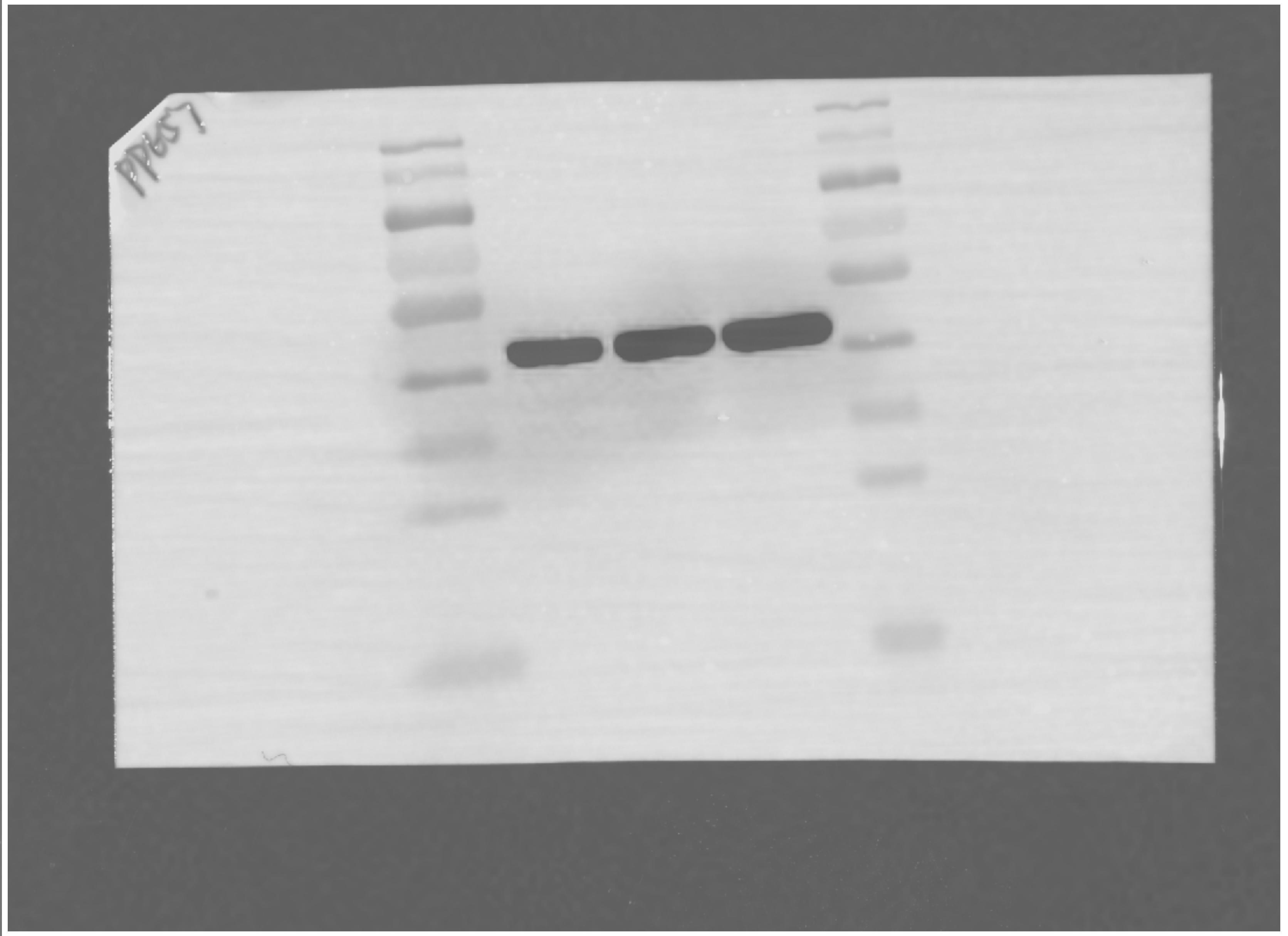

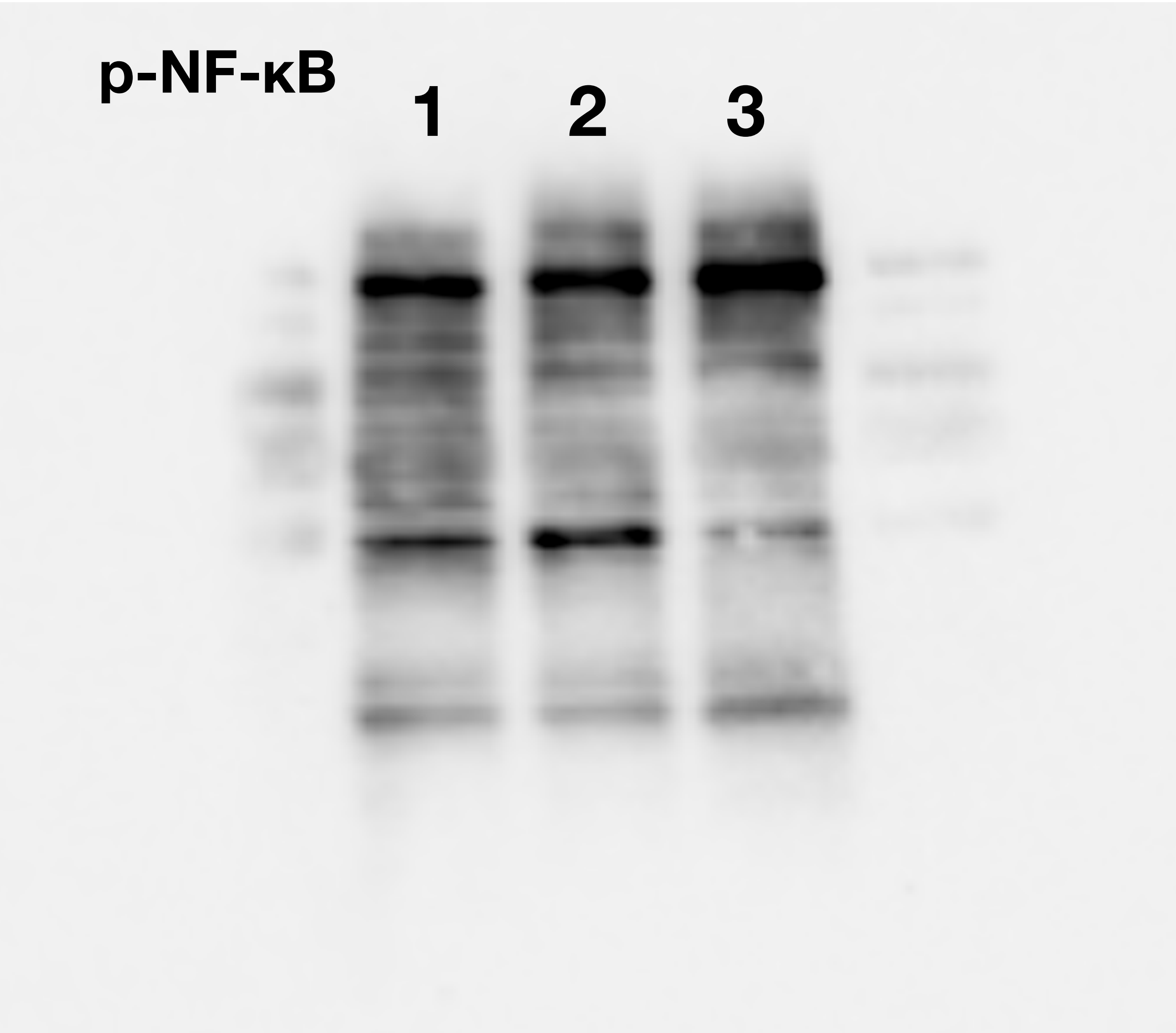

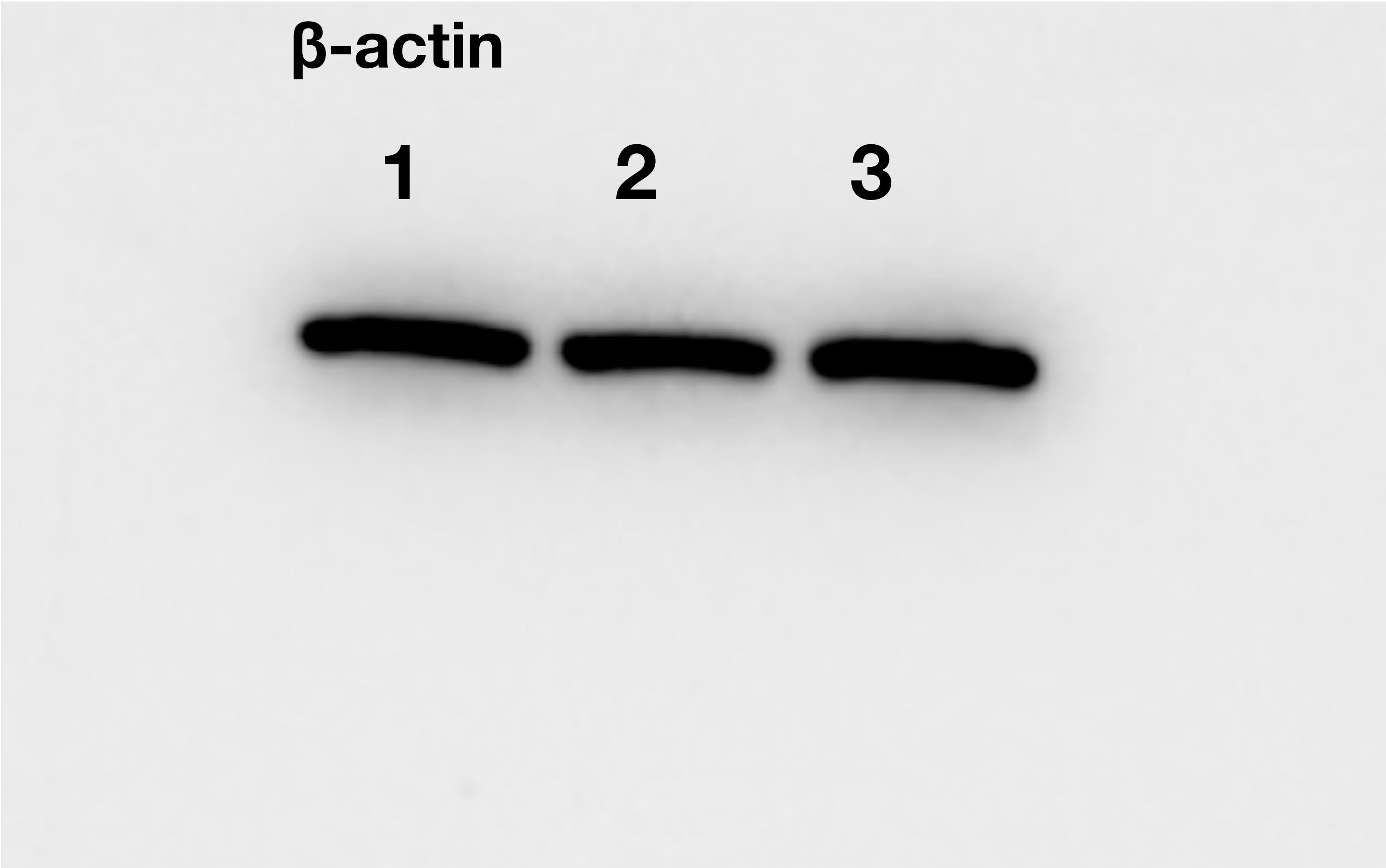

Western blot original bands from Figure 6A, Group 3 bands used for analysis

1 Control
2 LPS
3 LPS+NA

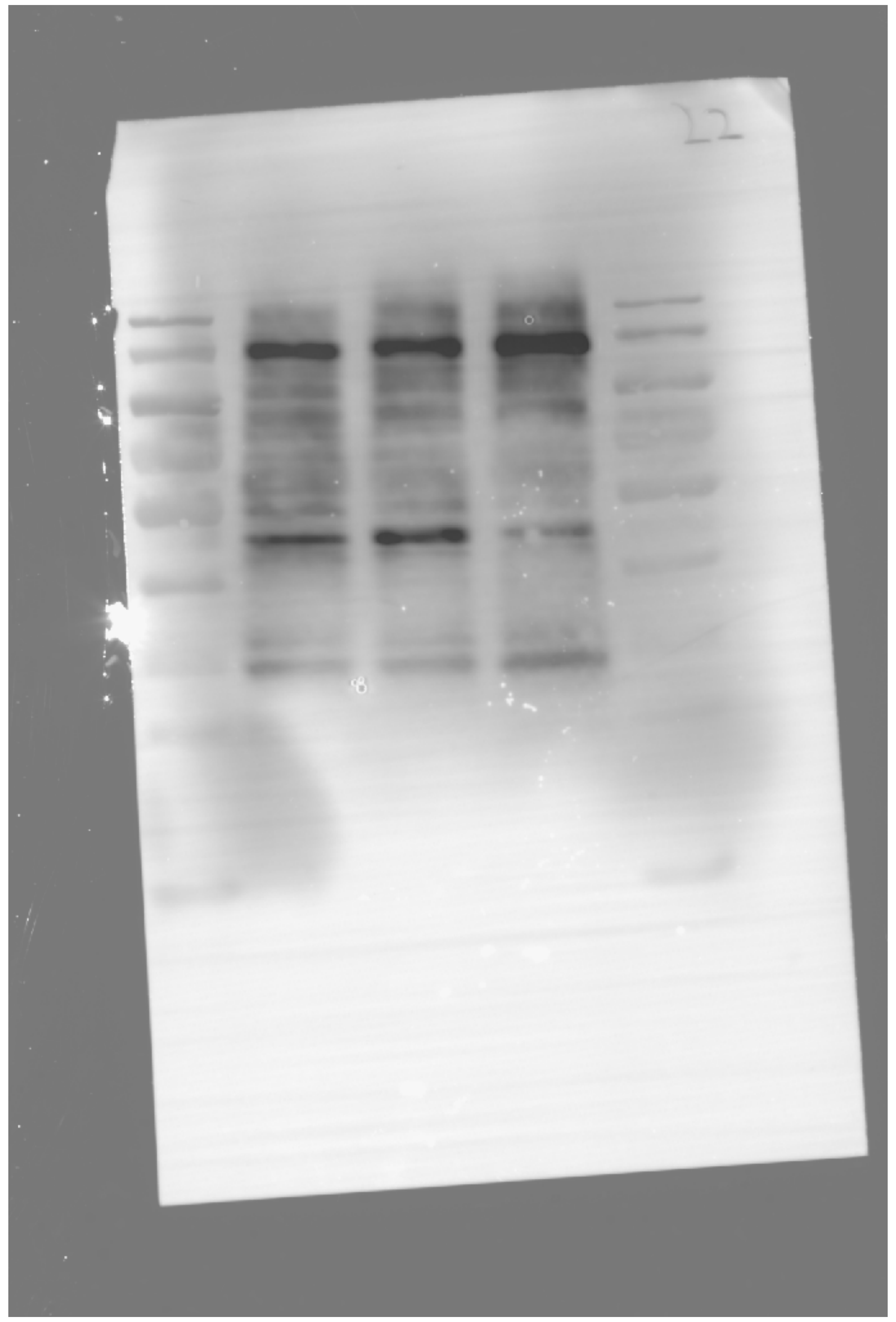

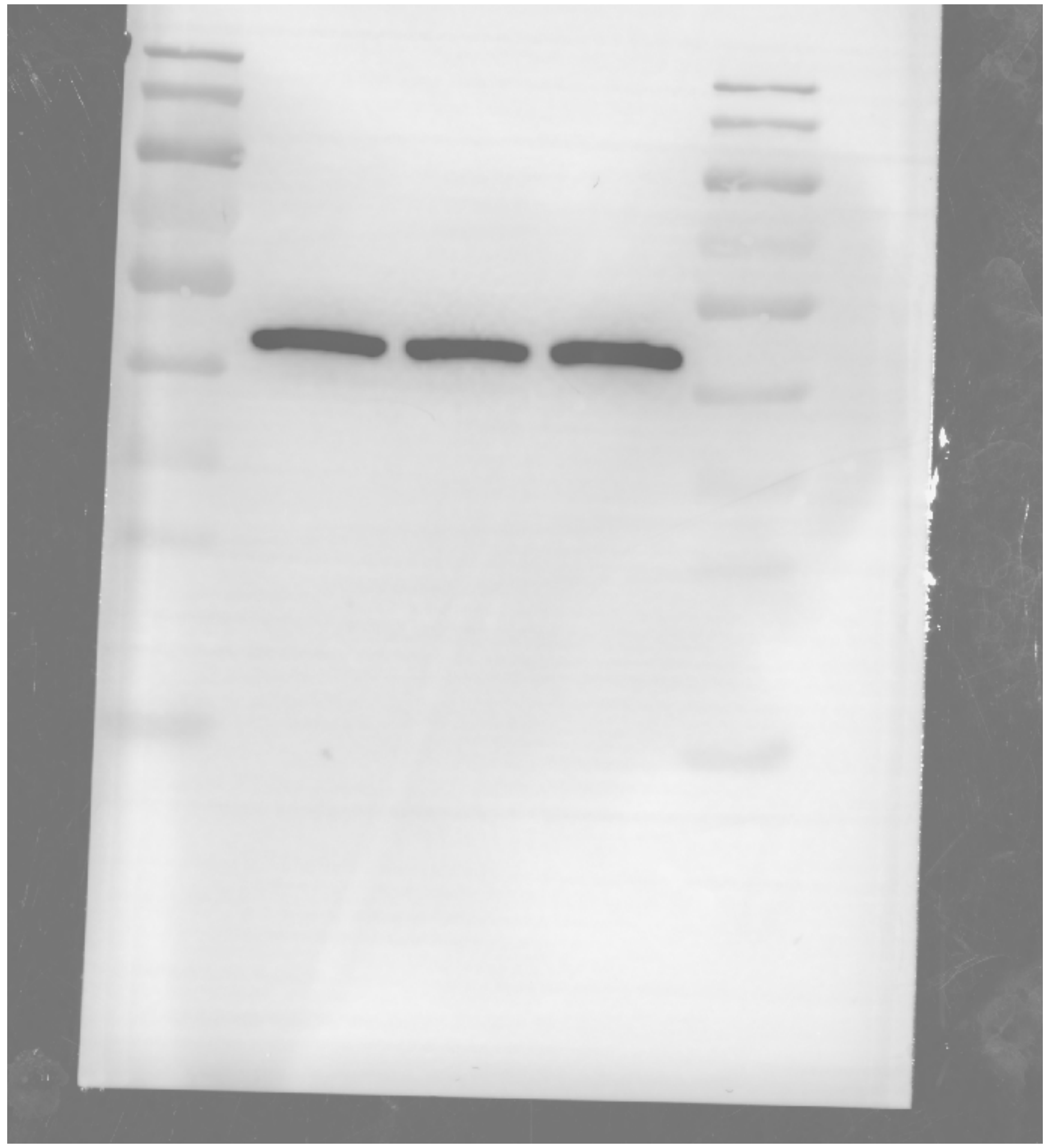

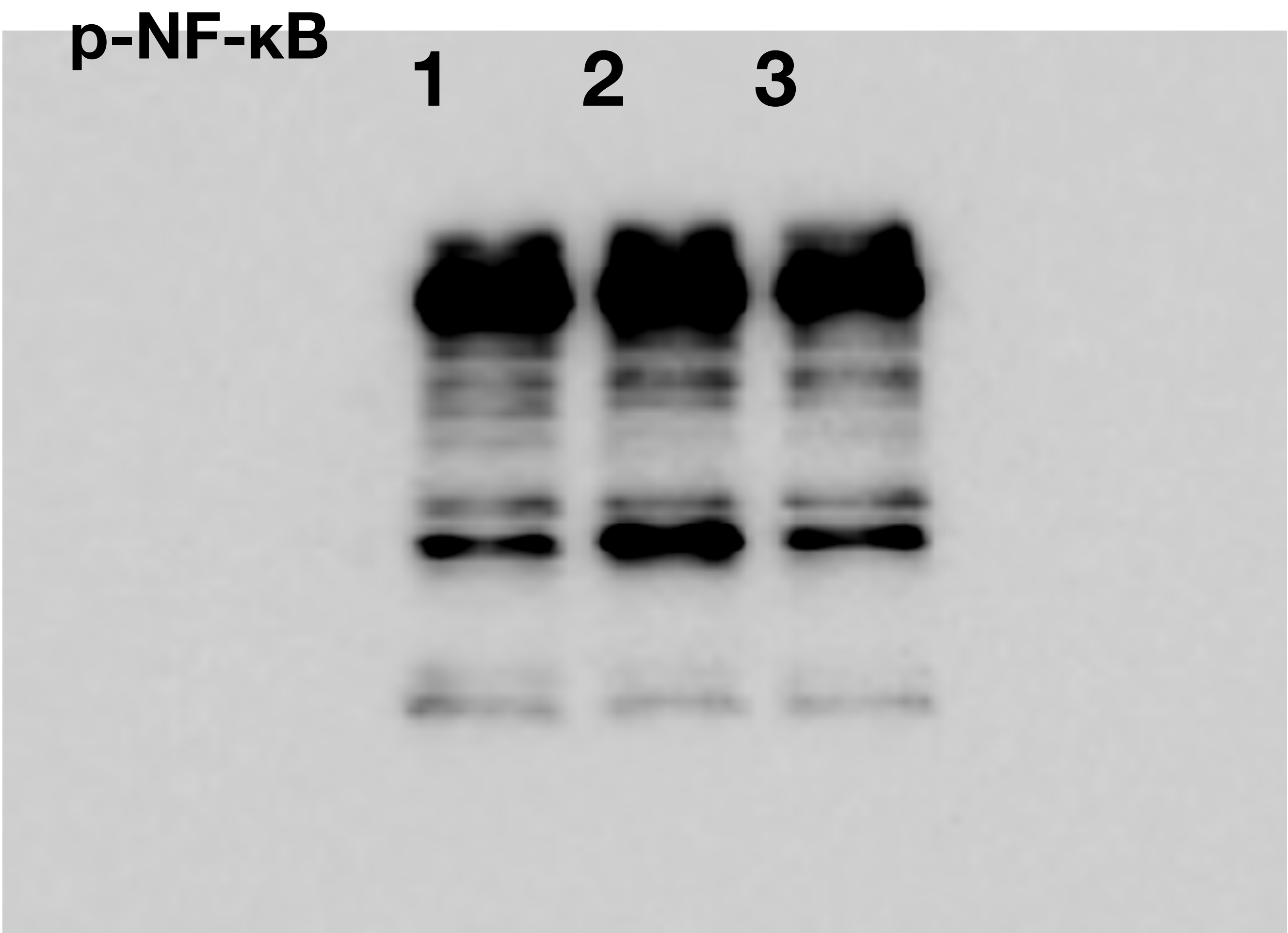

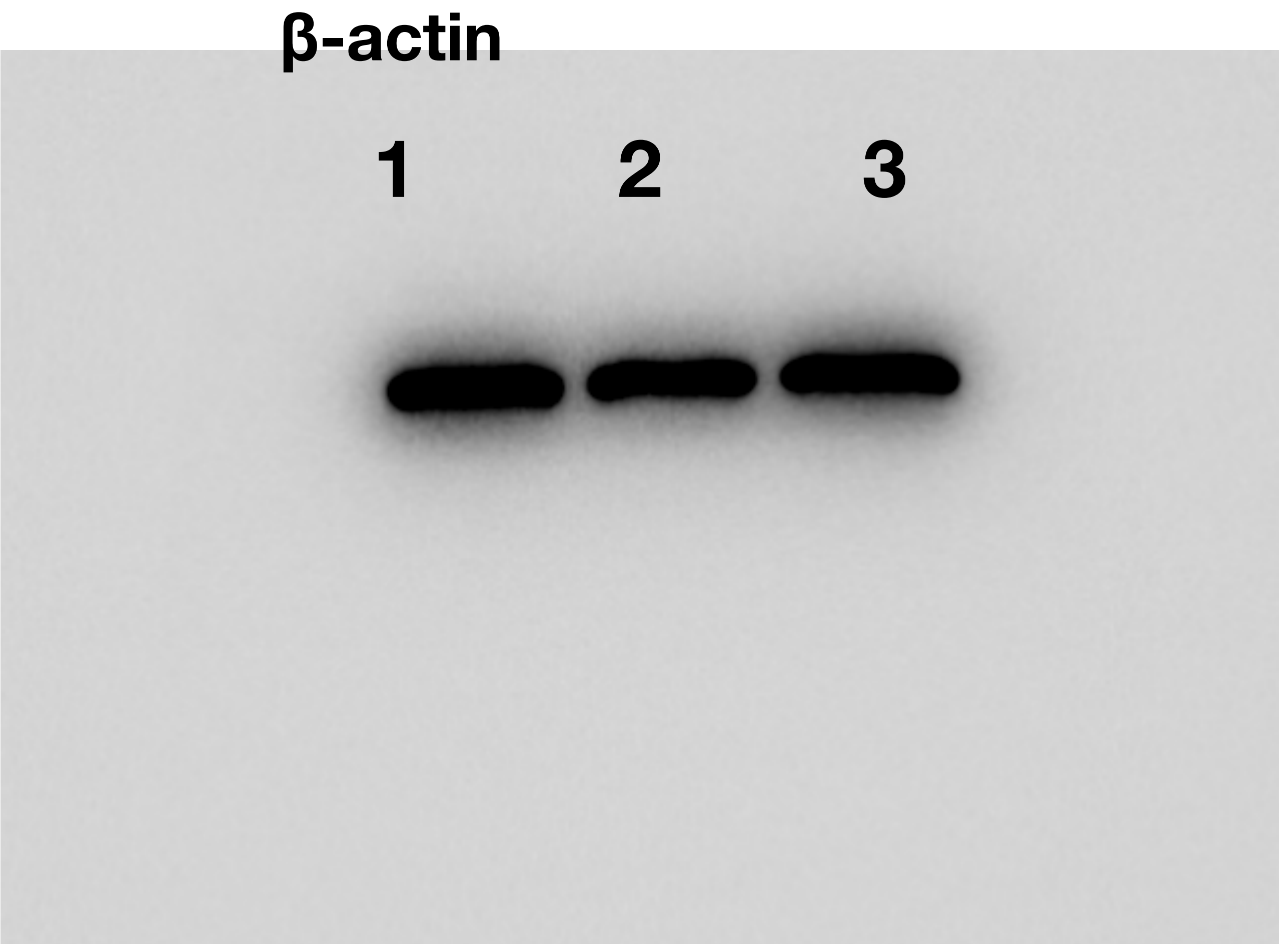

Western blot original bands from Figure 6D, Group 1 bands used for analysis

1 Control
2 LPS+control siRNA
3 LPS+MTA1 siRNA

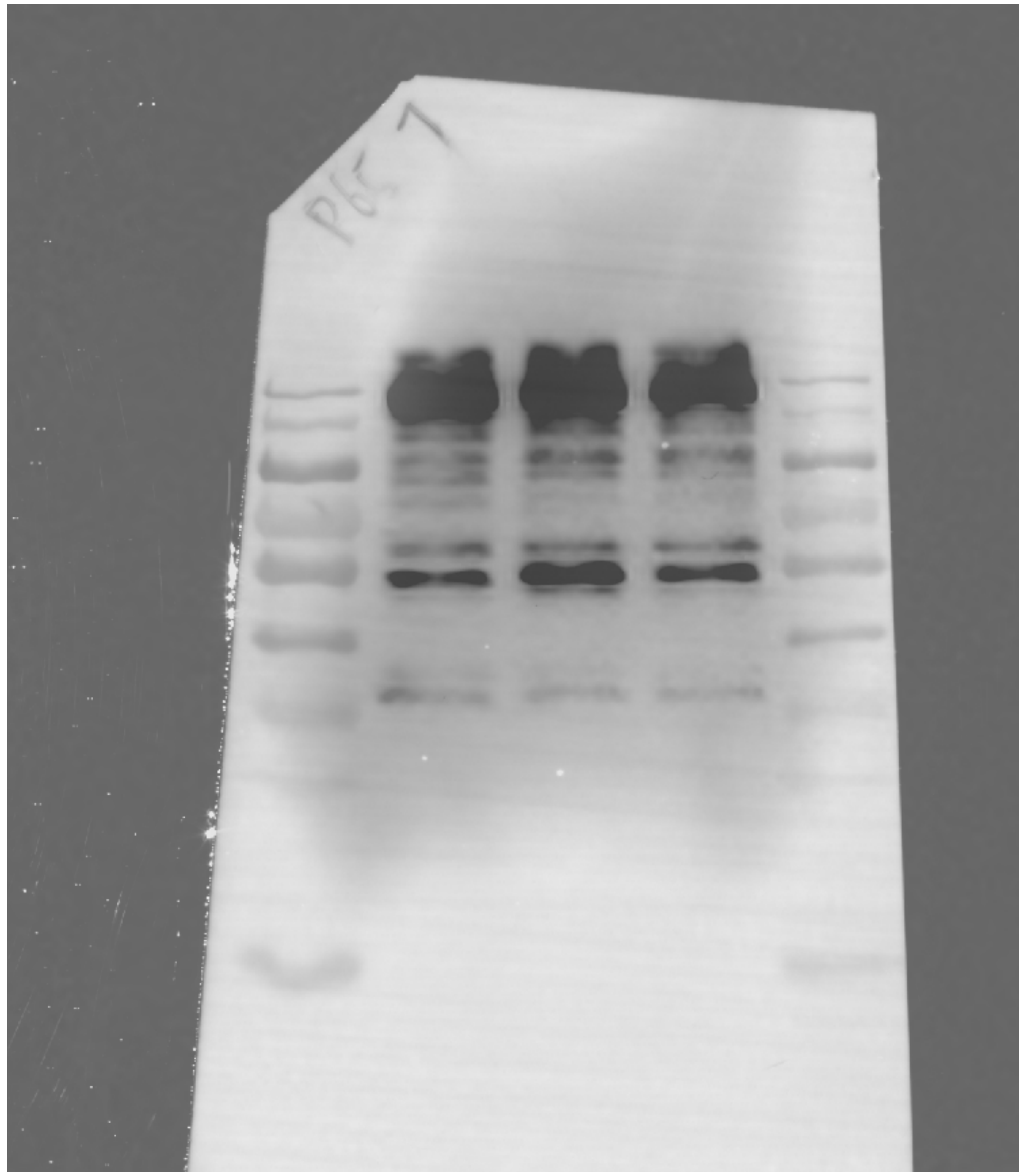

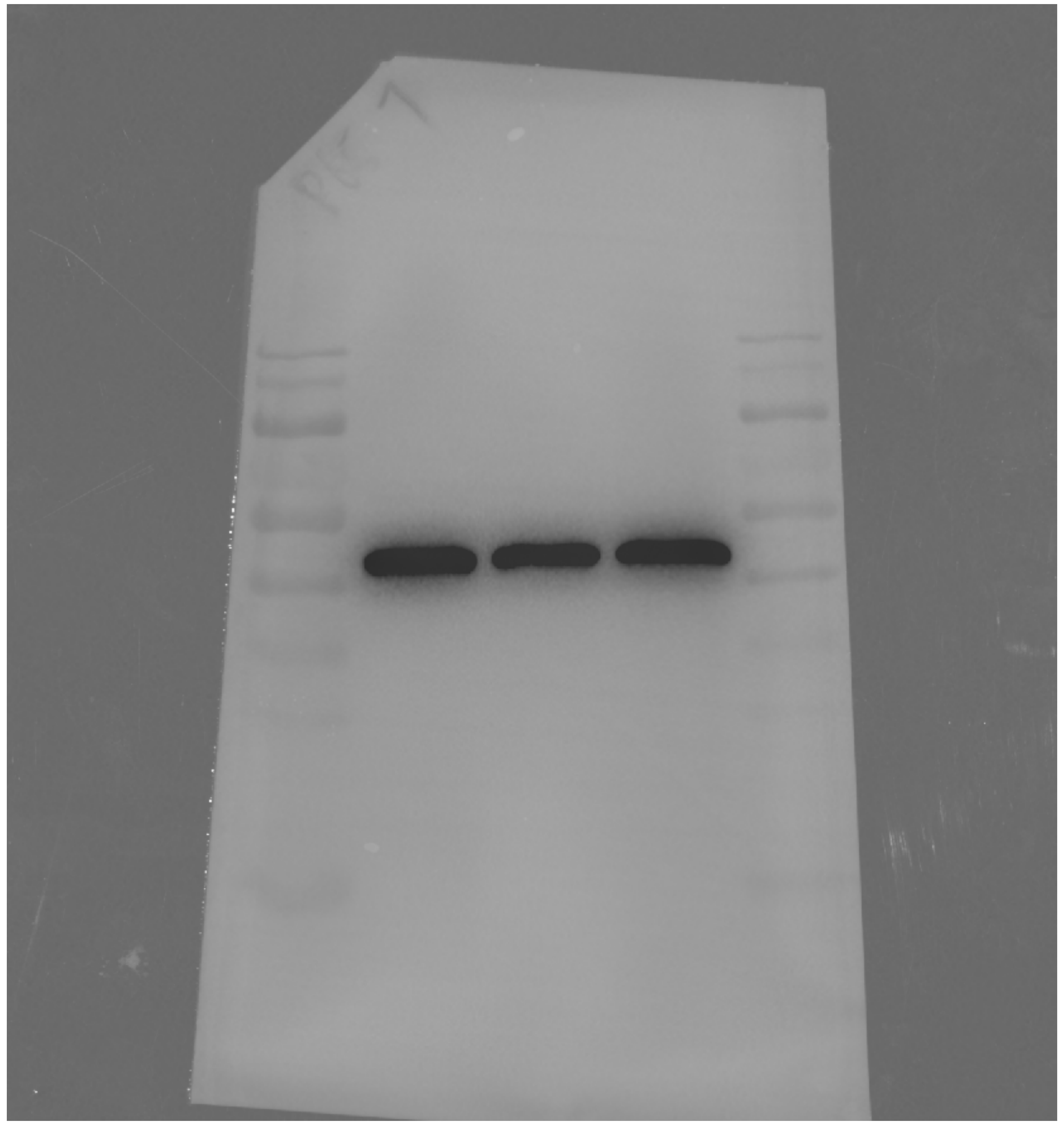

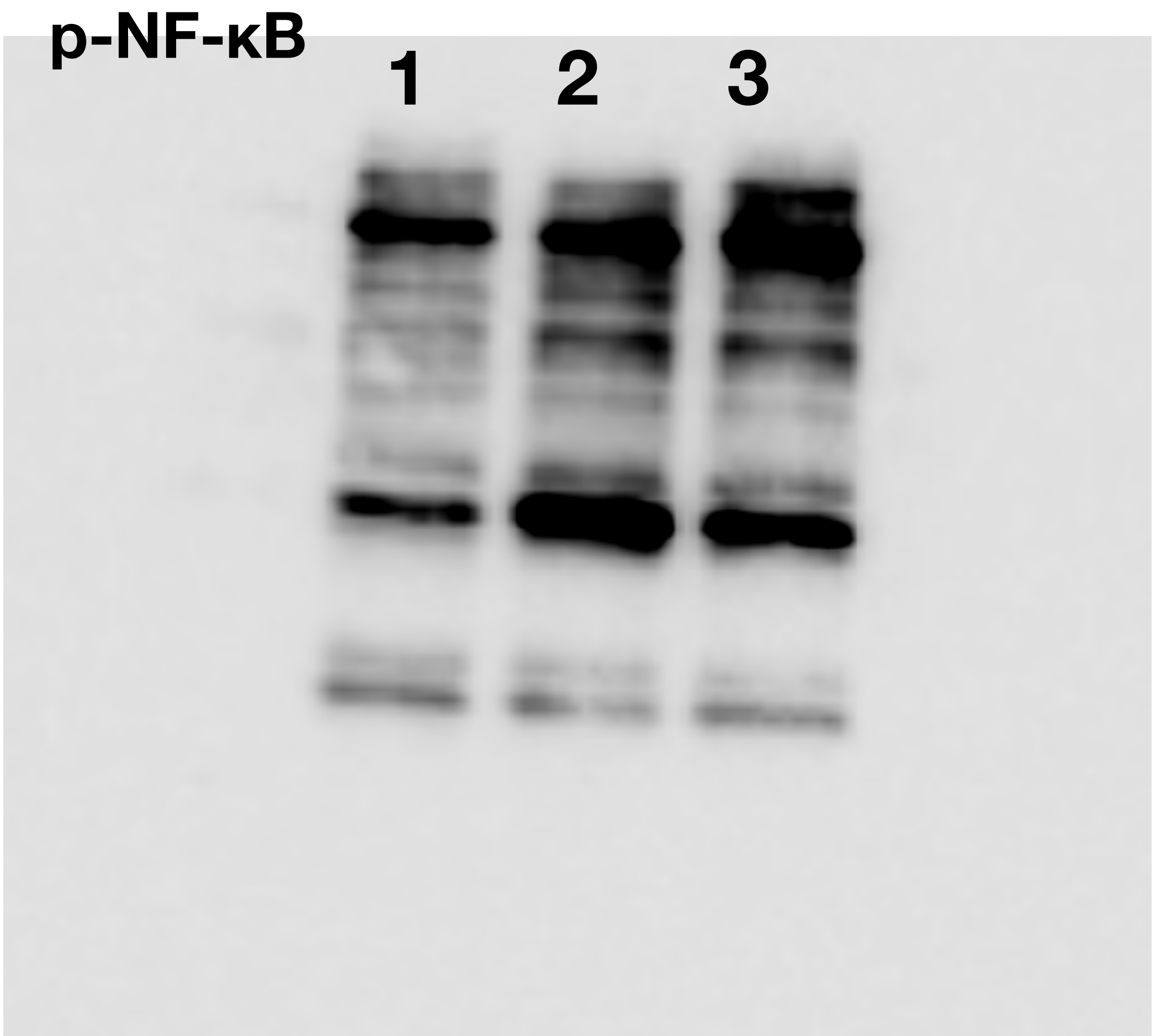

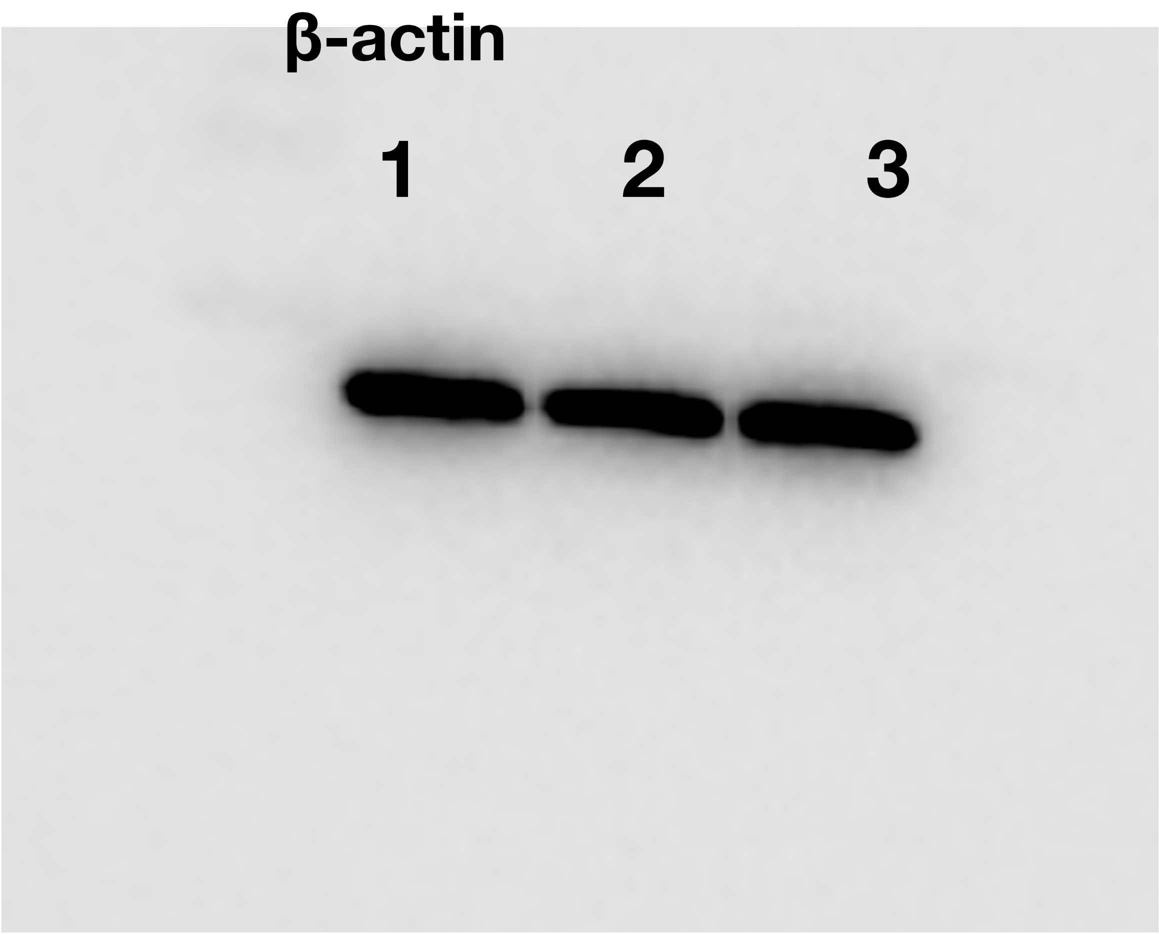

Western blot original bands from Figure 6D, Group 2 bands used for analysis

1 Control
2 LPS+control siRNA
3 LPS+MTA1 siRNA

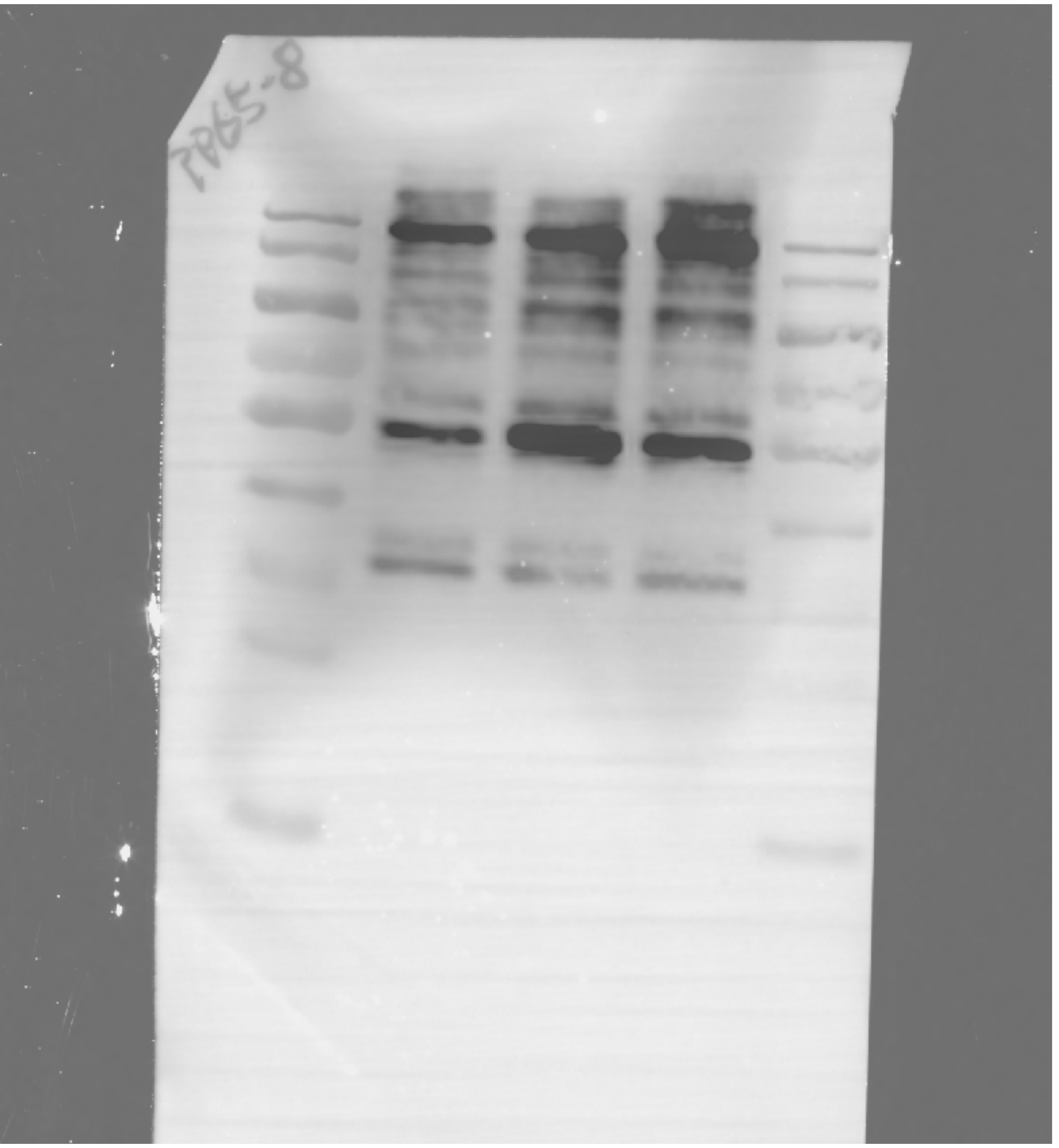

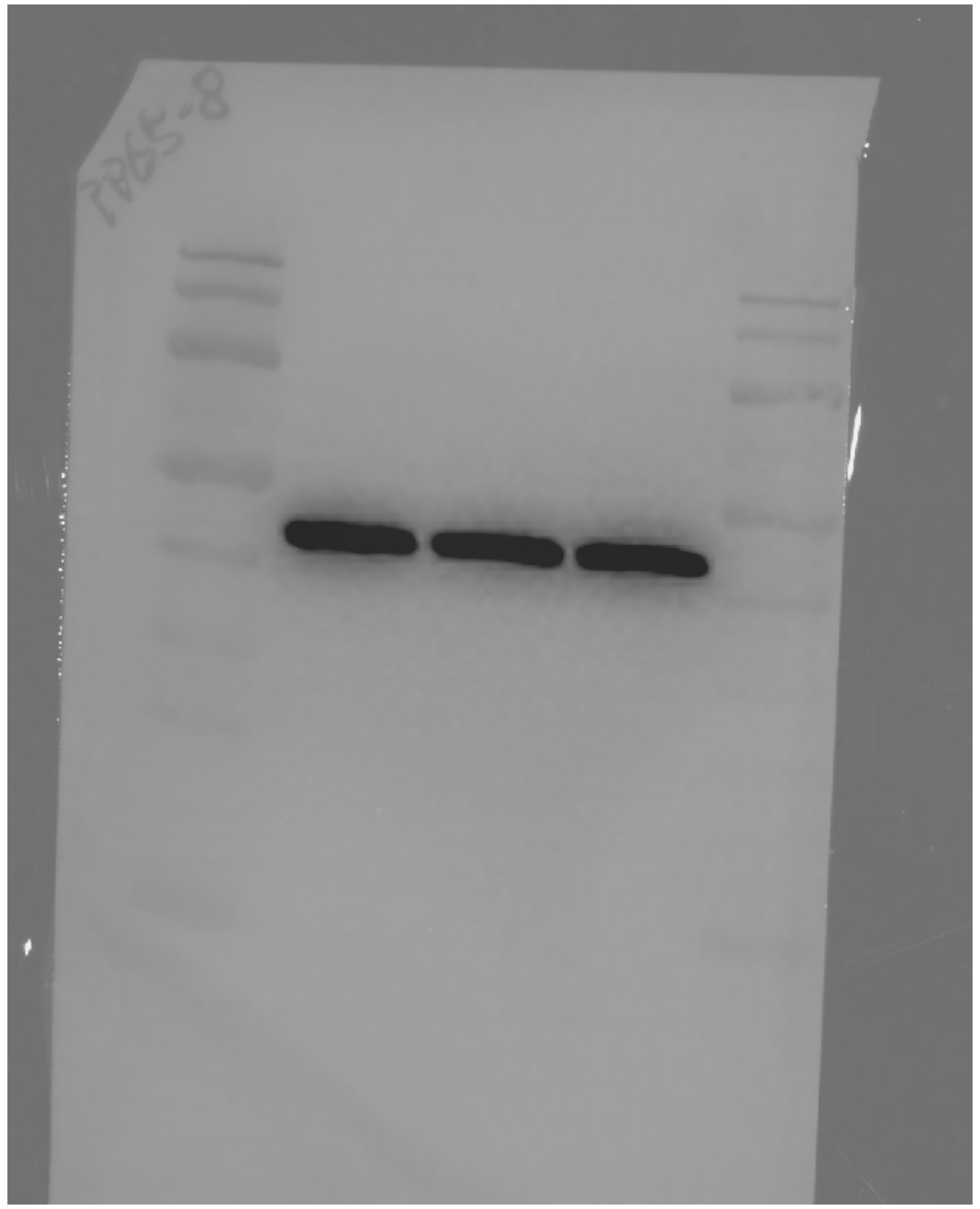

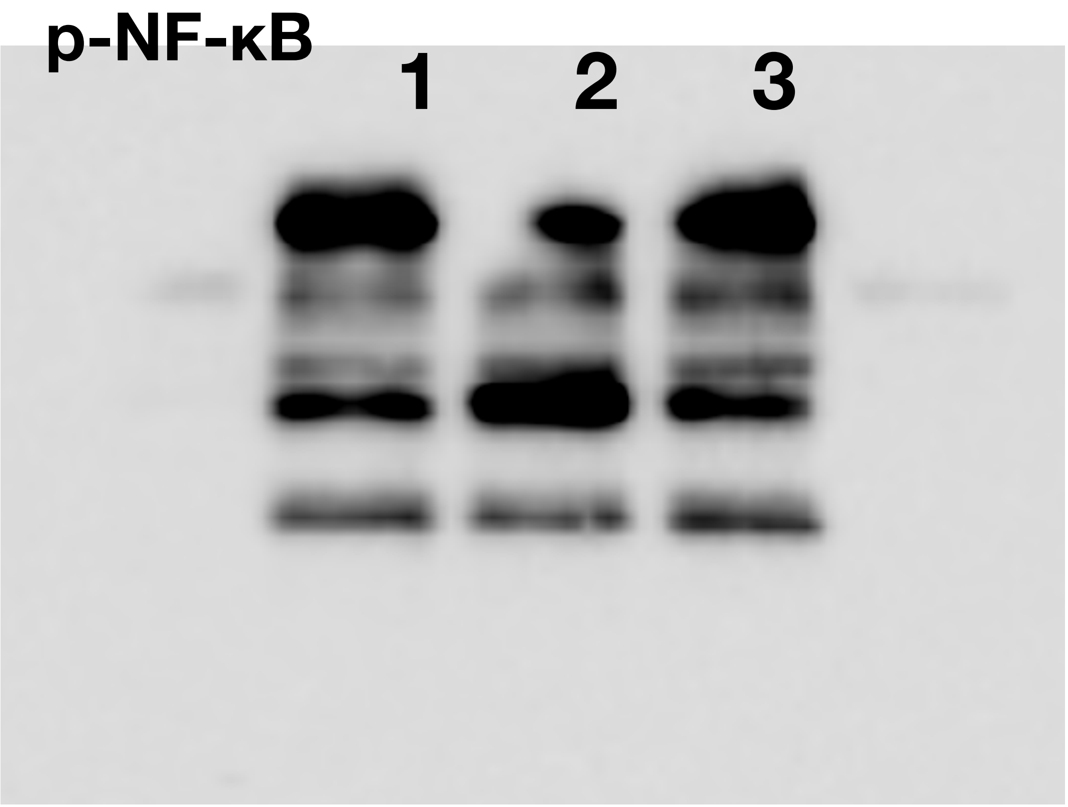

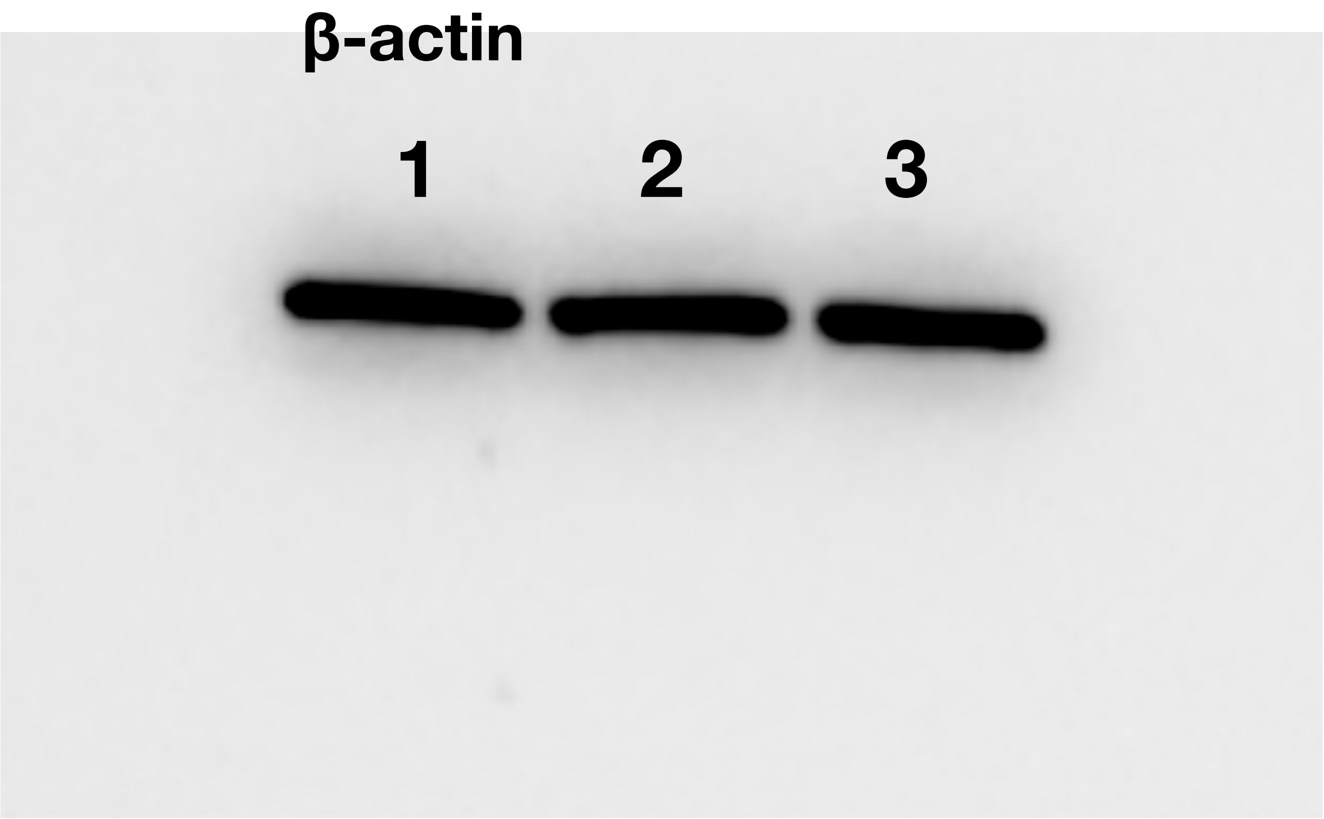

Western blot original bands from Figure 6D, Group 3 bands used for analysis

1 Control
2 LPS+control siRNA
3 LPS+MTA1 siRNA

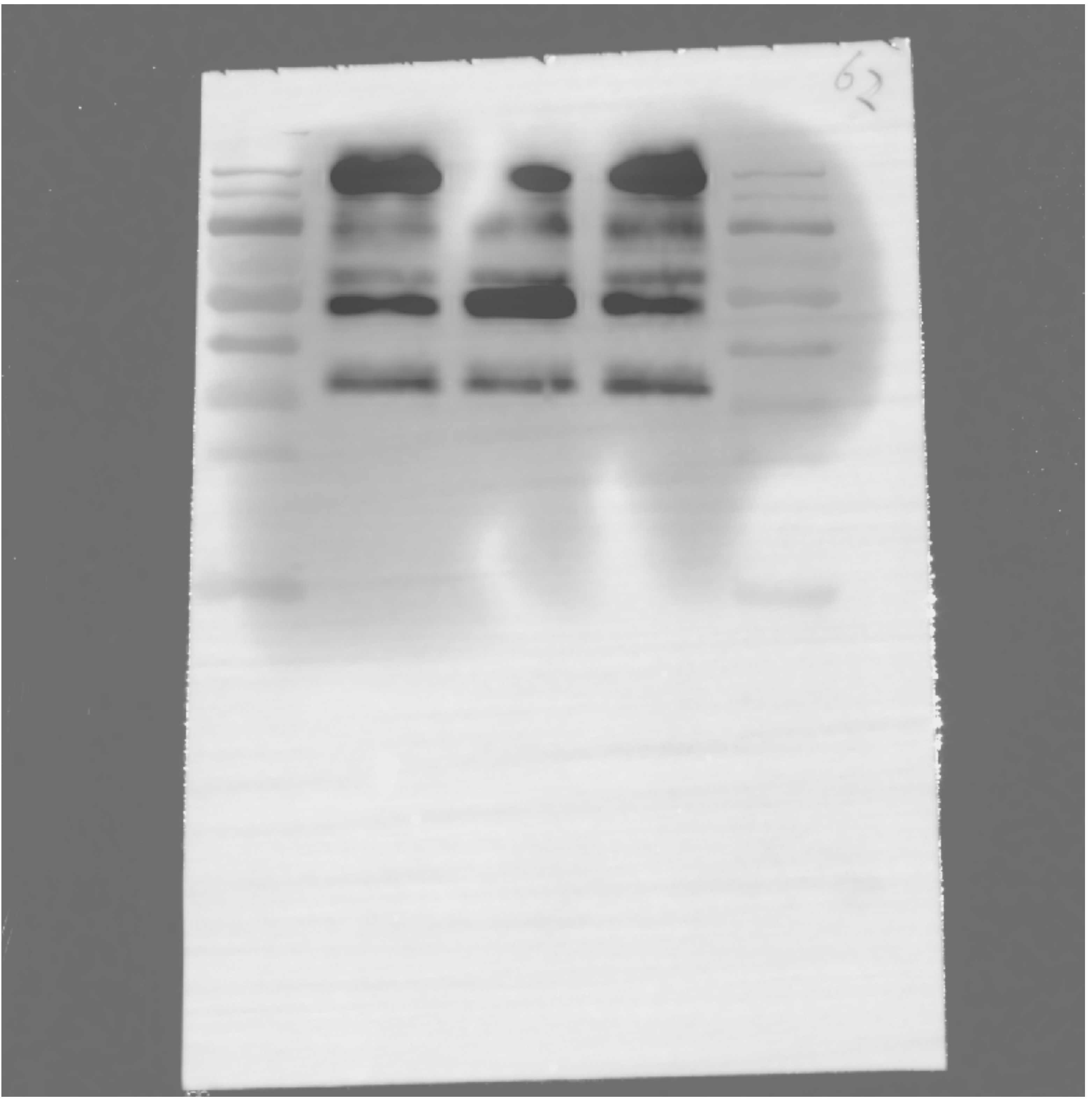

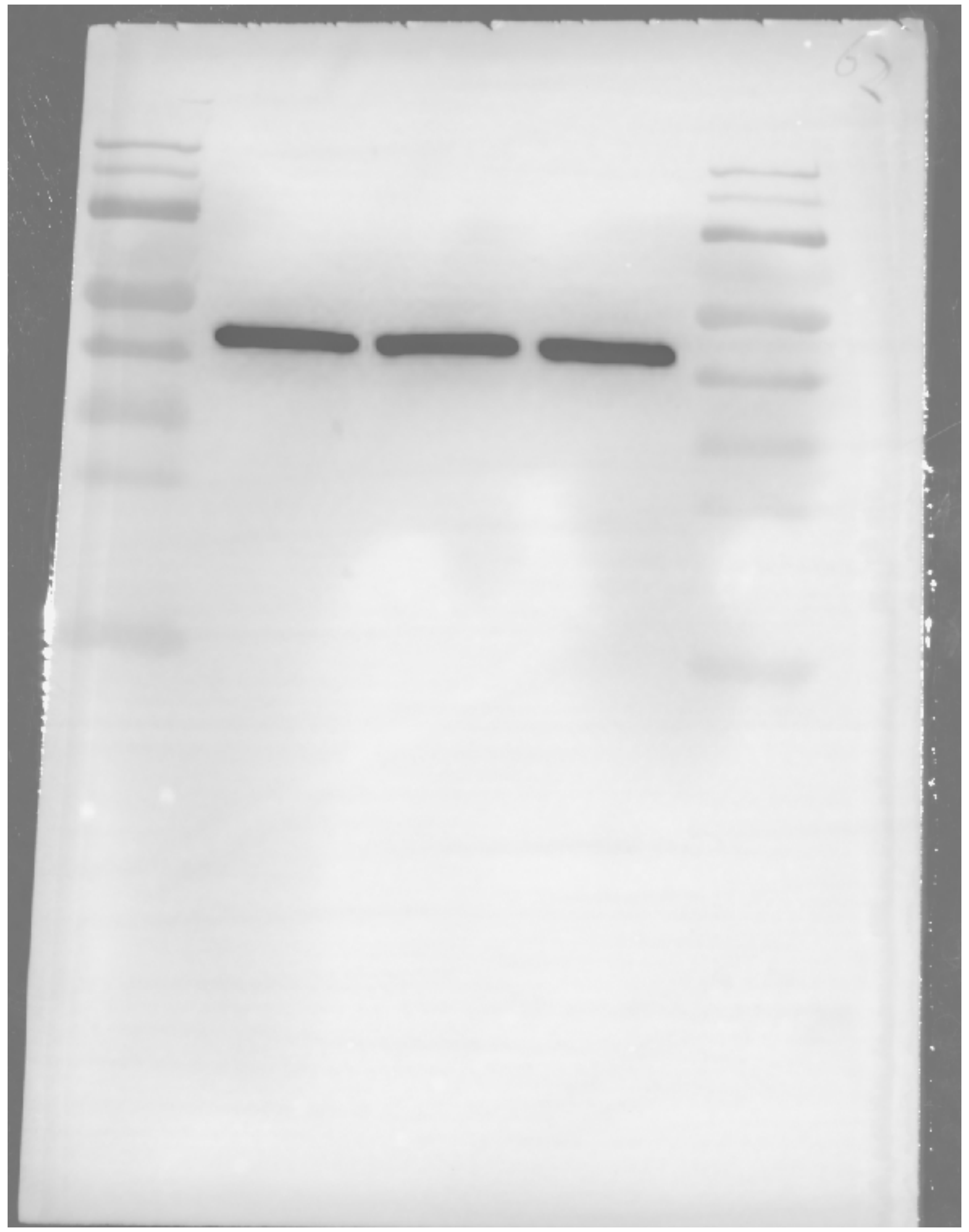

Supplement: Supplementary file 1 [file biomolecules-13-01495-s001.zip › Western blot original bands R5 .pdf]
